# Molecular Mechanisms of Intracellular Delivery of Nanoparticles Monitored by an Enzyme-Induced Proximity Labeling

**DOI:** 10.1007/s40820-023-01313-0

**Published:** 2024-02-01

**Authors:** Junji Ren, Zibin Zhang, Shuo Geng, Yuxi Cheng, Huize Han, Zhipu Fan, Wenbing Dai, Hua Zhang, Xueqing Wang, Qiang Zhang, Bing He

**Affiliations:** Department of Pharmaceutics School of Pharmaceutical Sciences, Peking University, 38 Xueyuan Rd, Haidian District, Beijing, 100191 People’s Republic of China

**Keywords:** Enzyme-induced proximity labeling, Intracellular delivery, Nano-protein interaction, Dynamic molecule profiling, Macrophages

## Abstract

Achieving increasingly finely targeted drug delivery to organs, tissues, cells, and even to intracellular biomacromolecules is one of the core goals of nanomedicines. As the delivery destination is refined to cellular and subcellular targets, it is essential to explore the delivery of nanomedicines at the molecular level. However, due to the lack of technical methods, the molecular mechanism of the intracellular delivery of nanomedicines remains unclear to date. Here, we develop an enzyme-induced proximity labeling technology in nanoparticles (nano-EPL) for the real-time monitoring of proteins that interact with intracellular nanomedicines. Poly(lactic-co-glycolic acid) nanoparticles coupled with horseradish peroxidase (HRP) were fabricated as a model (HRP(+)-PNPs) to evaluate the molecular mechanism of nano delivery in macrophages. By adding the labeling probe biotin-phenol and the catalytic substrate H_2_O_2_ at different time points in cellular delivery, nano-EPL technology was validated for the real-time in situ labeling of proteins interacting with nanoparticles. Nano-EPL achieves the dynamic molecular profiling of 740 proteins to map the intracellular delivery of HRP (+)-PNPs in macrophages over time. Based on dynamic clustering analysis of these proteins, we further discovered that different organelles, including endosomes, lysosomes, the endoplasmic reticulum, and the Golgi apparatus, are involved in delivery with distinct participation timelines. More importantly, the engagement of these organelles differentially affects the drug delivery efficiency, reflecting the spatial–temporal heterogeneity of nano delivery in cells. In summary, these findings highlight a significant methodological advance toward understanding the molecular mechanisms involved in the intracellular delivery of nanomedicines.

**Supplementary Information:**

The online version contains supplementary material available at 10.1007/s40820-023-01313-0.

## Highlights

- Novel enzyme-induced proximity labeling technology in nanoparticles (nano-EPL).
- Nano-EPL enables dynamic molecular mapping of the intracellular delivery of nanoparticles in macrophages.
- Nano-EPL enables the elucidation of a comprehensive phagosome-centered timeline during the vesicular transportation of nanoparticles, revealing distinct organelle engagement and its differential impact on drug delivery efficiency.

**Supplementary Information:**

The online version contains supplementary material available at 10.1007/s40820-023-01313-0.

## Graphical abstract

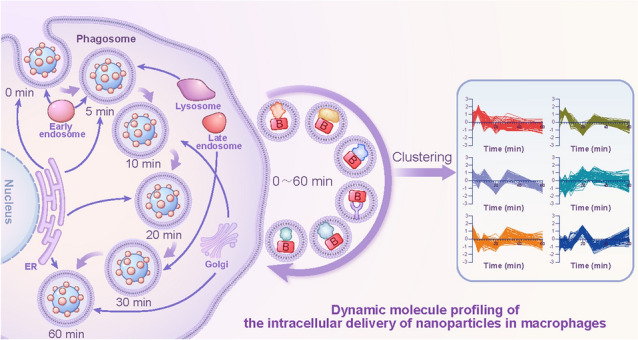

## Introduction

Nanomedicines significantly affect the absorption, distribution, metabolism, and excretion of loaded drug molecules [1]. When a drug is loaded in nanomedicines composed of different materials, the drug efficacy depends mainly on the biological behavior of the constituent materials in vivo [2]. Many nanomaterials confer different delivery and distribution characteristics on the loaded drug, such as reducing drug clearance in the kidney by changing particle size [3] or promoting drug penetration through the mucosa by facilitating transcellular transportation [4, 5]. In recent decades, numerous nanomaterials have been used to construct delivery systems, promoting the development of nanomedicines. However, the biological behavior of many nanomedicines remains unclear, although their potential efficacy has been demonstrated. Furthermore, how nanomaterials affect drug delivery is likewise not fully understood. These unelucidated mechanisms limit the clinical translation of nanomedicines [6, 7].

Generally, the delivery of nanomedicines needs to be targeted more finely, from organs to tissues, then to cells, and even to intracellular biomacromolecules. The delivery of nanomedicines in organs and tissues has been widely studied, and multiple mechanisms have been elucidated [8, 9]. As an example, when nanomedicines are administered through intravenous injection, the adsorption of plasma proteins as coronas and the resulting recognition by the reticuloendothelial system are considered the key mechanisms affecting the delivery of nanomedicines in vivo [10]. Therefore, identifying these corona proteins provides an important reference for the screening of efficient nanomedicines at the molecular level. Nevertheless, in terms of the cellular delivery of nanomedicines, although vesicular transportation from endosomes to lysosomes has been demonstrated, the detailed molecular mechanism remains largely unknown [11]. Multiple cellular proteins have been demonstrated to participate in the delivery process. Reportedly, receptor integrin and protease molybdenum cofactor sulfurase were successively discovered to affect and alter the intracellular transportation of nanomedicines [12, 13]. However, the transportation of nanomedicines among different vesicles in cells is a complex and rapid process that occurs within minutes and involves many protein molecules. Based on current techniques, it is very difficult to clarify all the proteins that participate in the cellular delivery of nanomedicines [14]. The dynamics of these proteins over time are even more difficult to elucidate. These difficulties make it necessary to establish a new technology to detect interacting proteins during the intracellular delivery of nanomedicines in real time.

Macrophages, a class of innate immune cells derived from the mononuclear phagocyte system (MPS), are widely distributed in the lung, liver, spleen, and other organs to remove pathogens and cell debris and present internalized antigens to activate adaptive immunity. Owing to their diverse functions, macrophages act as a double-edged sword affecting the delivery and efficacy of nanomedicines [15]. On the one hand, macrophages readily recognize and phagocytose nanomedicines in the blood, decreasing drug delivery efficiency. The loaded drugs are gradually degraded by the enzymes in phagolysosomes following phagocytosis [16]. However, multiple tissue-resident macrophages can act as an important target of nanomedicines for immunoregulation. Nanomedicines are usually designed as vaccines to promote antigen processing and presentation by enhancing the escape capability of antigens from the enzymatic degradation of phagolysosomes [17, 18]. Notably, regardless of the positive or negative effect, the intracellular delivery of nanomedicines in macrophages always requires the involvement of different organelles, including phagosomes, endosomes, lysosomes, the endoplasmic reticulum (ER), and the Golgi apparatus. Large numbers of proteins are engaged in transportation, affecting the intracellular fate of drugs by interacting with nanomedicines [19, 20]. However, the molecular characteristics of these interacting proteins remain largely unknown due to the lack of corresponding detection technology [21]. Furthermore, the molecular mechanism of nano delivery in macrophages has not been fully established thus far.

**Fig. 1:**
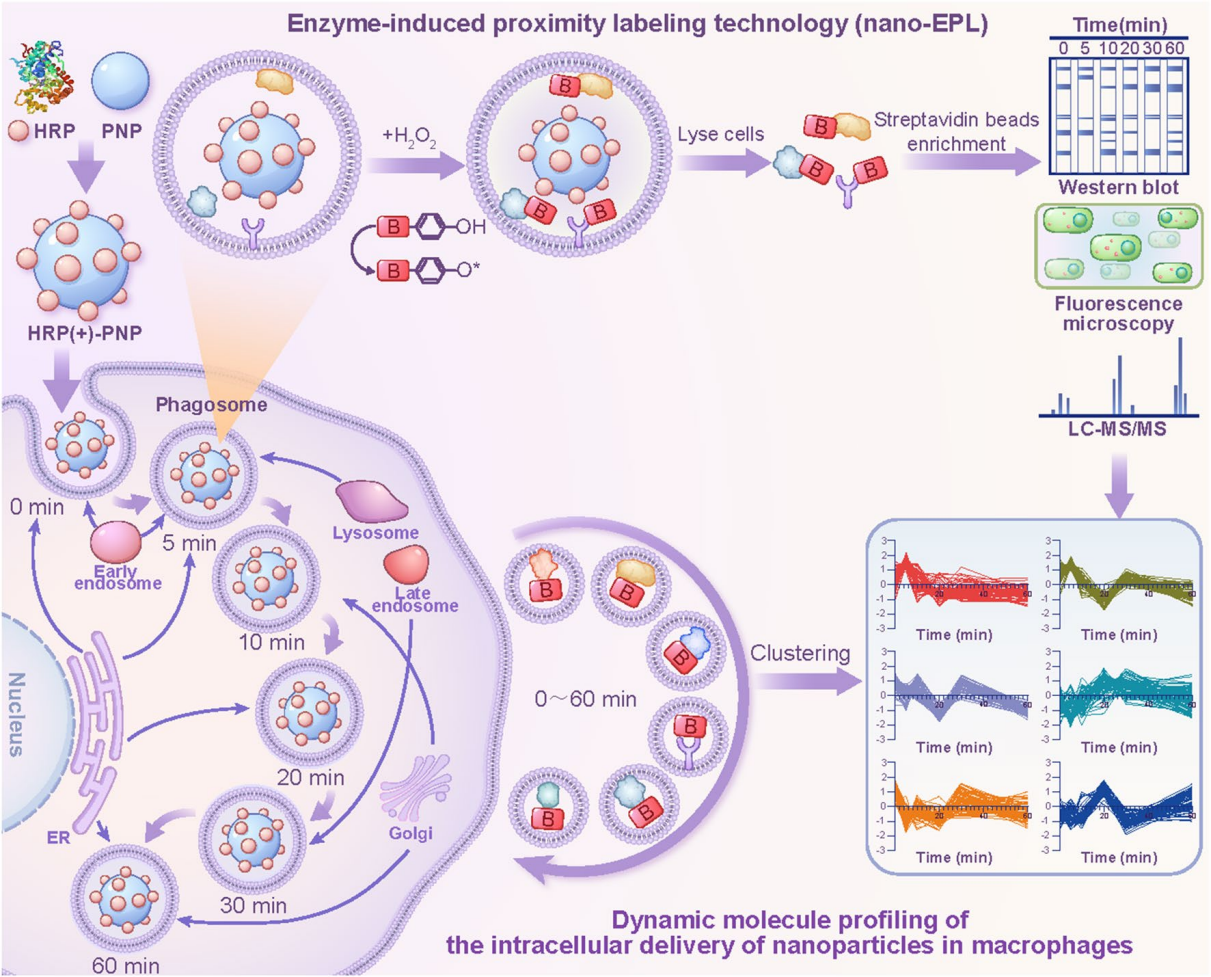
Schematic diagram of the construction of HRP (+)-PNPs and schematic representation of dynamic molecular profiling of the intracellular delivery of nanoparticles in macrophages by enzyme-induced proximity labeling technology (nano-EPL)

In this study, we developed an enzyme-induced proximity labeling technology in nanoparticles (nano-EPL) for the real-time monitoring of proteins that interact with intracellular nanomedicines in macrophages. As shown in Fig. 1, poly(lactic-co-glycolic acid) (PLGA) nanoparticles were prepared and coupled with HRP to construct the nanomedicine model (HRP(+)-PNPs) with EPL labeling capacity. The J774A.1 cell line was selected as the macrophage model and preincubated with the labeling probe biotin-phenol (BP). HRP (+)-PNPs were then added to the culture medium to induce phagocytosis by macrophages. Hydrogen peroxide (H_2_O_2_) was added as the substrate of HRP at different time points during the intracellular delivery of nanoparticles to rapidly trigger the enzyme-catalyzed reaction. H_2_O_2_ was quickly reduced to H_2_O with the production of highly reactive free radicals. These free radicals were rapidly transferred to the labeling probe BP in cells to induce BP activation. Activated BP molecules could rapidly react with proximal proteins by coupling to tyrosine, serine, and threonine residues, resulting in the tagging of these proteins by the biotin in BP [22]. This enzyme-catalyzed reaction was a rapid and efficient process. The whole labeling reaction was completed within a few seconds. Moreover, due to the short half-life of activated BP molecules, they could only couple with the nearest proteins. This strategy was reported to enable the efficient labeling of proteins within a range of approximately 20 nm around the catalyzing enzyme [23]. Therefore, when HRP was coupled to the nanoparticle surface, only the adjacent proteins that interact with nanoparticles could be labeled by BP. In conclusion, with this technique, proteins interacting with nanoparticles could be tagged with BP at different time points during phagocytosis by macrophages. The biotinylated proteins were further isolated and purified by avidin-conjugated magnetic beads and identified and characterized by a proteomics strategy based on mass spectrometry. Different references were used throughout the intracellular delivery process. Finally, the dynamic protein profile during the intracellular delivery of nanoparticles in macrophages could be mapped. Based on the dynamic map, the detailed timeline of nanoparticle transportation at the molecular level could be further investigated and evaluated.

## Methods and Materials

### Materials

1,2-Distearoyl-sn-glycero-3-phosphoethanolamine polyethylene glycol 2000 (DSPE-PEG2000) was purchased from NOF Corporation (Tokyo, Japan). Chlorin e6 (Ce6) was purchased from Frontier Scientific. HRP was obtained from Amresco (CA, USA). L-ascorbic acid sodium salt, guaiacol, HRP-conjugated streptavidin, 4% paraformaldehyde, mounting medium, N-hydroxy succinimide (NHS), 1-Ethyl-3(3-dimethylaminopropyl) carbodiimide (EDC), phosphatidylcholines, egg (EPC), urea, Trolox, and the enhanced 3,3’-diaminobenzidine (DAB) substrate kit were obtained from Solarbio (Beijing, China). Dialysis membranes (MWCO: 200 kD) were obtained from Bestbio (Shanghai, China). Dithiothreitol (DTT) and immobilon western chemiluminescent HRP substrate were purchased from Merck Millipore (Darmstadt, Germany). Fluorescein isothiocyanate-dextran (FITC-dextran), Pluronic F68, and protease inhibitor cocktail were obtained from Sigma-Aldrich (Missouri, USA). Bovine serum albumin (BSA), BCA protein assay kit, cell counting kit-8 (CCK-8), PAGE gel silver staining kit, Coomassie blue fast staining solution, 4–20% SDS-PAGE gel, SDS-PAGE sample loading buffer, phenylmethanesulfonyl fluoride (PMSF), radio immunoprecipitation assay (RIPA), blocking buffer for western blot, prestained protein ladder (10–180 kD), the organelle-specific probes mentioned in this article, and DyLight 405-labeled goat anti-rabbit IgG (H + L) were obtained from Beyotime (Haimen, China). Antibodies used for immunofluorescence including rabbit anti-CD28, anti-EEA1, anti-LAMP1, and anti-ATP6V1D were purchased from Abcam (Cambridge, UK), anti-CD44, anti-STXBP2, anti-RAB11, anti-DYNLT1, anti-ECM29 were purchased from Proteintech (USA), anti-CORO7, and anti-GOLIM4 were purchased from Bioss (Shanghai, China). Recombinant mouse cathepsin B (CTSB) was purchased from ProSpec (Rehovot, Israel). Dynabeads™ MyOne™ Streptavidin T1 and Pierce 660nm protein assay reagent were purchased from Thermo Fisher Scientific (USA). Dulbecco’s modified Eagle’s medium (DMEM), penicillin/streptomycin, and phosphate-buffered saline (PBS) were purchased from Macgene (Beijing, China). Biotin-phenol (BP), biotin, and Cy5 amine were purchased from APExBIO (Texas, USA). Streptavidin-AF488 and rhodamine phalloidin were purchased from YEASON (Shanghai, China). Poly(D, L-lactide-co-glycolide), acid terminated, and lactide:glycolide 50:50 (MW 24,000–38,000, PLGA) were purchased Aladdin (Shanghai, China). PLGA_30k_-PEI_2000_ was obtained from Ruixi Biological Technology (Xi’an, China). Mouse macrophage cell line J774A.1 was obtained from National Infrastructure of Cell Line Resource (Beijing, China). Cells were cultured in standard conditions by using DMEM medium supplemented with 10% fetal bovine serum (FBS, Gibco) and 1% penicillin–streptomycin solution.

### Preparation of PLGA Nanoparticles (PNPs), HRP (±)-PNPs, Cy5-PNPs, Ce6@PPNs@pEGFP and Ce6@PPNs@siRNA

A solvent diffusion method was employed to synthesize PLGA nanoparticles (PNPs). PLGA was dissolved in acetone (0.25% w/v) and then slowly introduced dropwise into an aqueous phase containing a Pluronic F68 solution (1%, w/v). Subsequent to solvent evaporation (0.05 MPa, 60 °C, 20 min), the resulting mixture underwent centrifugation at 11,000 rpm for 10 min followed by resuspension, leading to the preparation of PNPs.

To achieve enzyme inactivation, HRP was exposed to a mixture of 50 mg L^–1^ HRP and 10 mM GSH. The mixture was incubated at 37 °C for 10 h, briefly heated for 10 min in a water bath at 100 °C, and subsequently purified through dialysis (MWCO: 3,500) overnight. The inactivated enzyme was then subjected to vacuum lyophilization, and the resulting product was re-dissolved to a concentration of 1 mg mL^−1^ in double-distilled water (ddH_2_O).

This process entailed incubating 0.2 μg mL^−1^ EDC and 0.15 μg mL^−1^ NHS with 1 μg mL^−1^ PNPs under gentle agitation (300 rpm). Following a 2 h incubation period, 50 μL of HRP (1 mg mL^−1^) was introduced into the mixture. The reaction was allowed to proceed overnight under constant agitation at 300 rpm, after which the product was purified by dialysis (MWCO: 200 kD) overnight. The resulting conjugated nanoparticles were subsequently subjected to centrifugation at 11,000 rpm for 10 min, re-dispersed in 1 mL of PBS, and stored in the dark at 4 °C. The supernatants of HRP (±)-PNPs, prepared using varying HRP concentrations (25–1,000 μg mL^−1^), were freeze-dried and then re-dispersed in 20 μL of ddH_2_O for detection of coupled HRP and free HRP through Coomassie staining and BCA protein quantification assays. A graphical representation of the primary formulation steps is presented in Fig. S1.

To prepare Cy5-labeled PLGA (Cy5-PLGA), a conjugation reaction involving Cy5-amine and PLGA was conducted utilizing the EDC-NHS coupling method. Specifically, 50 mg of PLGA, 1 mg of NHS, 2 mg of EDC, and 4 mL of dichloromethane (CH_2_Cl_2_) were combined in a two-necked flask and stirred at 4 °C overnight. After solvent evaporation, the resultant powder was re-dissolved in N, N-dimethylformamide (DMF) to achieve a concentration of 10 mg mL^−1^. Cy5 dissolved in DMF was gradually added dropwise to the mixture, and the reaction proceeded at 25 °C for 36 h. The resulting reaction mixture was then subjected to dialysis (MWCO: 3500) in methanol for 1 day, followed by dialysis in ethanol for 2 days and subsequently in water for 2 days. The purified Cy5-PLGA was freeze-dried and stored in the dark at 4 °C for future utilization. The formulation process for Cy5-conjugated PNPs (Cy5-PNPs) was the same as that of PNPs, with Cy5-PLGA and PLGA combined in acetone at a mass ratio of 200:1.

The method of double emulsion (W/O/W) was used to synthesize Ce6@PPNs. Briefly, PLGA_30k_-PEI_2000_ (10 mg) and Ce6 (500 μg) were dissolved in chloroform (2 mL), followed by the addition of 400 µL of ddH_2_O containing 4 mg CaCl_2_ (10 mg mL^−1^). Ultrasonication (Scientz, China) was employed to emulsify the mixture, creating a primary emulsion (50 W, 5 min). This primary emulsion was subsequently incorporated into a 4% w/v polyvinyl alcohol (PVA) solution (10 mL) and subjected to a second round of sonication (50 W, 5 min) to yield a W/O/W double emulsion. The chloroform was removed via agitation using a magnetic stirrer for 6 h, followed by centrifugation (11,000 rpm, 10 min) to isolate the Ce6@PPNs. The prepared Ce6@PPNs were stored at 4 °C for future use.

For the preparation of Ce6@PPNs@Fam-siRNA, 200 nM siRNA was mixed with an equal volume of 2 mg mL^−1^ Ce6@PPNs. For the preparation of Ce6@PPNs@eGFP plasmid, 4 μg eGFP plasmid was mixed with an equal volume of 2 mg mL^−1^ Ce6@PPNs.

### Characterization of Nanoparticles

The hydrodynamic diameter and zeta potential of nanoparticles were measured by a laser particle size analyzer (Malvern Zetasizer Nano ZS, Malvern). The morphology of nanoparticles was observed under transmission electron microscope (TEM) (JEM-1400 Plus, JEOL) and scanning electron microscope (SEM) (JSM-7900F, JEOL).

To ascertain the presence of a protein corona on the surface of the nanoparticles, whole cell extract protein was meticulously extracted by subjecting J774A.1 cells to lysis in RIPA buffer, while maintained on ice for a duration of 30 min. Following this, the supernatant was carefully collected subsequent to centrifugation at 4 °C, 3,000 rpm for 10 min. 1 mg mL^−1^ PNPs, as well as HRP (±)-PNPs, were subjected to individual incubation with the whole cell extract protein, also at a concentration of 1 mg mL^−1^. These interactions were meticulously conducted at 37 °C for a period of 3 h. Subsequent to the incubation, the samples underwent centrifugation at 11,000 rpm for 10 min, facilitating the collection of nanoparticle pellets, which were subsequently subjected to a rigorous triple wash with ddH_2_O. The protein corona associated with the nanoparticles was effectively extracted from the nanoparticle pellets through a process involving boiling the samples in 1X protein loading buffer for a duration of 10 min. Finally, the protein corona was set for silver stain analysis.

### Spectrophotometric Assay of HRP Activity

The evaluation of HRP (+)-PNPs activity was predicated on the conversion of guaiacol to tetraguaiacol. Firstly, a standard curve was calibrated with varying concentrations of HRP (0.01–0.1 μg mL^−1^). The ensuing reaction was conducted within a 1 mL PBS, incorporating 0.03 M of guaiacol and HRP concentrations spanning 0.01 to 0.1 μg mL^−1^. The initiation of the reaction was achieved through the addition of 1 mM H_2_O_2_ (the final concentration) for 1 min. The dynamic absorbance alterations of the samples at 470 nm were systematically tracked through employment of a UV–visible spectrophotometer (HITACHI, Japan). To accurately assess the enzymatic activity of HRP(±)-conjugated nanoparticles during the initial minute, an empirically determined linear segment of the activity curves was utilized. This calculated enzymatic activity was subsequently quantified as the relative mass of HRP. This rigorous methodology was further applied to elucidate the influence of the duration of NHS/EDC incubation on HRP enzymatic activity. Notably, the quantification of the quantity of HRP molecules conjugated onto the PNPs' surface was meticulously computed through integration of parameters including the HRP concentration present on the PNPs' exterior, the Avogadro constant, the average molecular weight of PNPs, the particle size, and the density of the PLGA matrix.

### Identification of the Labeling Reaction of HRP(+)-PNPs

25 μg mL^−1^ HRP (+)-PNPs or isolated HRP was incubated in PBS buffer supplemented with 500 μg mL^−1^ BSA and 500 μM BP at 37 °C for a duration of 1 h. The initiation of the reaction entailed the introduction of 1 mM H_2_O_2_ (the final concentration). Several negative control variants were included, encompassing samples without HRP, HRP (+)-PNPs, HRP (−)-PNPs, BP, and H_2_O_2_, respectively. The reaction was quenched after 1 min by the addition of a 100 X "quencher solution," comprising 1 M sodium azide, 1 M sodium ascorbate, and 500 mM Trolox in PBS. Subsequently, to the quenching step, each sample underwent a meticulous process for analysis. A volume of 4 μL from each sample was combined with 1 μL of 5X protein loading buffer and subsequently subjected to boiling at 100 °C for 10 min. The resultant boiled samples were promptly cooled on ice. Western blot analysis was then employed for all samples. To this end, 20 μg of protein from each sample was loaded onto a 4–20% gradient precast gel for SDS-PAGE, employing an electric field strength of 100 mV over a 90-min period. The proteins were subsequently transferred onto a polyvinylidene difluoride (PVDF) membrane at 4 °C, utilizing a voltage of 110 mV over 70 min. The ensuing step involved blocking the PVDF membrane with a western blot blocking solution at 37 °C for 30 min, followed by an incubation period of 2 h at 37 °C with HRP-conjugated streptavidin. The visualization of the PVDF membrane images was accomplished utilizing a Tanon 5200 multi-imaging system (Tanon, Shanghai, China).

### Effect of pH/CTSB/Lipid Membrane on the Labeling Reaction of HRP(+)-PNPs

To systematically investigate the impact of solution pH, distinct enzymatic environments, and lipid membrane interactions, a comprehensive series of assays was conducted.

For the evaluation of pH-dependent effects, HRP (±)-PNPs at a concentration of 1 mg mL^−1^ were subjected to incubation within PBS buffers spanning a pH range of 5.0 to 7.5. These buffers were supplemented with 500 μM biotin-phenol (BP) and 500 μg mL^−1^ BSA. Incubations were carried out at 37 °C over a period of 1 h. Additionally, to probe the effects of CTSB, HRP ( +)-PNPs at a concentration of 1 mg mL^−1^ were incubated in PBS buffer containing 25 μg mL^−1^ CTSB. This incubation, also at 37 °C for 1 h, served to delineate the enzyme's influence on the labeling reaction. The initiation of the labeling reaction was achieved through the introduction of 1 mM H_2_O_2_, and after 1 min, the reaction was quenched by the addition of a 100X "quencher solution."

Subsequent purification steps involved the utilization of 30 kD Amicon® Ultra centrifugal filters, employing 14,000 g for 10 min to eliminate free BP. The resultant samples were divided into two parts: one designated for western blot analysis and the other for silver stain analysis after the enrichment of biotinylated proteins through streptavidin beads.

The process of biotinylated proteins enrichment commenced with the thorough vortexing of 50 μL of streptavidin beads to ensure even dispersion. After two washes with RIPA, the beads were incubated with 400 μg of proteins from each sample for 1 h at room temperature on a rotator. Subsequent wash steps involved RIPA, 1 M KCl, 0.1 M Na_2_CO_3_, and 2 M urea in Tris–HCl (10 mM, pH 8.0), followed by additional washes with RIPA. Biotinylated proteins were eluted by boiling each sample in 1X protein loading buffer supplemented with 2 mM biotin for 10 min, with the eluate collected through magnetic separation. Separation and analysis of the samples were achieved through SDS-PAGE, followed by silver stain analysis utilizing a PAGE Gel Silver Staining Kit.

Furthermore, for an in-depth exploration of lipid membrane interactions, liposomes were meticulously prepared using the film-rehydration method. Specifically, a mixture of 16 mg EPC, 4 mg cholesterol, and 6 mg mPEG2000-DSPE was dissolved in chloroform and subsequently rehydrated with a 10 mg mL^−1^ BSA solution in PBS. The liposome solution underwent thorough rehydration via ultrasonication, followed by dialysis (MWCO: 200 kD) and quantification using the BCA assay kit. The resultant liposome pellets were resuspended and diluted to various BSA concentrations (0.175, 0.35, and 0.7 mg mL^−1^) in PBS.

To elucidate the effects of the lipid membrane on HRP (+)-PNPs labeling, blank liposomes were prepared alongside control groups, employing similar protocols but using PBS buffer with no BSA during the rehydration step. Blank liposomes were then mixed with BSA at the same concentration as the control groups. Subsequently, 0.1 mg mL^−1^ HRP (+)-PNPs were introduced into the samples and incubated at 37 °C for 1 h. The labeling reaction was initiated through the addition of 1 mM H_2_O_2_, followed by quenching with the 100X "quencher solution" after 1 min. The subsequent enrichment and analysis of biotinylated proteins were facilitated through streptavidin beads and silver stain analysis.

### Pulse-Chase and Continuous Incubation Proximity Labeling

The labeling reaction of HRP (+)-PNPs within J774A.1 cells was executed through two methodologies, encompassing a pulse-chase approach or a continuous incubation approach.

In the pulse-chase paradigm, J774A.1 cells were maintained in complete DMEM medium containing 1 mM BP at a temperature of 4 °C for a span of 30 min. Subsequently, 2 mg mL^−1^ PNPs or HRP (±)-PNPs were introduced into the medium and allowed to incubate for 30 min at 4 °C, followed by a 10-min period on an ice bath. The preexisting medium was then replaced, and the cells were subjected to the complete DMEM medium supplemented with 500 μM BP, initiating a sequence of incubation periods at 37 °C spanning 0, 5, 10, 20, 30, and 60 min, respectively.

Conversely, within the continuous incubation approach, J774A.1 cells were exposed to the complete DMEM medium with 1 mM BP at 4 °C for 30 min. Subsequent to this, incubation ensued with 1 mg mL^−1^ PNPs or HRP (±)-PNPs at 37 °C over time intervals, namely 0, 5, 10, 20, 30, and 60 min. The introduction of 1 mM H_2_O_2_ at each time point served as the pivotal event for initiating the labeling reaction.

Upon the lapse of 1 min, the cells were subjected to three cycles of thorough washing with the "quencher solution." For subsequent western blot analysis, the cells were lysed following stringent procedures. This lysis process encompassed collecting cells in PBS, centrifuging at 4 °C and 3000 g for 10 min to form cell pellets. These pellets were subjected to lysis through RIPA lysis buffer augmented with 1X protease inhibitor cocktail, 1 mM PMSF, and quenchers, all within the confines of an ice bath, persisting for a duration of 30 min. Subsequent to this, the lysates were collected by centrifugation at 15,000 g for 10 min.

To ensure precision in quantification, the protein content of each sample was meticulously assessed employing the Pierce 660 nm assay. The western blot procedure was diligently executed, abiding by the established protocol.

For the purposes of fluorescence imaging, the cells underwent fixation through exposure to 4% paraformaldehyde at room temperature for a duration of 15 min. After fixation, a series of meticulous steps were followed, including three rounds of washing with PBS. The fixed cells were blocked with immunostaining blocking solution at 4 ºC overnight, subsequent to being treated with 2 μg mL^−1^ streptavidin-AF488. Additionally, cellular F-actin was visualized through staining with rhodamine phalloidin, conducted at room temperature over a span of 30 min, while nuclei were similarly stained with Hoechst 33,342, executed at room temperature for 20 min.

The culmination of these meticulously orchestrated steps was documented through imaging facilitated by an LSM880 confocal laser scanning microscope (Zeiss, Germany).

### TEM Imaging of HRP (+)-PNPs Proximity Labeling in Cells

To investigate the phagocytosis of HRP (±)-PNPs within J774A.1 cells, a meticulously executed pulse-chase approach, devoid of the labeling process, was undertaken. The parameters adhered closely to the aforementioned methodology, underlining a commitment to precision and consistency. The chase duration for this particular experimental configuration was set at 60 min time point.

Post-incubation, the J774A.1 cells were diligently fixed using a solution of 4% paraformaldehyde, maintained at room temperature for a duration of 15 min. Subsequent to fixation, a thorough and systematic cleansing process was initiated, involving three successive washes with PBS. To facilitate cellular permeabilization, the cells were treated with 0.2% Triton X-100 for 5 min, followed once again by a series of three PBS washes. The cells were blocked with immunostaining blocking solution at 4 ºC overnight, followed by incubation at 4 ºC overnight with HRP-conjugated streptavidin.

The cells were then subjected to an incubation step utilizing an enhanced 3,3'-diaminobenzidine (DAB) substrate kit, executed in full compliance with the manufacturer's protocol. After osmification, dehydration, embedding, and sectioning treatments, the subcellular structures in cells were observed by TEM (JEM-1400 Plus, JEOL).

### Colocalization of Biotinylated Proteins and Proteins-Interest

The intracellular labeling of proteins within distinct subcellular compartments using HRP (+)-PNPs was conducted as delineated earlier. This was subsequently followed by the application of the pulse-chase methodology. Biotinylated proteins were visualized using a concentration of 2 μg mL^−1^ streptavidin-AF488. Sequentially, the cells were subjected to an overnight incubation at 4 ºC with specific primary antibodies anti-CD28 (1:200), anti-CD44 (1:200), anti-EEA1 (1:1000), anti-LAMP1 (1:500), anti-STXBP2 (1:500), anti-GOLIM4 (1:500), anti-ECM29 (1:500), anti-DYNLT1 (1:200), anti-CORO7 (1:200), anti-RAB11 (1:200), and anti-ATP6V1D (1:200), respectively. This incubation took place within a 1% BSA (w/v) PBS solution. Subsequent to the primary antibody incubation, a secondary antibody, goat anti-rabbit AF405 (1:500), was applied and allowed to incubate for 1 h at 37 ºC. Colocalization of biotinylated proteins and proteins-interest was observed by confocal laser scanning microscope. For the colocalization analysis, the ZEN colocalization tool was used (ZEN software, Carl Zeiss).

### Colocalization of HRP (+)-PNPs and Subcellular Compartments

J774A.1 cells were subjected to staining using distinct fluorescent organellar probes, namely ER-tracker, Golgi-tracker, 70 kD FITC-dextran, and Lyso-tracker, adhering to the procedural guidelines outlined by the respective manufacturer. Subsequently, employing the pulse-chase paradigm without labeling, the internalization of HRP(+)-PNPs within these cells was conducted. Imaging of the cellular processes was conducted utilizing an LSM880 confocal laser scanning microscope (Zeiss, Germany), capturing images at intervals of 2 min over a span of 1 h.

### Confocal Imaging of Intracytoplasmic 70 kD FITC-Dextran, Fam-siRNA or eGFP after Photodynamic Destruction of Phagosome Membrane

1 mg mL^−1^ Ce6@PPNs, Ce6@PPNs@Fam-siRNA, and Ce6@PPNs@pEGFP were phagocytosed by J774A.1 cells following the pulse-chase approach described above without labeling. For the identification of intracytoplasmic 70 kD FITC-dextran, cells were preincubated with 1 mg mL^−1^ 70 kD FITC-dextran for 1 h. After a pulse-chase treatment with Ce6@PPNs and irradiation for 30 s (200 mW cm^−2^) at 5, 10, 20, 30, 60 min, the cells were fixed with 4% paraformaldehyde at room temperature for 15 min and washed three times with PBS. For the identification of intracytoplasmic Fam-siRNA, after pulse-chase treatment with Ce6@PPNs@Fam-siRNA and irradiation, the cells were fixed and washed. For the identification of intracytoplasmic eGFP, Ce6@PPNs@pEGFP were phagocytosed by cells in the same way. The difference was, after the irradiation treatment at 5 time points, the cells were incubated at 37 °C for 12 h. Then, the cells were fixed and washed. The images were taken by an LSM880 confocal laser scanning microscope (Zeiss, Germany).

### Identification of Biotinylated Proteins Generated by Nano-EPL

Biotinylated proteins were first enriched as described above. All sample solutions were separated using 10% SDS-PAGE gels. Gel fragments containing protein bands were cut out and stored at 4 °C. The gel was mixed with 50 mM NH_4_HCO_3_: acetonitrile (ACN) (v:v = 1:1) at 37 ºC, washed twice with ACN, and dried. This mixture was incubated with 5 mM DTT at 45 ºC for 30 min, then washed twice with ACN, and dried. The obtained sample was treated with 11 mM IAA for 20 min, washed twice with ACN, and dried. Subsequently, 6.25 μg mL^−1^ trypsin was added to the samples and left at 4 ºC for 60 min, and at 37 °C overnight. 10% trifluoroacetic acid (TFA) was then added to stop the reaction. The obtained peptide mixture was extracted twice with 50% ACN/0.1% TFA (v:v) and lyophilized for subsequent identification. The obtained peptides were detected by LC–MS/MS (Mass Spectrometric) based on a label-free quantification (LFQ) strategy. In brief, peptides were first loaded on a C18 precolumn (Thermo Fisher Scientific, USA) and then separated by nano-LC–MS/MS. In the process of separation, the mobile phases A (H_2_O/TFA) and B (ACT/TFA), flow rate, and gradient were set as reported in our previous work. LTQ Orbitrap Velos pro or Q-Exactive HF (Thermo Fisher Scientific, MA, USA) was used for mass spectrometric analysis. A data-dependent collision-induced dissociation (CID) model was used for the acquisition of MS/MS spectra. The top 15 most intense ions were selected for MS/MS. MaxQuant software (version 1.5.6.0) was used for analyzing the raw data. The heatmap and Pearson correlation were obtained by Perseus (version 1.6.0, http://www.perseus-framework.org) through transformation, filtering, and imputation. The identification of different proteins was conducted using the UniProt Mouse database.

### Statistical Analysis

All data in this study were analyzed by GraphPad Prism 8.0.2 and the *R* language and presented as mean ± SD. The comparison between two groups was conducted as the unpaired Student’s *t*-test (two-tailed), and the *P* value was considered to be statistically significant if it was lower than 0.05. Specifically, * represented *P* value < 0.05, ** represented *P* value < 0.01, *** represented *P* value < 0.001, and **** represented *P* value < 0.0001.

### Data Availability

The mass spectrometry proteomics data have been deposited to the ProteomeXchange Consortium (http://proteomecentral.proteomexchange.org) via the iProX partner repository with the dataset identifier PXD043708.

## Results and Discussion

### Enzyme-Induced Chemocatalysis in HRP (+)-PNPs Triggers the Proximity Labeling of Proteins

**Fig. 2:**
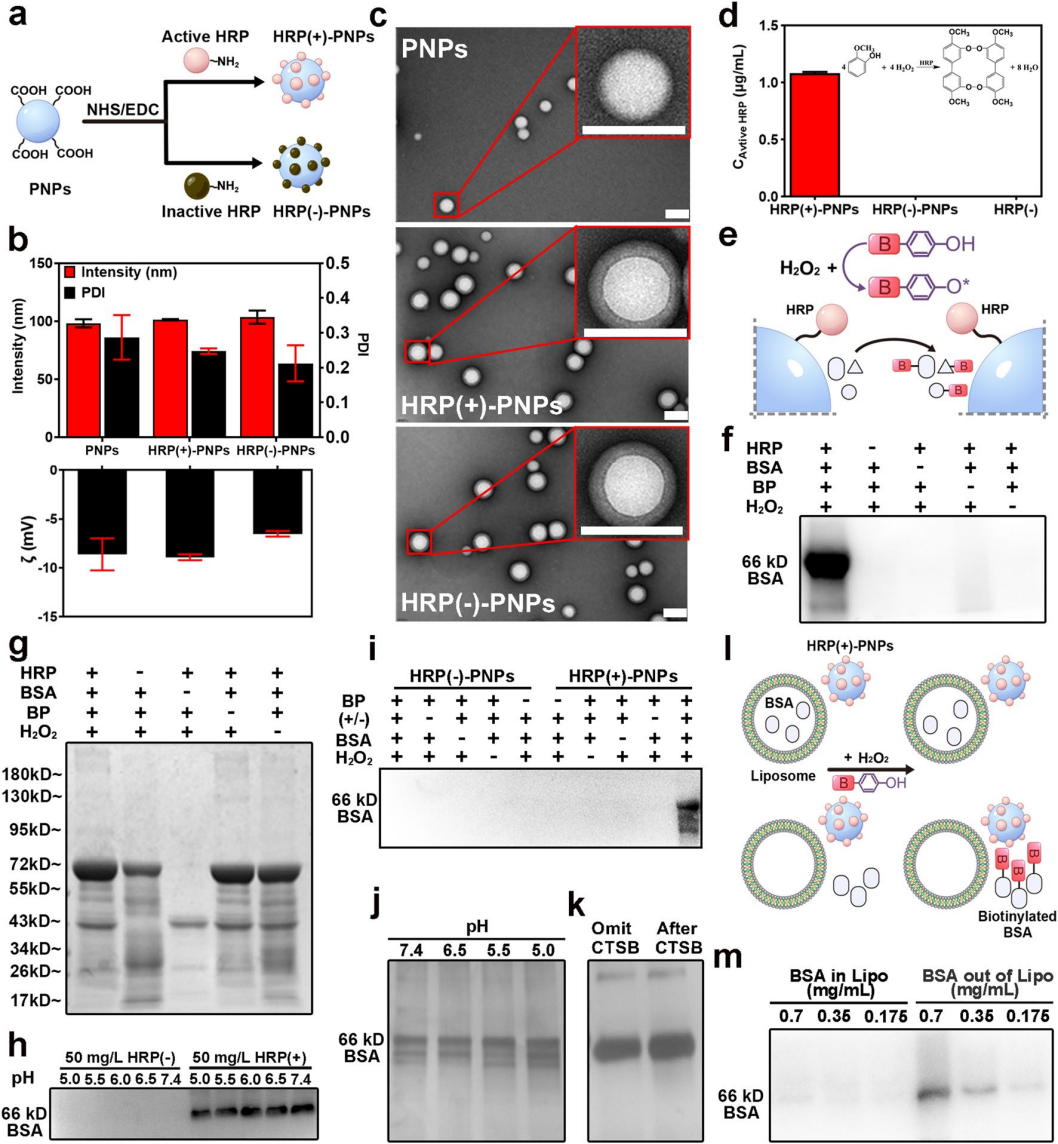
Preparation and characterization of PNPs and HRP (±)-PNPs.** a** Schematic illustration of HRP (±)-PNPs preparation. **b** Particle size and zeta potential value of PNPs and HRP (±)-PNPs (*n* = 3). **c** TEM images of PNPs and HRP (±)-PNPs (scale bar, 0.1 μm).** d** Relative enzyme activity of HRP (−), HRP (±)-PNPs. **e** Schematic illustration of the labeling activity of HRP (+)-PNPs. **g** Coomassie staining and **f** western blot of BSA labeled by HRP. **h** Western blot of BSA labeled by HRP ( ±) incubated in PBS at various pH values (5.0–7.4). **i** Western blot of BSA labeled by HRP ( ±)-PNPs. BP-omitted, H_2_O_2_-omitted, and BSA-omitted groups were used as controls. **j** Silver staining analysis of BSA labeled by HRP (±)-PNPs incubated in PBS at various pH values (5.0–7.4). **k** Silver staining analysis of BSA labeled by HRP (+)-PNPs incubated with CTSB for 1 h. **l** Schematic illustration of the labeling activity of HRP (+)-PNPs within/outside of liposomes. **m** Western blot of BSA labeled by HRP (+)-PNPs within/outside liposomes

First, PNPs were prepared by a solvent emulsification method. HRP was further conjugated to the nanoparticle surface via an EDC/NHS cross-linking reaction to establish a nanomedicine model with enzyme-induced proximity labeling capacity (HRP(+)-PNPs) (Fig. 2a). Here, the deactivated HRP obtained by glutathione (GSH) and boiling treatment was also coupled to the PNP surface, forming the inactive model (HRP(−)-PNPs) as the reference (Fig. S1).

According to measurement by dynamic light scattering (DLS) technology, both PNPs and HRP(±)-PNPs exhibited similar size distribution features (Fig. 2b). The mean diameter of all nanoparticles was approximately 100 nm, and the surface charge was negative at approximately -8.0 mV. The stability analysis in Fig. S2 revealed that all nanoparticles remained constant in particle size and dispersibility during incubation for 48 h. This guaranteed that the subsequent intracellular study was not interfered with by the stability of the nanoparticles. Transmission electron microscopy (TEM) and scanning electron microscopy (SEM) images further confirmed the size and morphological characteristics of the nanoparticles. As shown in Figs. 2c and S5, the coupling of HRP on the nanoparticle surface could be clearly distinguished by the formation of a corona structure. The activity assay demonstrated that the surface coupling of HRP did not affect the enzyme catalysis function (Fig. 2d). In contrast, the deactivated HRP on HRP (−)-PNPs completely lost its activity. Based on the quantitative catalysis detection using guaiacol as the substrate, we further calculated the coupling amount of HRP on each nanoparticle. As illustrated in Fig. S3a, the coupling amount of HRP was associated with its input in conjugation. When 100 μg mL^−1^ HRP was added to a 1 mg mL^−1^ PNP dispersion, an average of 15 HRP molecules were coupled to one PNP nanoparticle. Interestingly, while the concentration of HRP decreased to less than 50 μg mL^−1^, the coupling amount decreased to 10 molecules and remained unchanged. The amount of HRP coupled to each nanoparticle was limited, which suggested that these enzymes could not completely cover the nanoparticle surface. Consistent with this, the electrophoresis analysis of the interaction of nanoparticles with cellular proteins in culture medium showed that HRP (+)-PNPs and HRP(−)-PNPs had similar protein distributions to PNPs (Fig. S4), indicating that the coupling of HRP had no effect on the protein interaction with PNPs.

Next, we investigated the enzyme-induced labeling capacity of HRP proteins (Fig. 2e). According to western blot imaging using streptavidin as the specific indicator of biotin-tagged proteins, when HRP was directly dispersed in medium that contained BSA, only the coexistence of BP and H_2_O_2_ could induce the coupling of biotin to BSA (Fig. 2f), although the electrophoretic bands of BSA were also detected in the other treatment groups, as shown in Fig. 2g. Additionally, HRP exhibited a stable labeling capacity in different media from pH 5.0 to pH 7.4 (Fig. 2h). These findings fully demonstrated that HRP could be used as a catalyzing enzyme for the proximity labeling of proteins. Afterward, the labeling capability of HRP (+)-PNPs was further investigated using the same strategy (Fig. 2e). As illustrated in Fig. 2i, when HRP (+)-PNPs were added to the BSA dispersion, they induced the biotinylation of BSA only when both BP and H_2_O_2_ were added. In contrast, the deactivated HRP (−)-PNPs had no labeling capacity even when BP and H_2_O_2_ were both present in the medium. Notably, HRP (+)-PNPs also showed labeling stability in media with different pH values (Fig. 2j). Since the cellular uptake of nanoparticles was generally attributed to endocytosis or phagocytosis, where nanoparticles were transported from different endosomes to the acidic lysosome, this result suggested that the labeling efficacy of HRP (+)-PNPs was not affected by the pH variation of different intracellular vesicles, including lysosomes. This ensured the analytical accuracy of the intracellular delivery dynamics of the nanoparticles. Moreover, we verified that the labeling capacity of HRP (+)-PNPs was independent of cathepsin B (CTSB), which is the predominant degrading enzyme in lysosomes (Fig. 2k). This demonstrated the resistance of EPL technology to enzymatic degradation.

Finally, we tested the spatial accuracy of labeling during the intracellular delivery of nanoparticles. To investigate whether the free radicals from enzymatic catalysis could be released from the membranes of endosomes or lysosomes that contained nanoparticles to cause inaccurate labeling, we fabricated BSA-loaded liposomes to mimic endosomal membranes, incubated them with HRP (+)-PNPs and added both BP and H_2_O_2_ to trigger the enzymatic catalysis reaction. As shown in Fig. 2l, if the generated free radical penetrated across the membrane, it could biotinylate BSA in liposomes. The western blot image in Fig. 2m revealed that BSA loaded in liposomes was hardly labeled with biotin. In contrast, BSA that dispersed outside liposomes was significantly biotinylated. This result revealed that the proximity labeling triggered by HRP (+)-PNPs was accurately restricted to the endosome or lysosome that contained nanoparticles.

In summary, these findings demonstrated the feasibility of nano-EPL technology for the analysis of dynamic molecular mechanisms during the intracellular delivery of nanoparticles.

### Enzyme-Induced Chemocatalysis Enables the In Situ Labeling of Interacting Proteins During the Intracellular Delivery of HRP(+)-PNPs

**Fig. 3:**
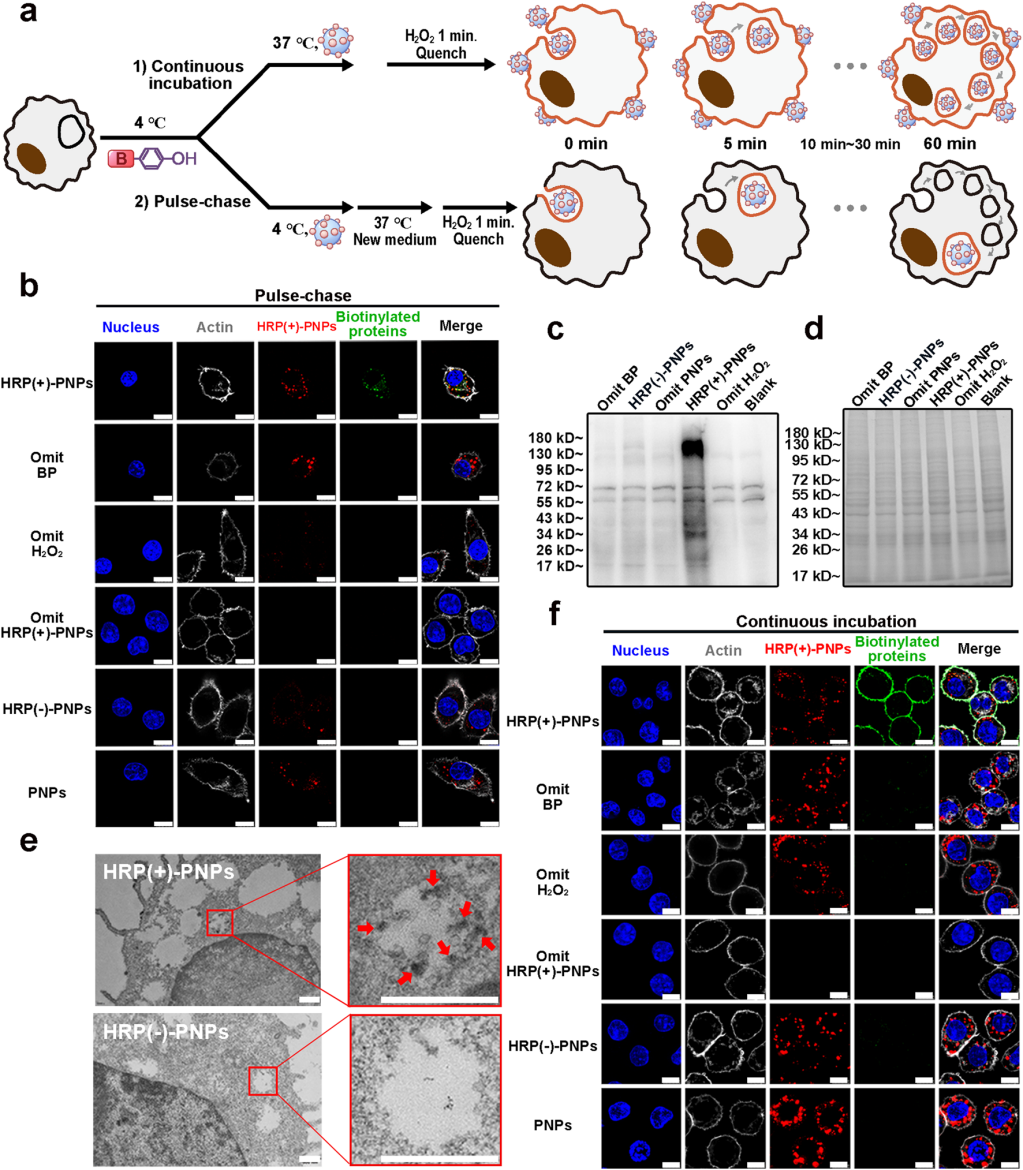
In situ labeling activity of interacting proteins during the intracellular delivery of HRP (+)-PNPs.** a** Schematic illustration of the two incubation methods. **b** Fluorescence images and **c** western blot and **d** Coomassie staining of intracellular proteins labeled by HRP (+)-PNPs through pulse-chase treatment. BP-omitted, H_2_O_2_-omitted, and BSA-omitted groups were used as controls (scale bar, 10 μm). **e** TEM analysis of proteins (as arrows show) labeled by HRP (+)-PNPs in phagosomes (scale bar, 0.5 μm). **f** Fluorescence images of intracellular proteins labeled by HRP (+)-PNPs through continuous incubation. BP-omitted, H_2_O_2_-omitted, and BSA-omitted groups were used as controls (scale bar, 10 μm)

The J774A.1 cell line was selected as the model of macrophages in this study, and the fluorescent probe Cy5 was conjugated to PNPs to trace the intracellular delivery of nanoparticles (Fig. S6a–c). Generally, the strategy of inducing intracellular delivery of nanoparticles was divided into two methods (Fig. 3a). Cells could be steadily incubated with the dispersion that contained nanoparticles, causing nanoparticles to continuously enter cells from outside. In addition, cells could be treated through a pulse-chase pattern to induce the intracellular delivery of nanoparticles. Cells were incubated with nanoparticles at low temperature for a short period to induce the adsorption of nanoparticles on the cell surface without triggering endocytosis or phagocytosis. Then, the nanoparticle dispersion was replaced by fresh culture medium, and the environmental temperature was reset to 37 °C to induce the internalization of adsorbed nanoparticles by cells.

We first investigated the intracellular labeling capacity of HRP (+)-PNPs in a pulse-chase incubation pattern. After staining biotinylated proteins with fluorescent streptavidin in the confocal images, as shown in Fig. 3b, revealed that intracellular HRP (+)-PNPs induced the biotinylation of proteins when pretreating cells with both BP and H_2_O_2_. The absence of either ingredient disabled the labeling capacity. More importantly, the biotinylated proteins colocalized well with Cy5-labeled nanoparticles, highlighting the proximity labeling capacity of EPL technology. In contrast, the deactivated HRP (−)-PNPs lost their catalytic and labeling functions, although the fluorescence of the nanoparticles themselves could be detected in cells. These findings confirmed the effectiveness of intracellular labeling, which was not interfered with by the complex protein environment in cells.

In addition, western blot imaging of biotinylated proteins was used to verify the labeling capacity of HRP (+)-PNPs. Only the coexistence of HRP (+)-PNPs, BP and H_2_O_2_ in cells could trigger the effective labeling of cellular proteins (Fig. 3c, d). By using streptavidin and diaminobenzidine (DAB) for the specific staining of biotinylated proteins in TEM imaging, it is shown in Fig. 3e that HRP (+)-PNPs markedly induced the deposition of black DAB granules. This further demonstrated the effective biotinylation of proteins. Notably, these biotinylated proteins were all restricted to the phagosome or lysosome. This result revealed the spatial accuracy of the proximity labeling triggered by HRP (+)-PNPs, which was consistent with the in vitro results shown in Fig. 2m.

The intracellular labeling characteristics of HRP (+)-PNPs in a continuous incubation pattern were further investigated in this study. As illustrated in Fig. 3f, the addition of both BP and H_2_O_2_ was also required for effective labeling. However, the colocalization image showed that the majority of biotinylated proteins were present in the cell membrane, but most nanoparticles entered the cells. This result suggested that HRP(+)-PNPs that remained outside cells might also participate in the labeling process during continuous incubation, thus causing the inconsistent colocalization between nanoparticles and biotinylated proteins. Finally, by comparing the effectiveness of intracellular labeling in two intracellular delivery patterns, the pulse-chase incubation strategy was demonstrated to reveal authentic nano-protein interactions in cells more accurately than the continuous incubation pattern.

### The Intracellular Delivery of HRP (+)-PNPs in Macrophages Is a Phagosome-Centered Process

**Fig. 4:**
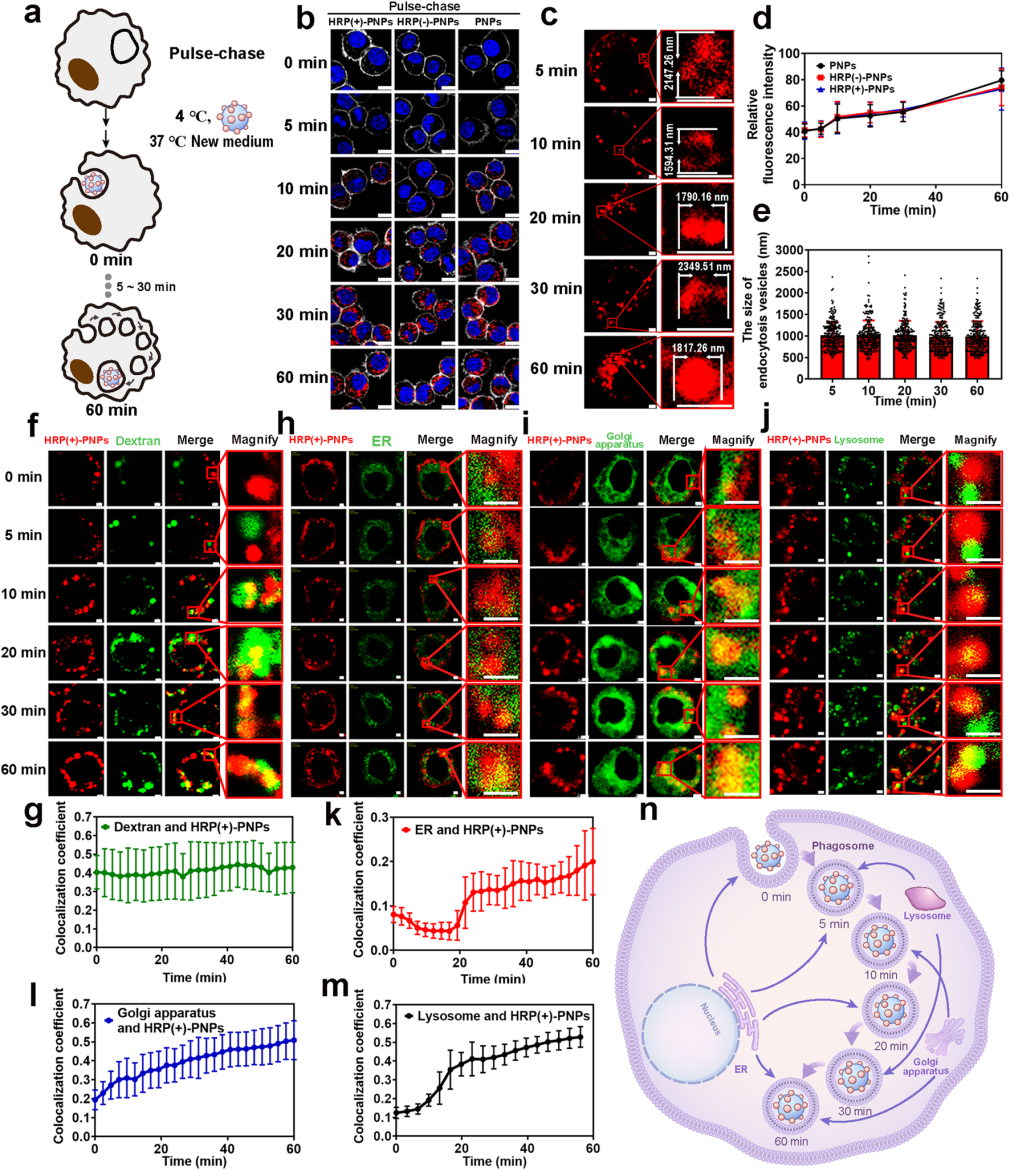
Phagosome-centered process during intracellular delivery of HRP(+)-PNPs.** a** Schematic illustration of the pulse-chase approach. **b** Fluorescence images and **d** quantitative image analysis of nanoparticles incubated with J774A.1 cells through pulse-chase treatment (scale bar, 10 μm). Nanoparticles (red), nuclei (blue), cytomembrane (white). **c** Fluorescence images and **e** quantitative image analysis of intracellular vesicles containing HRP(+)-PNPs at 5 time points (scale bar, 2 μm). Nanoparticles (red). **f** Florescence images of 70 kD dextran, **h** ER, **i** Golgi apparatus, **j** lysosome and HRP (+)-PNPs (scale bar, 2 μm). **g** Trend of colocalization assessed by regions between 70 kD dextran, **k** ER, **l** Golgi apparatus, **m** lysosome, and HRP (+)-PNPs over time (*n* = 50). **n** Schematic diagram of the fusion of phagosomes with different vesicles

Before clarifying the molecular mechanism of intracellular delivery using nano-EPL technology, we first investigated the vesicular delivery characteristics of nanoparticles in macrophages. Cy5-labeled nanoparticles were incubated with J774A.1 cells for different times in a pulse-chase pattern to induce stepwise transportation from the membrane to intracellular vesicles (Fig. 4a). As illustrated in Fig. 4b, nanoparticles gradually entered cells and were distributed in the form of fluorescent spots. This result demonstrated the vesicular delivery characteristics of the nanoparticles. The quantitative curves based on fluorescence intensity in Fig. 4d show that more nanoparticles were internalized by cells over time. Interestingly, HRP(+)-PNPs and HRP(−)-PNPs exhibited almost the same cellular uptake dynamics as PNPs. This result revealed that the coupling of HRP to PNPs had no effect on the biological behavior of the nanoparticles. Actually, the intracellular delivery of nanoparticles in a continuous incubation pattern also showed similar characteristics (Fig. S7). These findings fully verified that the intracellular delivery of nanoparticles relied on the vesicular pathway.

Notably, via high-resolution imaging based on confocal laser scanning microscopy (CLSM), we found that nanoparticles rapidly entered large vesicles with a range greater than 1 μm from the time of internalization (Fig. 4c). With increasing incubation time, more nanoparticles entered and filled these large vesicles. According to the statistical analysis, as shown in Fig. 4e, the mean diameter of these vesicles was approximately 1 μm, and the vesicular size was independent of the intracellular delivery of nanoparticles.

To determine the identity of these large vesicles, we used the classical fluorescent probe dextran to label phagosomes in macrophages. CLSM imaging in Fig. 4f shows that HRP (+)-PNPs rapidly entered the dextran-labeled phagosome. The colocalization analysis directly demonstrated that nanoparticles were quickly delivered to phagosomes and remained located there from the beginning of internalization (Fig. 4g). However, this process was not unchanging and constant. By labeling the ER, Golgi apparatus, and lysosome with the corresponding fluorescence trackers, it was illustrated in Fig. 4h–j that the vesicles from different organelles were continuously transported and fused to the nanoparticle-containing phagosomes. According to the colocalization analyses (Fig. 4k–m), lysosomes were transported to nanoparticle-containing phagosomes after 5 min of incubation, and the ER started to fuse 20 min later. In contrast, vesicles from the Golgi apparatus were continuously transported to phagosomes during the delivery of nanoparticles.

In summary, these findings suggested that the intracellular delivery of nanoparticles in macrophages was not a stepwise transportation among different vesicles. In contrast, as shown in Fig. 4n, nanoparticles were rapidly delivered to phagosomes from the beginning and continuously located there. The vesicles from other organelles were successively transported and fused to the nanoparticle-containing phagosomes. Therefore, although nanoparticles remained in the phagosomes, the microenvironment they encountered was continuously affected by the fused vesicles. Different proteins from these vesicles were transported to the nanoparticle-containing phagosomes and affected the intracellular fate of the drugs loaded in the nanoparticles.

### Nano-EPL Technology Achieves the Dynamic Molecular Profiling of Intracellular Delivery of HRP(+)-PNPs in Macrophages

**Fig. 5:**
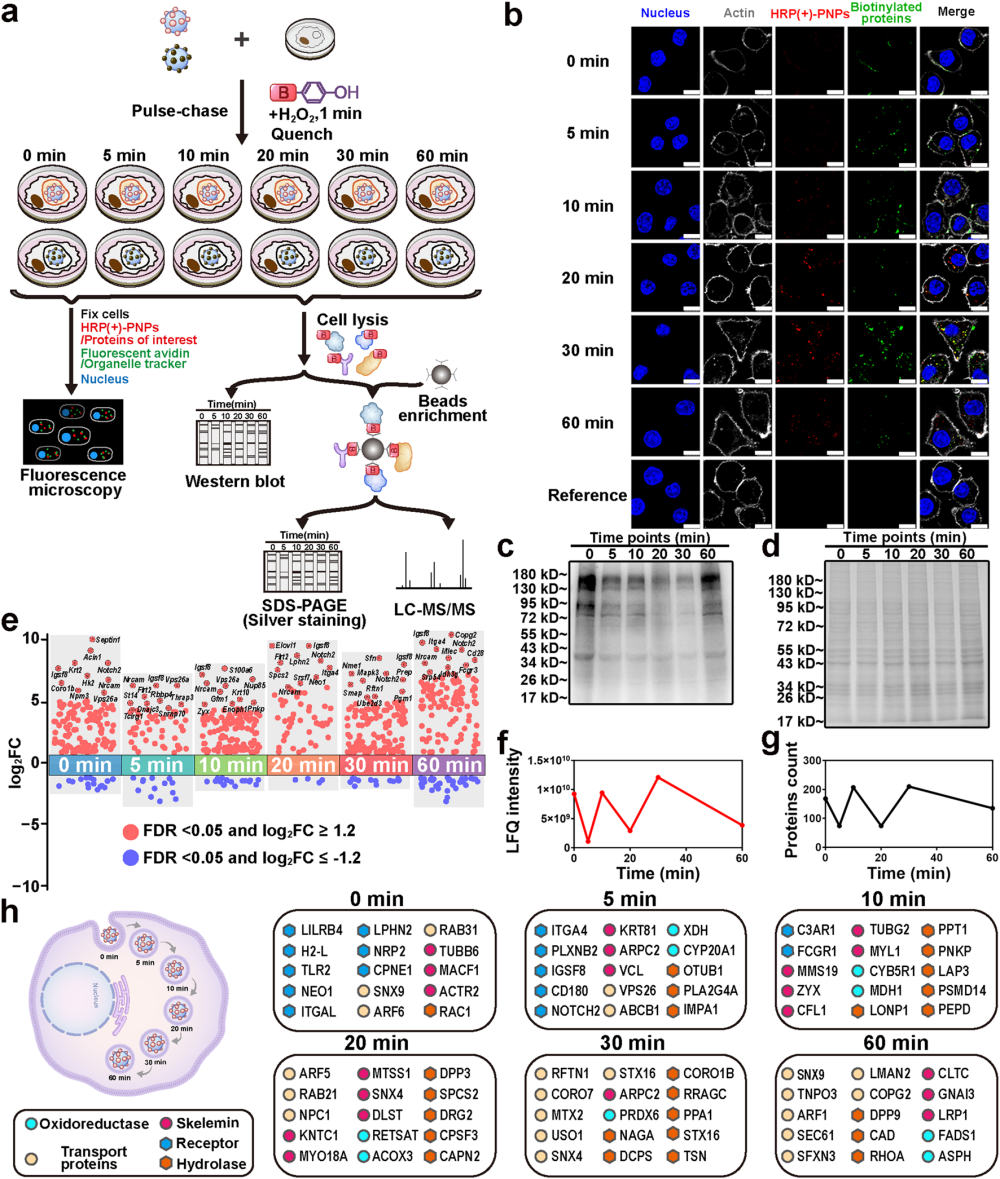
Dynamic molecule profiling of intracellular delivery of HRP (+)-PNPs in macrophages. **a** Schematic illustration of the proteomic experiment. **b** Fluorescence image and **c** western blot and **d** Coomassie staining of intracellular proteins labeled by HRP (+)-PNPs through the pulse-chase approach over time. (scale bar, 10 μm). **e** Manhattan plot for the true positive proteins at 6 time points. **f** LFQ intensity and **g** count of the true positive proteins of 6 time points. **h** Dynamic molecular mapping of intracellular delivery of nanoparticles

After demonstrating the phagosome-centered delivery pathway of nanoparticles in macrophages, we investigated the molecular mechanism using nano-EPL technology. HRP(+)-PNPs were incubated with J774A.1 cells in a pulse-chase pattern, and H_2_O_2_ as the substrate was added to the medium at different time points to trigger enzyme-induced labeling (Fig. 5a). Fluorescent streptavidin was then used to stain the biotinylated proteins. As shown in Fig. 5b, the dynamic distribution of nanoparticles exhibited high consistency with that of biotinylated proteins. All the fluorescently labeled proteins colocalized well with intracellular nanoparticles during the whole delivery process. This fully demonstrated the spatiotemporal accuracy of EPL technology in detecting the interacting proteins in the dynamic transportation of nanoparticles in cells. Western blot imaging of protein biotinylation in Fig. 5c further showed that different cellular proteins were biotinylated over time during the delivery of nanoparticles, although the content of total proteins was identical among different time points (Fig. 5d).

Next, to identify the interacting proteins at different delivery time points, a proteomics strategy based on LFQ using liquid chromatography‒mass spectrometry (LC‒MS/MS) technology was utilized in this study. According to the flow diagram in Fig. 5a, the biotinylated proteins were extracted after cell lysis, purified by streptavidin beads and then identified by LC‒MS/MS. To eliminate the negative effect of nonspecific labeling and endogenous biotinylation, HRP (−)-PNPs as the reference were incubated with macrophages for the same times as the treatment of HRP (+)-PNPs. H_2_O_2_ was also added to trigger enzyme-induced labeling, and the biotinylated proteins were extracted, purified and identified using the same procedure. The experiments that separately used HRP (+)-PNPs and HRP(−)-PNPs were performed twice at each time point. More than 1500 proteins were finally identified and quantified in each experiment (Fig. S9). Correlation analysis of the data showed that the Pearson coefficients among different samples were all greater than 0.800 (Fig. S10), demonstrating the feasibility and reproducibility of the proteomic strategy. Then, the ratio of protein abundance of HRP(+)-PNPs to HRP(-)-PNPs groups was used to plot the proteins frequency distribution (Fig. S11). For each protein, the abundance ratio (FC (±)) and the false discovery rate (FDR) were calculated according to the distribution diagram, and the corresponding scatter plot is illustrated in Fig. 5e. By setting the FDR as 0.05 and the FC (±) threshold as 1.2, 168, 74, 207, 74, 210, and 135 proteins were screened after treatment with HRP (+)-PNPs for 0, 5, 10, 20, 30, and 60 min, respectively. These proteins were regarded as the true positive proteins that interacted with nanoparticles during the delivery process. Notably, the dynamic curve of these proteins in the numbers showed a zigzag trend (Fig. 5f). The change in protein abundance based on LFQ detection also exhibited identical characteristics (Fig. 5g). These findings suggested that the microenvironment around HRP (+)-PNPs frequently changed during the intracellular delivery of nanoparticles. Furthermore, since nanoparticles were internalized and transported through a phagosome-centered delivery pathway in macrophages, the dynamic change in these interacting proteins highlighted the complex fusion process among endomembrane systems, including the phagosome, endosome, lysosome, ER, and Golgi apparatus.

To clarify the molecular mechanism of the dynamic transport of nanoparticles in cells, we investigated the function and location of these interacting proteins using gene ontology (GO) analysis based on the UniProt database. As shown in Fig. 5h, the identified proteins were differentially enriched at different time points during the delivery process of nanoparticles. By classifying and arranging these proteins in accordance with the function and participation time, we achieved dynamic molecular mapping of the intracellular delivery of nanoparticles (Fig. 5h and Table S2). Notably, many receptors, including leukocyte immunoglobulin-like receptor subfamily B member 4A (LILRB4), H-2 class I histocompatibility antigen (H2-L), toll-like receptor 2 (TLR2), integrin alpha-L (ITGAL), and adhesion G protein-coupled receptor L2 (LPHN2), were found to interact with nanoparticles from the beginning (0 min). This result suggested that surface binding mediated by multiple receptors was the trigger mechanism for nanoparticle internalization. However, the receptors that truly engaged in the endocytosis or phagocytosis of nanoparticles were limited because the number and proportion of interacting receptors were markedly reduced following nanoparticle internalization for only 5 min. Interestingly, they were enriched again after the transportation of nanoparticles for 60 min. This finding reflected the continuously changing microenvironment in phagosomes with time.

In addition to receptors, an increasing number of proteins involved in the motion and fusion of endosomes had been identified along with the intracellular delivery of nanoparticles. These proteins were generally divided into the cytoskeleton, including microtubule-actin cross-linking factor 1 (MACF1), tubulin beta-6 chain (TUBB6), and actin-related protein 2 (ACTR2); GTPases for vesicular fusion, such as tubulin gamma-2 chain (TUBG2), Ras-related C3 botulinum toxin substrate 1 (RAC1), and septin-8 (SEPTIN8); and structure and adapter proteins in endosomes, such as early endosome antigen 1 (EEA1), AP-2 complex subunit alpha-2 (AP2A2), and lysosome-associated membrane glycoprotein 1 (LAMP1) [24, 25]. With the aid of these proteins, endosomes and other vesicles were continuously transferred and fused to nanoparticle-containing phagosomes, causing variation in the microenvironment. Enzymes, especially proteases and hydrolases, including calpain-2 catalytic subunit (CAPN2), palmitoyl-protein thioesterase 1 (PPT1), and peroxiredoxin-6 (PRDX6), also gradually accumulate following nanoparticle internalization [26]. They cleaved and even degraded the internalized proteins, becoming the primary driver of the variation in the microenvironment around nanoparticles. In summary, the dynamic molecular profile of intracellular delivery of nanoparticles was built via nano-EPL technology. Proteins that interacted with nanoparticles at different delivery times were accurately identified, providing a series of potential targets for regulating the intracellular delivery of nanoparticles.

### Dynamic Molecule Profiling Based on Nano-EPL Technology Reveals a Detailed Organellar Participation Timeline During the Intracellular Delivery of HRP (+)-PNPs

**Fig. 6:**
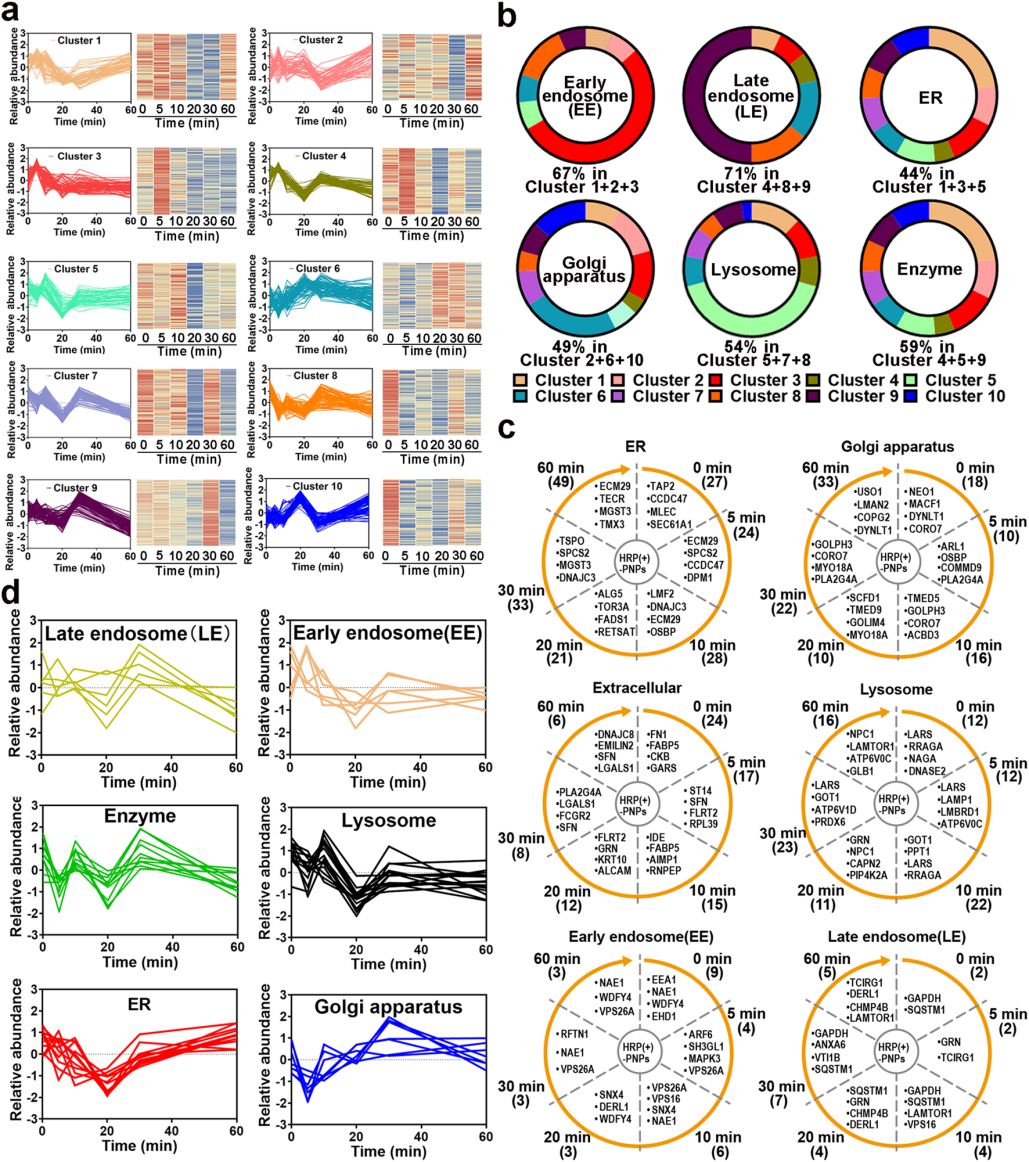
Proteomics analysis of the participating organelles. **a** Trend of proteins in each cluster after normalization. **b** The percentage of early endosomes (EEs), late endosomes (LEs), ER, Golgi apparatus, lysosome proteins, and enzymes in 10 clusters. **c** Trend of EEs**,** LEs, ER, Golgi apparatus**,** lysosome, and extracellular protein counts over time. Numbers in parentheses represent number of proteins. **d** Relative abundance trend of EEs, LEs, ER, Golgi apparatus, lysosome, and extracellular proteins over time

The intracellular delivery of nanoparticles in macrophages is a phagosome-centered process [19]. Nanoparticles are rapidly and continuously located in phagosomes after internalization. Endosomes, lysosomes, and ER/Golgi secreted vesicles are successively transported and fused to nanoparticle-containing phagosomes. However, the detailed transport and fusion timeline is unclear. Here, according to the established molecular profiling based on nano-EPL technology, we investigated the timeline of each interacting protein and conducted unsupervised clustering analysis in accordance with the change in protein abundance over time. As illustrated in Fig. 6a, the variation in different interacting proteins with time was divided into ten clusters. All the dynamic variations showed a zigzag trend but had different fluctuations from peak to trough over time. As an example, proteins in cluster 1 remained constant in abundance during the initial 10 min of internalization. Then, the protein abundance was markedly reduced in the next 10 min. Following the further extension of intracellular delivery, these proteins gradually increased again until 60 min. Proteins in cluster 3 exhibited a similar change feature as that in cluster 1, in which the abundance of all proteins was reduced to a minimum after the internalization of nanoparticles by macrophages for 20 min and then increased with the extension of time. However, unlike the slow-recovering feature in (cluster 1) 20 min later, the protein abundance in cluster 3 rapidly returned during the next 10 min. Notably, we found that nearly 44% of the ER-located proteins, including proteasome adapter and scaffold protein ECM29 (ECM29), dolichyl-phosphate-beta-glucosyltransferase (ALG5), microsomal glutathione S-transferase3 (MGST3), GPI transamidase component PIG-T (PIG-T), and all-trans-retinol 13,14-reductase (RETSAT), were mainly distributed in clusters 1 and 3 (Fig. 6b) [27]. More importantly, many structural proteins in the ER, such as ER membrane protein complex subunit 1 (EMC1) and PAT complex subunit CCDC47 (CCDC47), were also identified in these clusters [28]. These findings suggested that many ER-located proteins participated in intracellular delivery and interacted with nanoparticles at different time points. Furthermore, considering the similar behavior of these proteins over time, it was indicated that the involvement of the ER in the intracellular delivery of nanoparticles had an intrinsic timeline.

According to the variation in protein abundance and category shown in Fig. 6a, c, the ER participated in the transportation of nanoparticles in macrophages from the initial internalization. Interestingly, early endosomes (EEs) were also widely involved in phagocytosis at the beginning (Fig. 6c) because nearly half of EE-located proteins were also distributed in cluster 3, showing a similar trend to ER proteins. These results revealed that both the ER and EEs rapidly interacted and fused with the nanoparticle-containing vesicles in the initial stage of phagocytosis. However, the engagement of ER and EEs was not sustained for a longer time. After internalization for only 5 min, the proximity proteins that interacted with nanoparticles were gradually replaced by the proteins located in late endosomes (LEs) and lysosomes, especially various hydrolases and proteases (Fig. 6a–c). These proteins were mainly distributed in clusters 5 and 9, peaking at 10 min of internalization and then rapidly attenuating and remaining at low abundance. This tendency indicated that LEs and lysosomes interacted with the nanoparticle-containing phagosomes following the engagement of ER and EEs. Because many enzymes in LEs and lysosomes are transported to phagosomes, there is a significant reduction in proteins that interact with nanoparticles, including multiple receptors. Interestingly, accompanied by a duration of phagocytosis of more than 30 min, more proteins in the Golgi apparatus were identified to interact with nanoparticles. These Golgi-located proteins were mainly distributed in cluster 6. Notably, the ER-located proteins accumulated again with the extension of phagocytosis, showing an increasing trend similar to that of the Golgi-located proteins. These results revealed that the ER and Golgi apparatus finally interacted and fused with the nanoparticle-containing phagosomes during the late stage of intracellular delivery.

To test whether the variation in interacting proteins located in different organelles shown in the unsupervised clustering analysis could reflect the real timeline of different organelles interacting with the nanoparticle-containing phagosomes, we selected specific organellar proteins from the identified data based on the strict location annotation in GO and performed supervised clustering analysis. As illustrated in Fig. 6d, the variation in these specific proteins in EEs, LEs, ER, Golgi apparatus and lysosomes with time showed an identical tendency with the overall trend of these organelles from the unsupervised clustering analysis. The consistency between unsupervised and supervised clustering analyses fully demonstrated that the organelles had different participation timelines during the intracellular delivery of nanoparticles in macrophages.

**Fig. 7:**
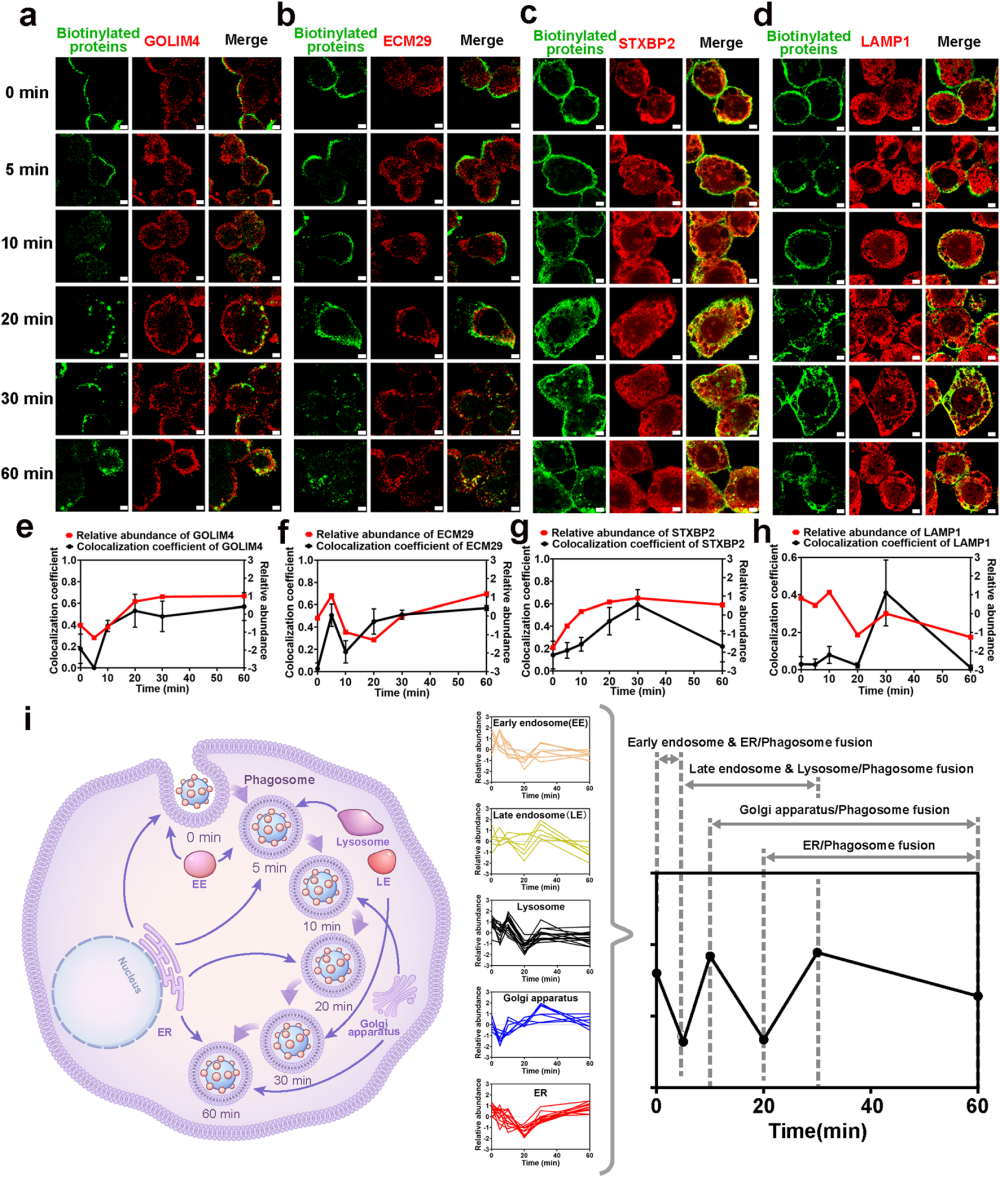
Detailed organellar participation timeline during the intracellular delivery of HRP(+)-PNPs.** a** Fluorescence images of GOLIM4, **b** ECM29, **c** STXBP2, **d** LAMP1 and biotinylated proteins (scale bar, 2 μm). Trend of colocalization assessed by regions and relative abundance between **e** GOLIM4, **f** ECM29, **g** STXBP2, **h** LAMP1 and biotinylated proteins over time (*n* = 50). **i** Schematic diagram of the dynamic subcellular organelles/phagosome fusion process during intracellular delivery of nanoparticles

Next, the participation timeline of different organelles was further verified by staining the specific organelle proteins with fluorescent probes and detecting their colocalization with the proximal proteins labeled by nano-EPL technology during the intracellular delivery of nanoparticles over time. First, GOLIM4, a classical marker of the Golgi apparatus, and STXBP2, which is involved in intracellular vesicle trafficking and vesicle fusion with membranes, were stained, and their colocalization with biotinylated proteins labeled by nano-EPL technology was investigated [29]. As illustrated in Fig. 7a, c, e, and g, the variation in colocalization coefficients with time showed a high degree of consistency with the abundance change in GOLIM4, STXBP2 in the proteomic analysis. Likewise, ECM29, a representative structural protein in the ER, and LAMP1, a specific marker of lysosomes, both exhibited consistency between the colocalization coefficient and protein abundance over time (Fig. 7b, d, f, and h). These findings confirmed that the timeline based on a series of proteomic analyses could reflect the real dynamics of different organelles participating in the intracellular delivery of nanoparticles.

In summary, according to dynamic molecule profiling based on nano-EPL technology, a detailed organellar participation timeline during the intracellular delivery of nanoparticles was established here. As shown in Fig. 7i, the organelles, including endosomes, lysosomes, ER and Golgi apparatus, had independent participation timelines, although the intracellular delivery of nanoparticles in macrophages was a phagosome-centered process. By cumulating the variation curves of the proteins from different organelles, the global change in all the interacting proteins exhibited a zigzag trend in abundance and category with time. The binding of nanoparticles to the cell membrane of macrophages triggers phagocytosis. Internalization caused a remarkable reduction in proteins that interacted with nanoparticles. Both the ER and EEs rapidly interacted and fused with nanoparticle-containing phagosomes in the initial stage (0–5 min). However, their involvement was not maintained for a longer time. The next stage was the involvement of LEs and lysosomes in the intracellular delivery of nanoparticles (5–30 min). The involvement of many enzymes in LEs and lysosomes reduced the interacting proteins again. However, with the extension of phagocytosis, the Golgi apparatus gradually interacted and fused with the nanoparticle-containing phagosomes (10–60 min). Interestingly, the ER was also involved again during the late stage of intracellular delivery (20–60 min). As a result, the involvement of organelles that had different timelines finally constructed a dynamic and complicated intracellular delivery process of nanoparticles.

### Engagement of Distinct Organelles Differentially Affects the Intracellular Delivery Efficiency of Gene Drug-Loaded Nanoparticles

**Fig. 8:**
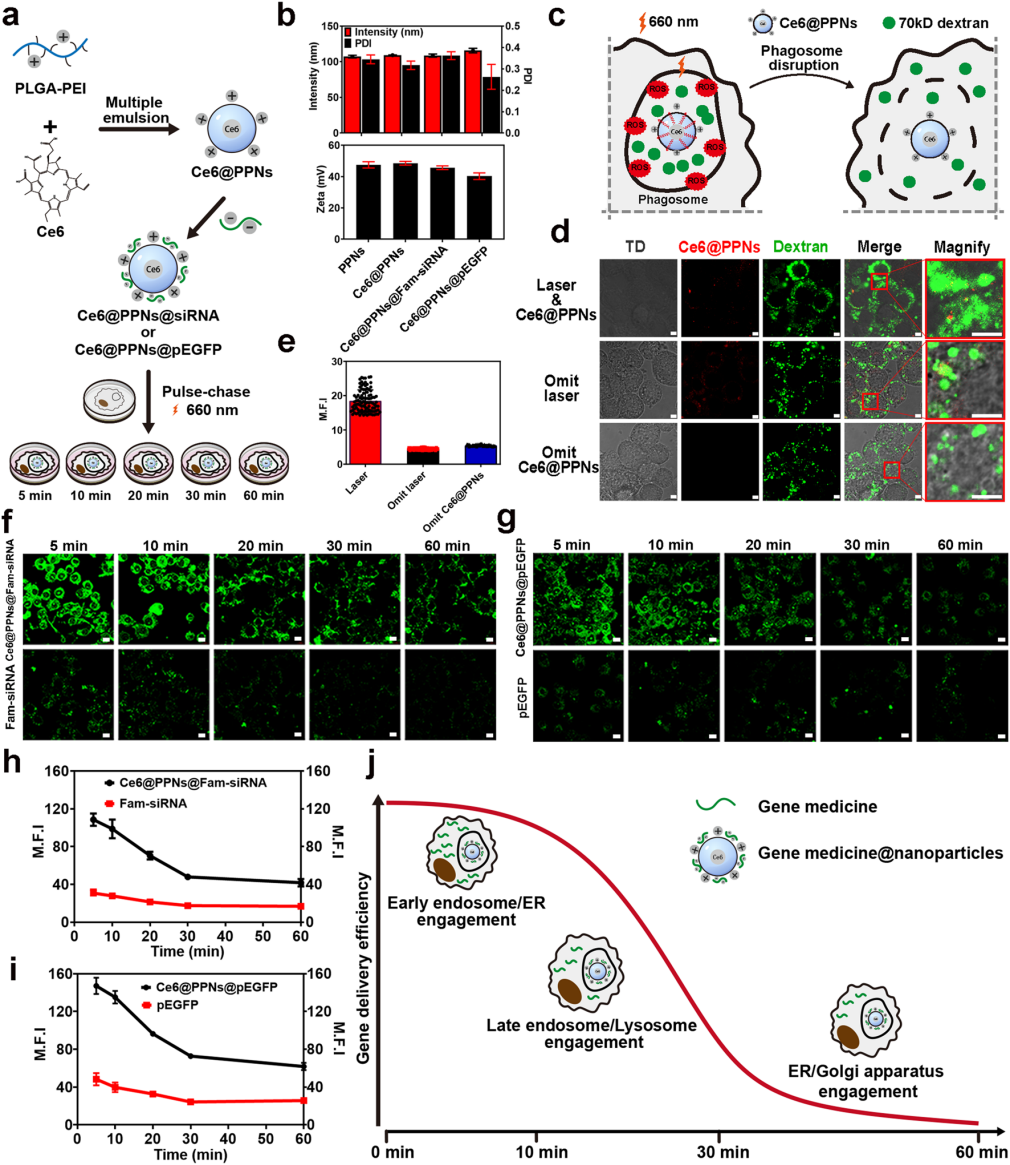
Intracellular delivery efficiency of gene drug-loaded nanoparticles. **a** Schematic illustration of Ce6@PPNs@siRNA and Ce6@PPNs@pEGFP preparation and functional steps. **b** Particle size and zeta potential value of PPNs, Ce6@PPNs, Ce6@PPNs@siRNA, and Ce6@PPNs@pEGFP (*n* = 3). **c** Schematic diagram of phagosome disruption caused by the photodynamic properties of Ce6@PPNs (660 nm, 200 mW cm^−2^, 30 s). **d** Fluorescence image analysis and **e** mean fluorescence intensity of intracytoplasmic 70 kD dextran after illumination. **f** Fluorescence images of cytoplasmic Fam-siRNA and **g** eGFP after photodynamic treatment at 5 time points (scale bar, 10 μm). Fam-siRNA and EGFP (Green). **h** Trend of mean fluorescence intensity of cytoplasmic Fam-siRNA and **i** eGFP after photodynamic treatment at 5 time points. **j** Schematic illustration of the optimum escape period

The involvement of different organelles in the intracellular delivery of nanoparticles is usually accompanied by the transportation of organellar proteins to nanoparticle-containing phagosomes [30]. These proteins, especially enzymes from different organelles, always cause alterations in the microenvironment around nanoparticles and even affect the intracellular delivery efficiency of loaded drugs. However, the detailed mechanism by which the engagement of different organelles affects the efficiency of intracellular drug delivery remains unknown. Here, to investigate the influence of different organelles on intracellular drug delivery, we selected PLGA-PEI as the carrier material to prepare nanoparticles (PPNs) and separately loaded two gene medicines, Fam-labeled siRNA and recombinant plasmid DNA expressing eGFP, as model drugs to monitor delivery efficacy (Fig. 8a). The introduction of PEI endowed PLGA nanoparticles with cationic characteristics. Two gene medicines could be efficiently loaded in nanoparticles via electrostatic interactions (Fig. 8b).

In this study, the dynamic molecule profiling based on nano-EPL technology, it was demonstrated in this study that different organelles had distinct timelines to participate in the intracellular delivery of nanoparticles. Therefore, to accurately evaluate the effect of different organelles on the delivery efficacy of gene medicines, we established a method for forcibly inducing the release of gene medicines from phagosomes into the cytoplasm at a specific time. As shown in Fig. 8c, chlorin e6 (Ce6), as a photosensitizer, was simultaneously loaded in PLGA-PEI nanoparticles while carrying gene medicines. When Ce6-loaded nanoparticles were internalized by cells for a specific time, the cells were exposed to a laser of 660 nm to trigger the production of free radicals, which could directly induce lipid peroxidation and cause the leakage of nanoparticle-containing phagosomes. Then, the loaded gene medicines could be completely released from phagosomes to the cytoplasm. Therefore, by detecting and comparing the loss of gene medicines in phagosomes when irradiating cells at different delivery timelines to trigger the complete release of gene medicines, the effect of different organelles on intracellular drug delivery could be accurately evaluated.

First, we tested the feasibility of this method to induce the leakage of nanoparticle-containing phagosomes. As illustrated in Fig. 8a, c, Ce6-loaded nanoparticles and FITC-labeled dextran, a classical phagosome probe, were simultaneously incubated with cells to trigger phagocytosis. After internalization for 30 min, the cells were then irradiated with a 660 nm laser to induce the leakage of phagosomes. According to the confocal imaging and the corresponding quantification results in Fig. 8d, e laser irradiation significantly led to the release of FITC-labeled dextran from phagosomes to the cytoplasm. As a result, this method could be used to compare the effects of different organelles on gene delivery efficacy by irradiating cells at different time points.

Next, we prepared PLGA-PEI nanoparticles that were separately loaded with Fam-labeled siRNA and recombinant plasmid DNA expressing eGFP. In accordance with the flow chart shown in Fig. 8a, nanoparticles were incubated with macrophages in a pulse-chase pattern. After internalization for 5, 10, 20, 30, and 60 min, the cells were exposed to a laser at 660 nm to trigger the complete release of the gene medicines. Then, the residual gene medicines and the gene expression product were detected by confocal imaging (Fig. 8f, g). Remarkably, when irradiation by laser was omitted, the intact structure of phagosomes caused tremendous gene medicines to be degraded, retaining little fluorescence of Fam-siRNA and eGFP in cells. In contrast, laser irradiation significantly promoted the delivery of gene medicines to the cytoplasm and accelerated gene expression by inducing the leakage of phagosomes. With the postponement of irradiation time points, both the residual siRNA and the gene expression product eGFP gradually decreased. It was indicated that many gene medicines were degraded before they were released from the leaked phagosomes. Notably, by quantifying the fluorescence intensity of Fam-siRNA and eGFP in cells after laser irradiation at different timelines, nonlinear curves were shown over time. As illustrated in Fig. 8h, i, the decay rate of the fluorescence intensity of Fam-siRNA and eGFP was not high during the initial stage (5–10 min) of nanoparticle internalization. Then, the residual siRNA and the gene expression product eGFP rapidly decreased with the extension of phagocytosis (10–30 min). Afterward, the decay rate of fluorescence leveled off again until 60 min. Interestingly, the dynamics showed a high degree of consistency with the participation timeline of different organelles in the intracellular delivery of nanoparticles (Fig. 7i). This revealed that the involvement of different organelles endowed the loaded gene medicines with different degradation characteristics. As shown in Fig. 8j, both the ER and EEs rapidly interacted and fused with the nanoparticle-containing phagosomes in the initial stage of phagocytosis, but they had a limited effect on the stability of the gene medicines. In contrast, the engagement of LEs and lysosomes significantly induced the degradation of the loaded siRNA and plasmid DNA (10–30 min). This was mainly derived from the involvement of many enzymes, including hydrolase, nuclease and protease. Notably, during the late stage of intracellular delivery, the Golgi apparatus and ER gradually dominated the interaction with phagosomes (30–60 min), which delayed the rapid degradation of gene medicines and promoted the slow release of drugs from phagosomes to the cytoplasm.

This finding demonstrated that the involvement of distinct organelles differentially affected the intracellular delivery efficiency of drug-loaded nanoparticles. More importantly, it provided a more accurate time window for the design of nanomaterials with lysosomal and endosomal escape capacity for gene medicine delivery. According to the dynamics shown in Fig. 8j, inducing escape at the initial or late stage of intracellular delivery might be a better choice because it avoids the influence of degrading enzymes in LEs and lysosomes.

## Conclusion

In this study, we developed an enzyme-induced proximity labeling technology in nanoparticles (nano-EPL) for the real-time monitoring of proteins that interact with intracellular nanomedicines. PLGA nanoparticles coupled with HRP were prepared as the nanomedicine model (HRP(+)-PNPs), and the J774A.1 cell line was used to evaluate the molecular mechanism of nano delivery in macrophages. By adding the labeling probe BP and the catalytic substrate H_2_O_2_ at different time points of nanoparticle internalization, proteins interacting with nanoparticles could be tagged with biotin in real time and in situ. After isolation, purification, identification and screening, 740 proteins constituted the dynamic molecular profile of the delivery of HRP(+)-PNPs in macrophages. These proteins interacted with nanoparticles at different times and affected the nano delivery in the clustering pattern according to their own location and function. Based on a series of dynamic clustering analyses of the molecular profile, we discovered that different organelles, including endosomes, lysosomes, the ER, and the Golgi apparatus, had independent participation timelines at the minute level during the intracellular delivery of nanoparticles in macrophages. ER and EEs took the lead in nano delivery (0–5 min). What followed was the involvement of LEs and lysosomes (5–30 min). The Golgi apparatus and ER were engaged during the late stage of intracellular delivery of nanoparticles (20–60 min). More importantly, the engagement of these organelles differentially affected the intracellular delivery efficiency of the loaded gene medicines. This study provided a detailed spatial–temporal mechanism for the design of efficient nanomedicines. In summary, the established nano-EPL technology achieved dynamic mapping of the intracellular delivery of nanomedicines at the molecular level. We believe that this technology can be expanded to more types of nanomedicines and cells to reveal the molecular mechanism of cellular delivery.

## Supplementary Information

Below is the link to the electronic supplementary material.

Supplementary file 1 (DOCX 6542 KB)

Supplementary file 2 (PDF 2802 KB)

Supplementary file 3 (PDF 1087 KB)

Supplementary file 4 (PDF 400 KB)
