# Supplementary material for: Molecular Mechanisms of Intracellular Delivery of Nanoparticles Monitored by an Enzyme-Induced Proximity Labeling
Source: Nanomicro Lett. 2024 Feb 1;16:103. doi: 10.1007/s40820-023-01313-0 (PMC10834923; doi:10.1007/s40820-023-01313-0)
Supplement: Supplementary file 1 — (DOCX 6542 KB) [file 40820_2023_1313_MOESM1_ESM.docx]

Supporting Information for

**Molecular mechanisms of intracellular delivery of nanoparticles monitored by an enzyme-induced proximity labeling**

Junji Ren, Zibin Zhang, Shuo Geng, Yuxi Cheng, Huize Han, Zhipu Fan, Wenbing Dai, Hua Zhang, Xueqing Wang, Qiang Zhang*, and Bing He*

Department of Pharmaceutics School of Pharmaceutical Sciences, Peking University, 38 Xueyuan Rd, Haidian District, Beijing 100191, People’s Republic of China

*Corresponding authors. E-mail: [zqdodo@bjmu.edu.cn](mailto:zqdodo@bjmu.edu.cn); hebingmumu@bjmu.edu.cn

#
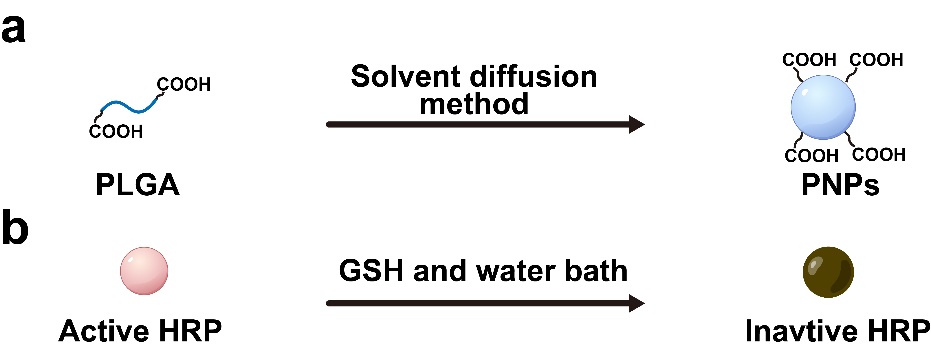
Supplementary Figures

##
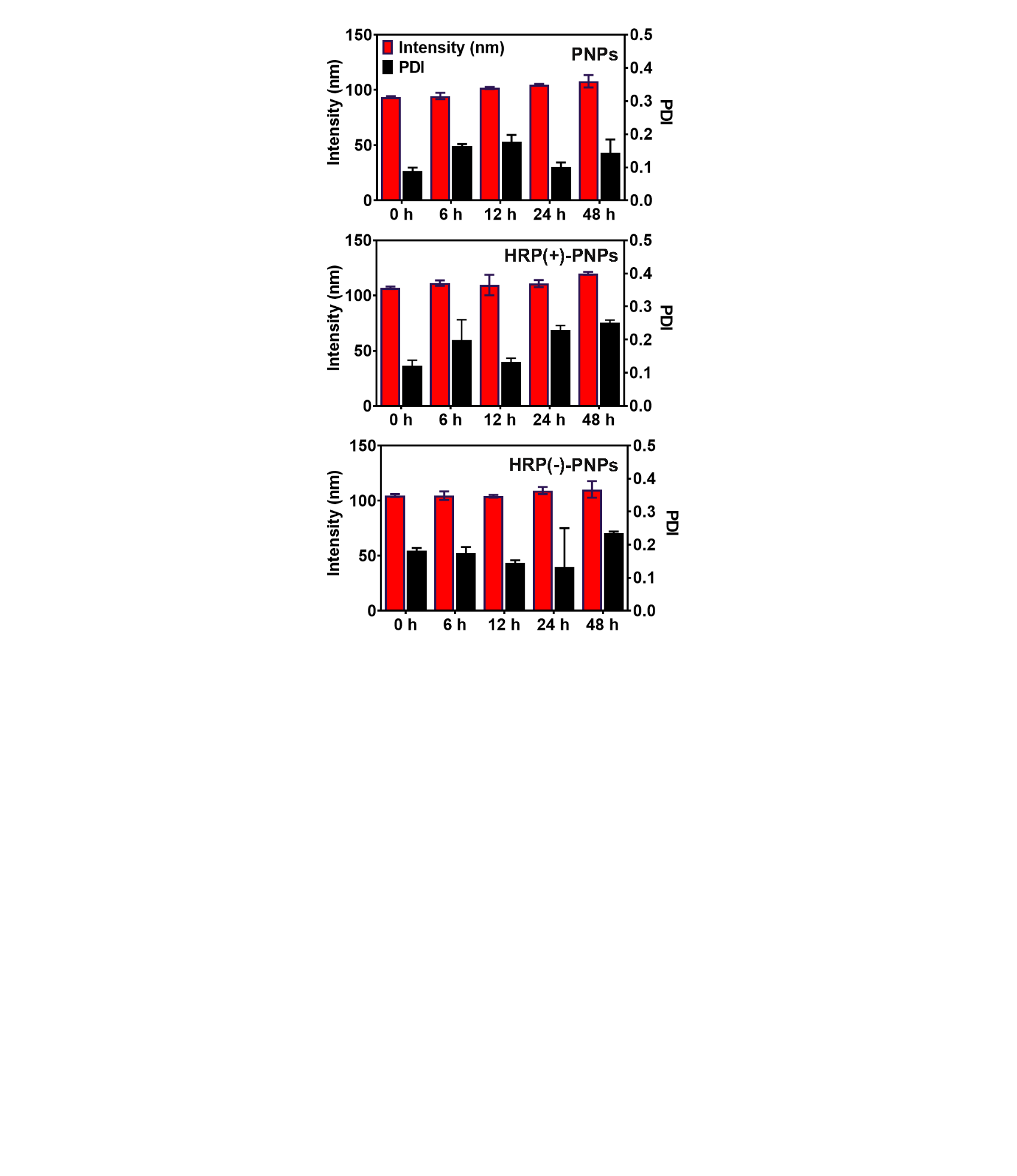
**Fig. S1** Schematic illustration of (**a**) PNPs and (**b**) HRP(-) preparation step

## **Fig. S2** Stability of PNPs and HRP(±)-PNPs over 48 h (n=3)

##
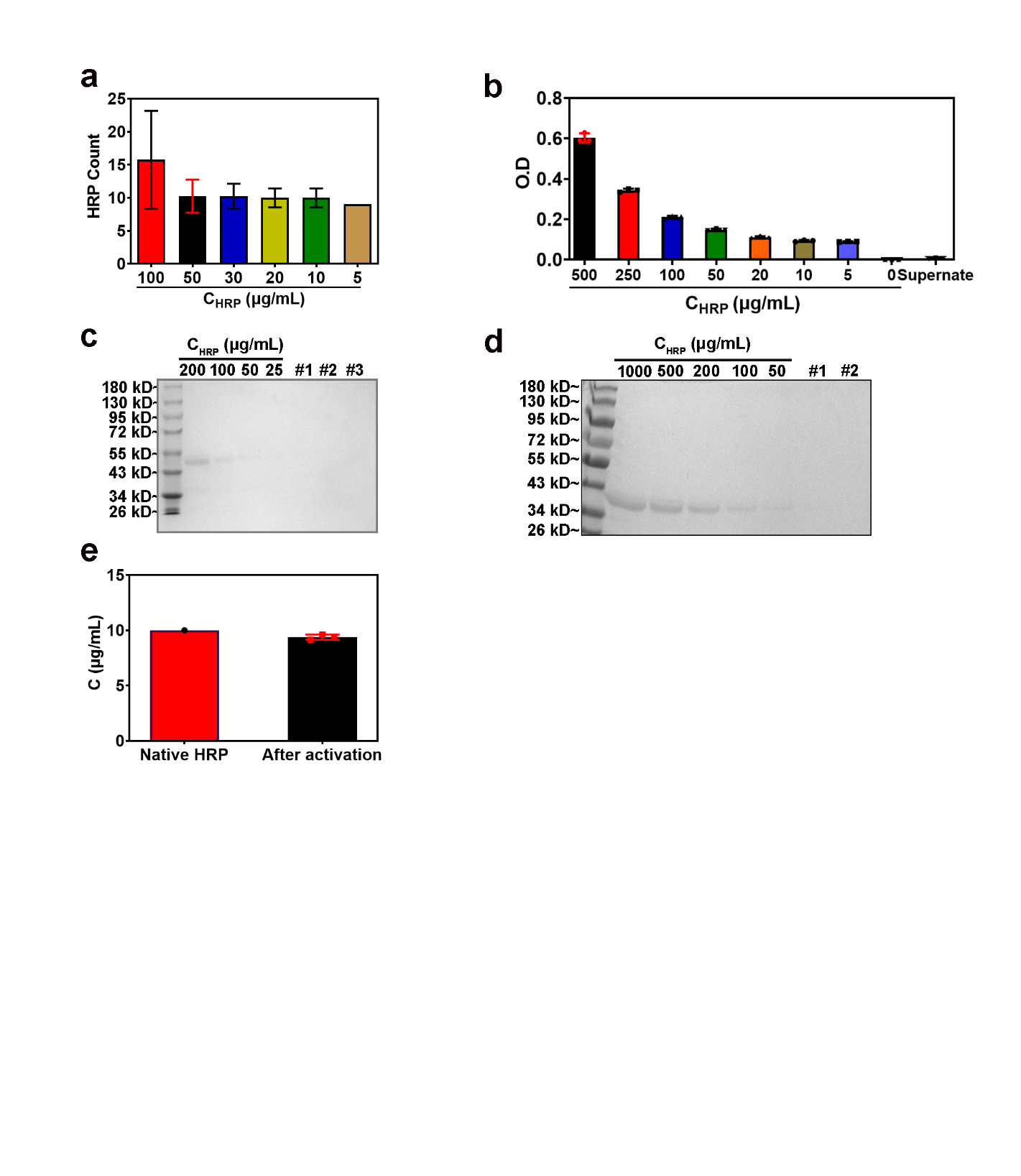
**Fig. S3** (**a**) The amount of active enzyme on HRP(+)-PNPs (n=3). (**b**) BCA analysis and (**c**) Coomassie staining of free HRP in the supernatant of HRP(+)-PNPs (n=3). (**d**) Coomassie staining of coupled HRP in the supernatant. # represents replicate sample. (**e**) Enzyme activity of HRP after NHS/EDC incubation for 2 h (n=3)

##
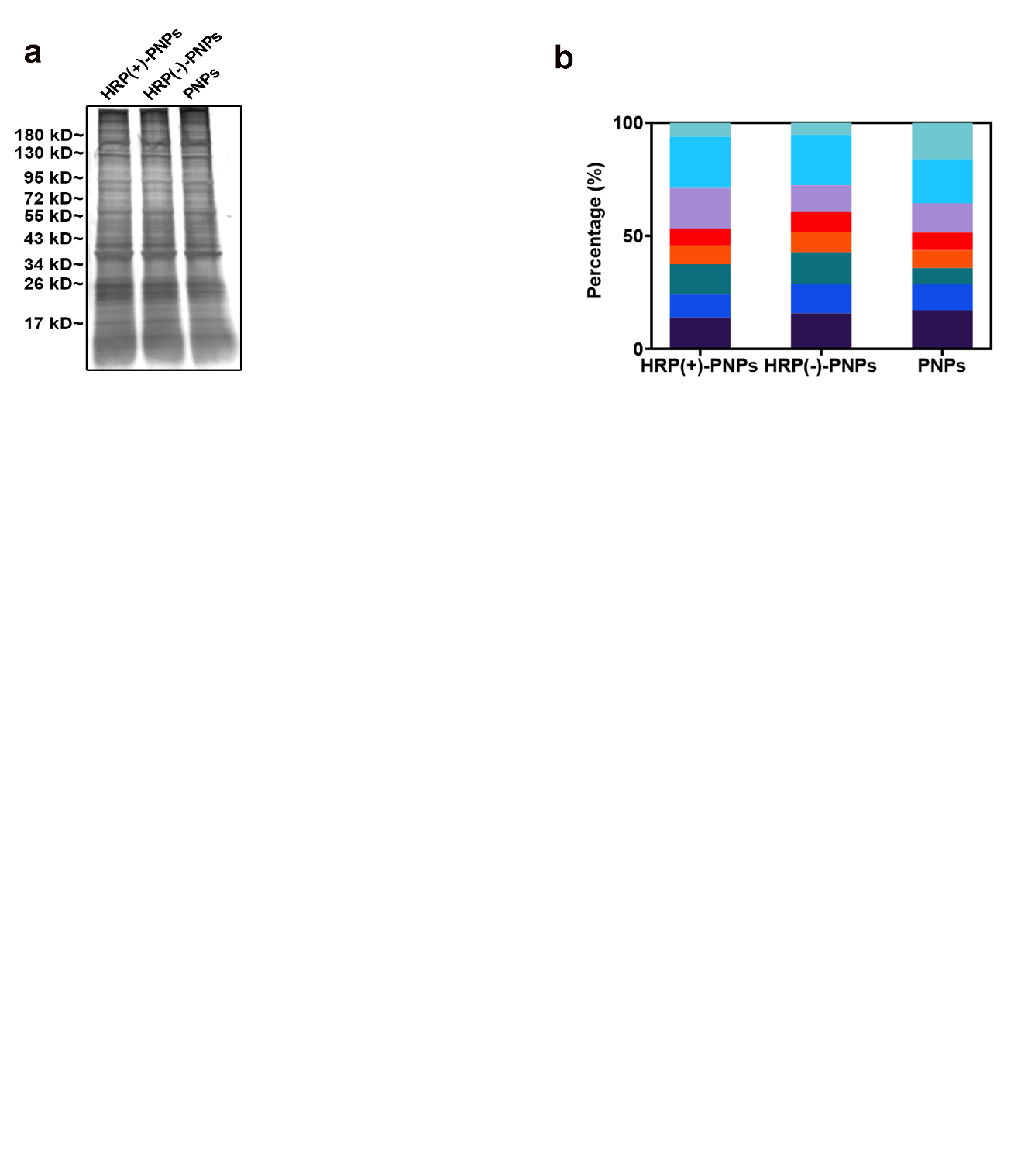
**Fig. S4** (**a**) Silver staining and (**b**) grayscale analysis of the protein corona on PNPs and HRP(±)-PNPs


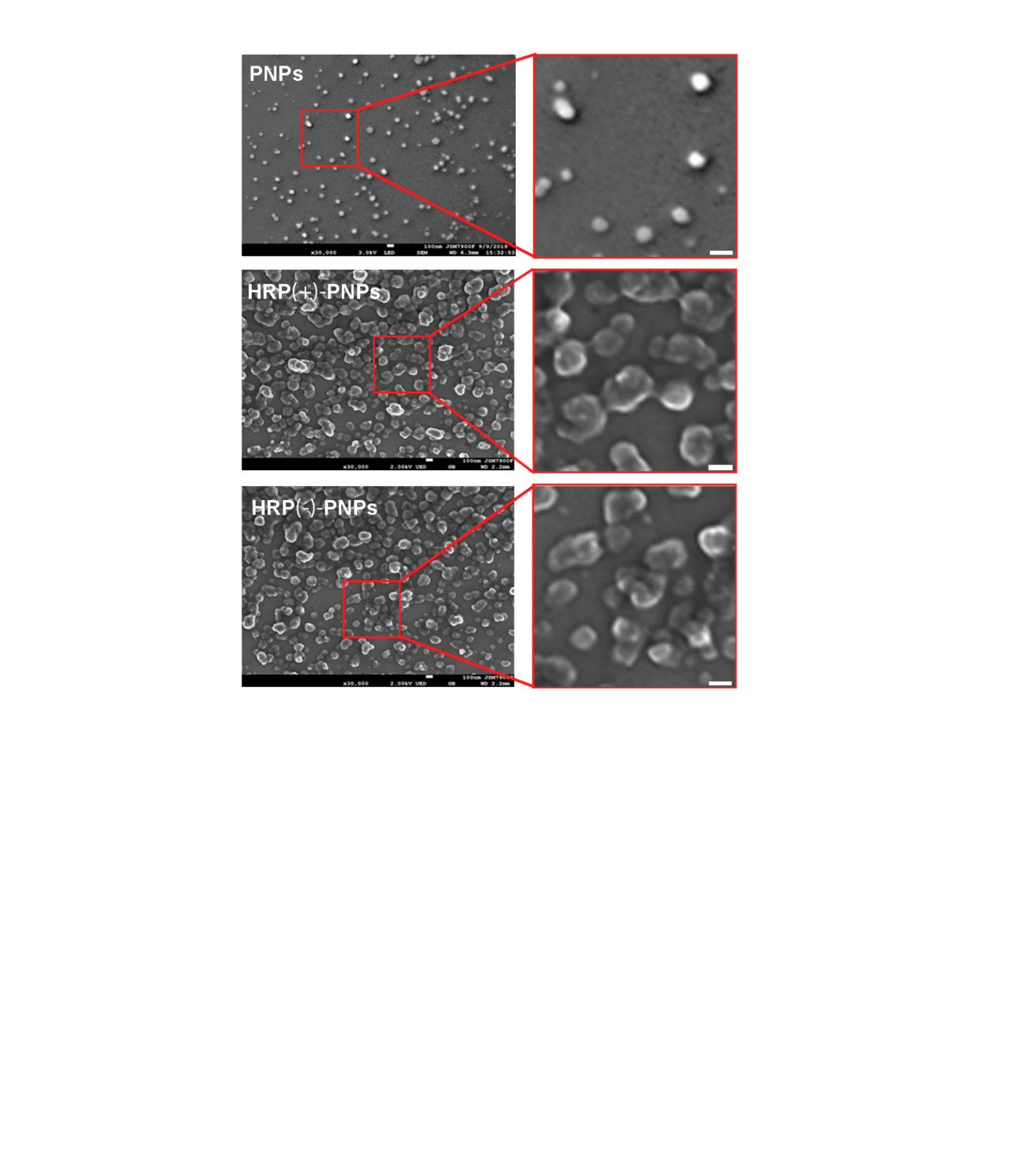


## **Fig. S5** SEM imaging of PNPs and HRP(±)-PNPs (scale bar, 0.1 μm)

##
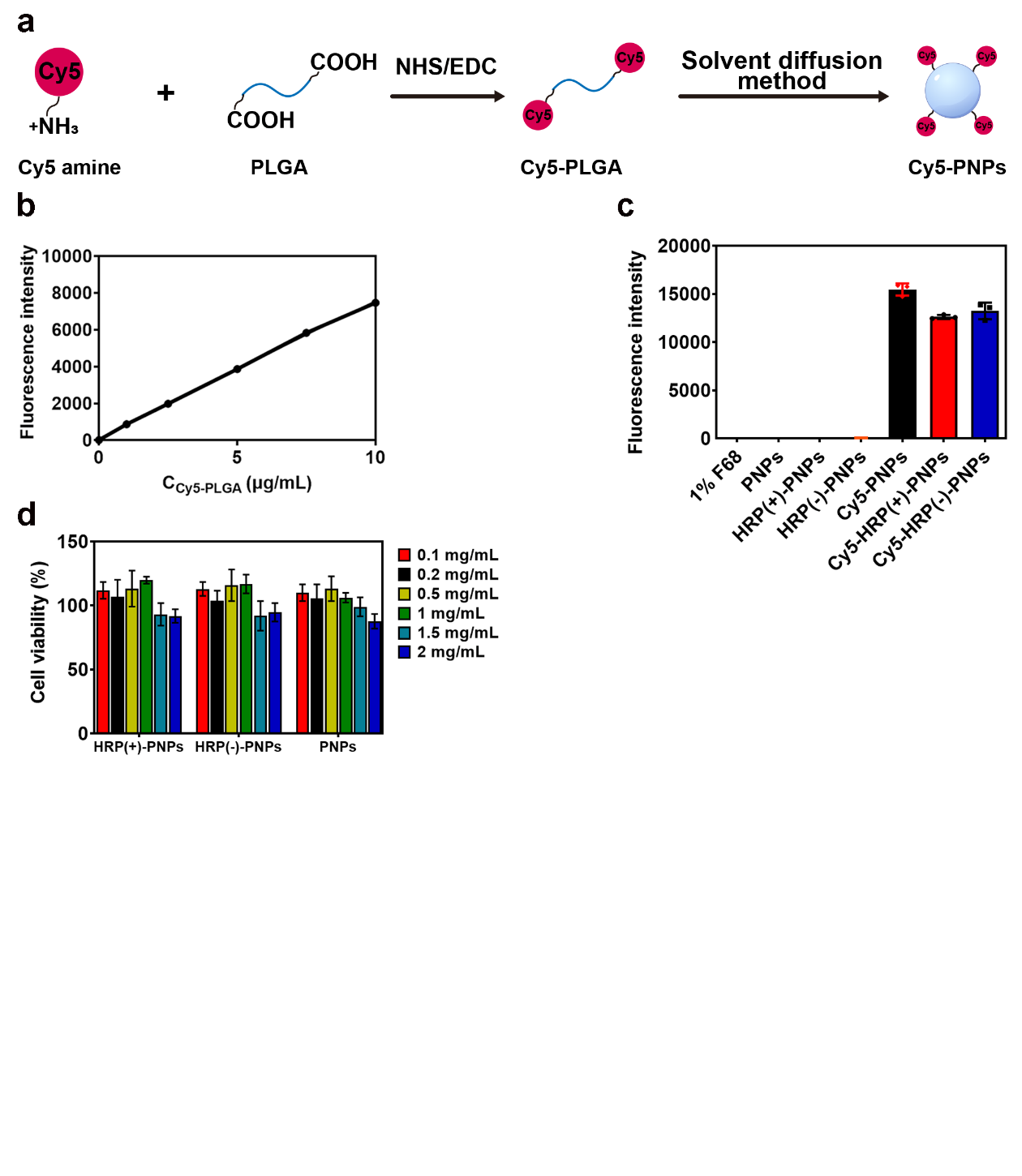
**Fig. S6** (**a**) Schematic illustration of Cy5-PNPs preparation. (**b**) Standard curves of the fluorescence intensity of Cy5-PLGA at 646 nm. (**c**) Mean fluorescence intensity of Cy5-PNPs, Cy5-HRP(+)-PNPs, Cy5-HRP(-)-PNPs. (**d**) Cell cytotoxicity assay of PNPs and HRP(±)-PNPs in J774A.1 cells evaluated by MTT assay

##
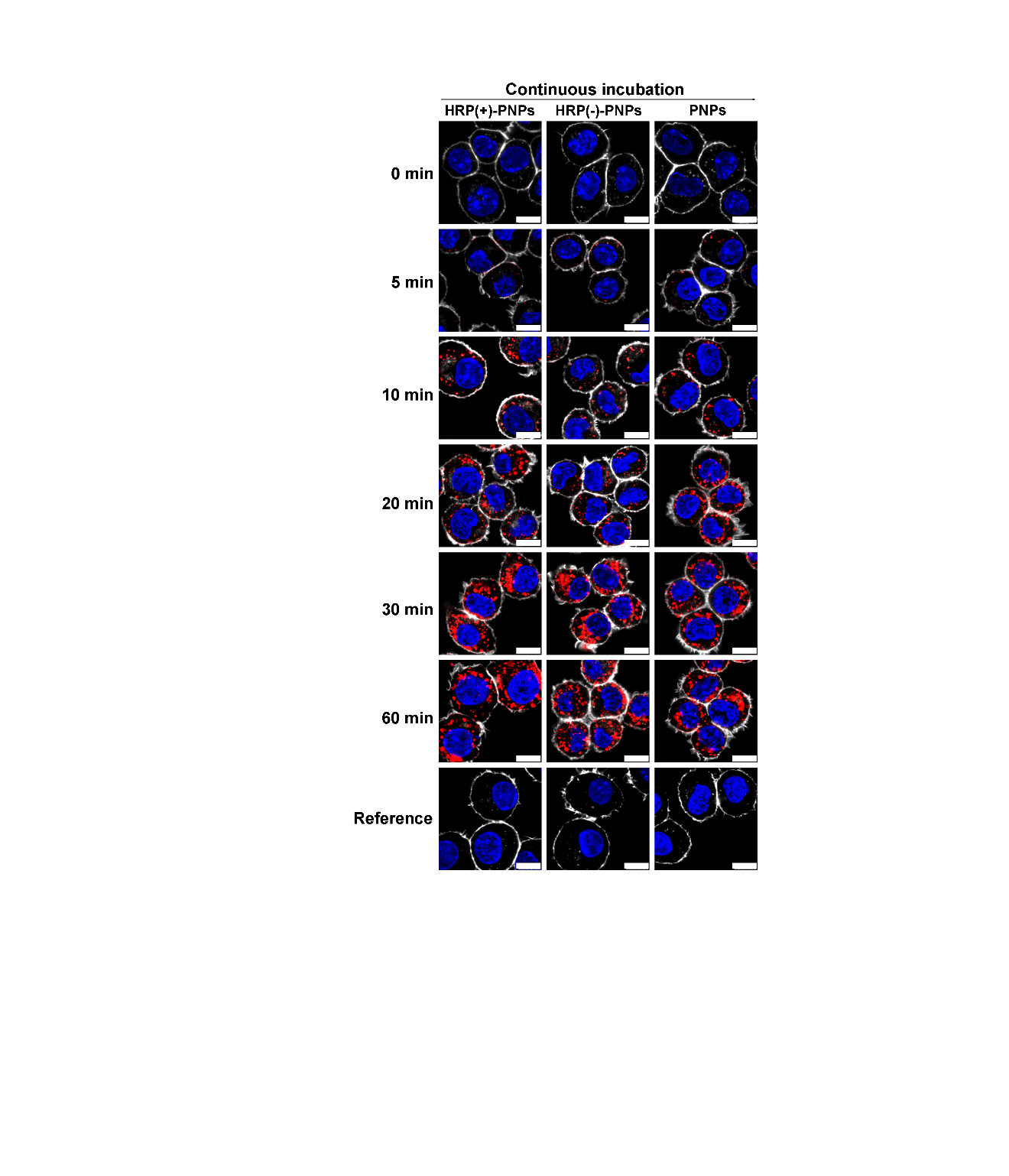
**Fig. S7** Fluorescence images of nanoparticles in J774A.1 cells with a continuous incubation over time (scale bar, 10 μm). Nanoparticles (red), nuclei (blue), cytomembrane (white)

##
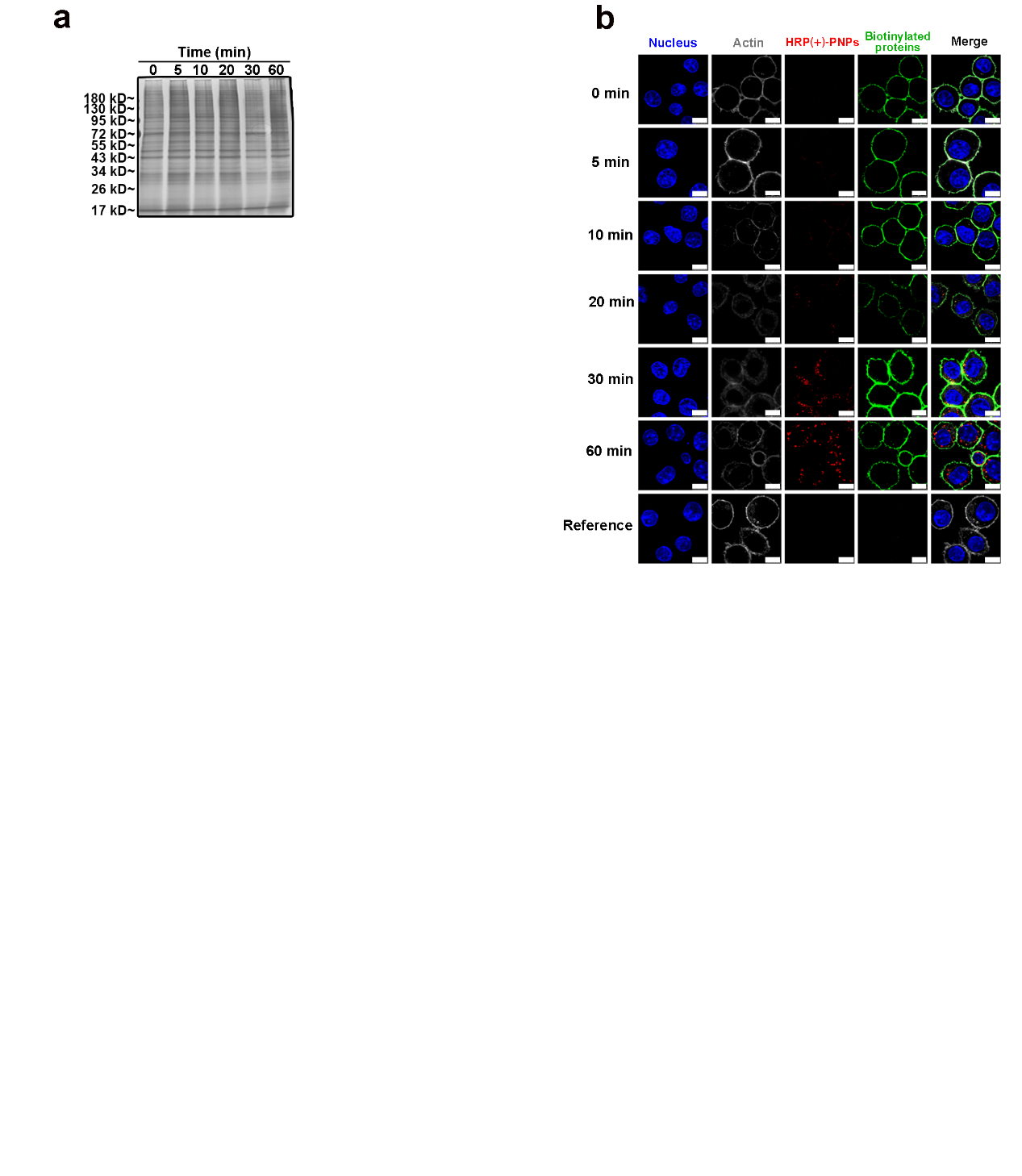
**Fig. S8** (**a**) Silver staining after streptavidin-beads enrichment of intracellular proteins labeled by HRP (+)-PNPs through a pulse-chase approach over time. (**b)** Fluorescence images of HRP (+)-PNPs in J774A.1 cells with a continuous incubation over time (scale bar, 10 μm)


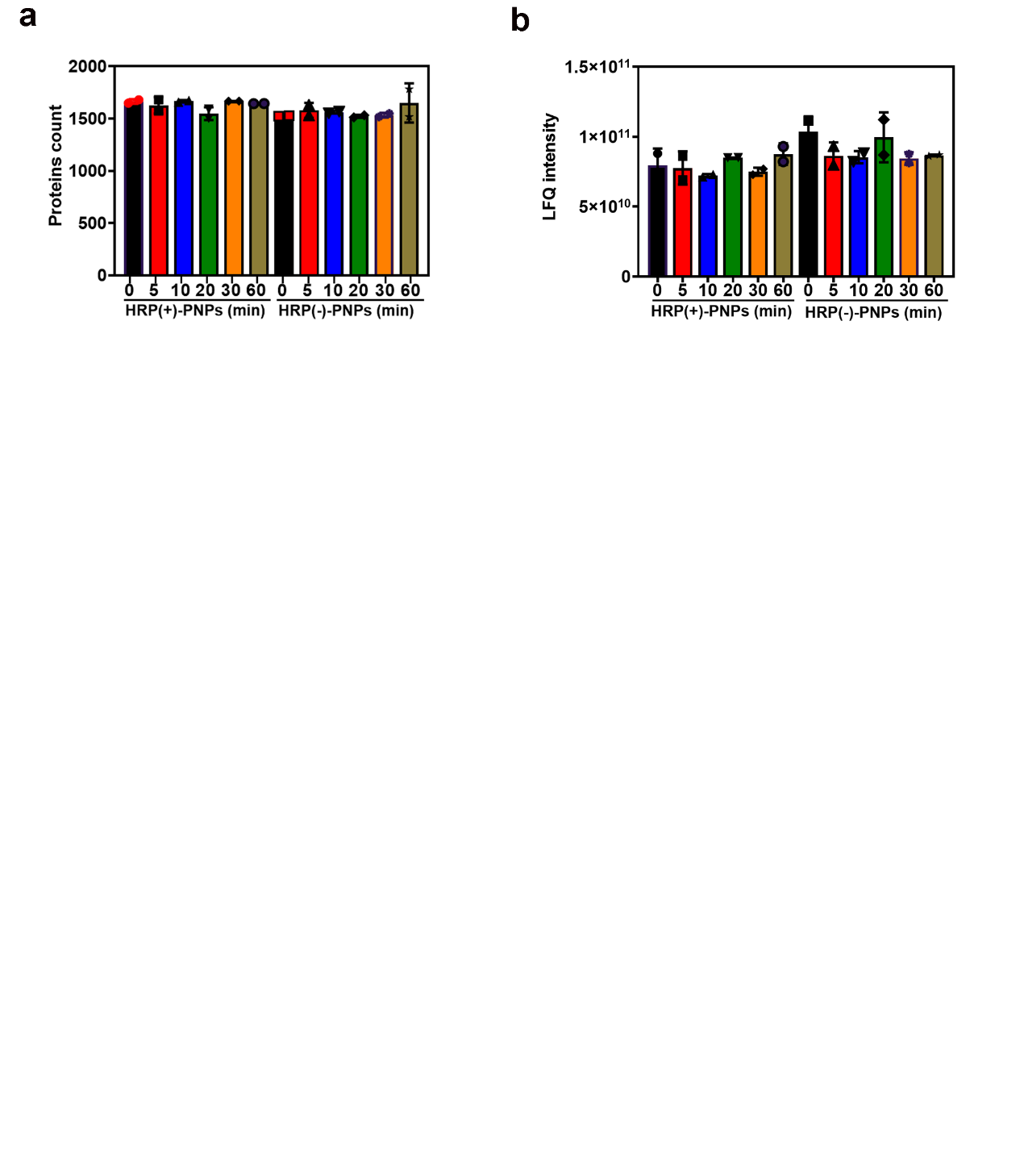


## **Fig. S9** (**a**) Count and (**b**) LFQ intensity of enriched total proteins at 6 time points in 12 groups


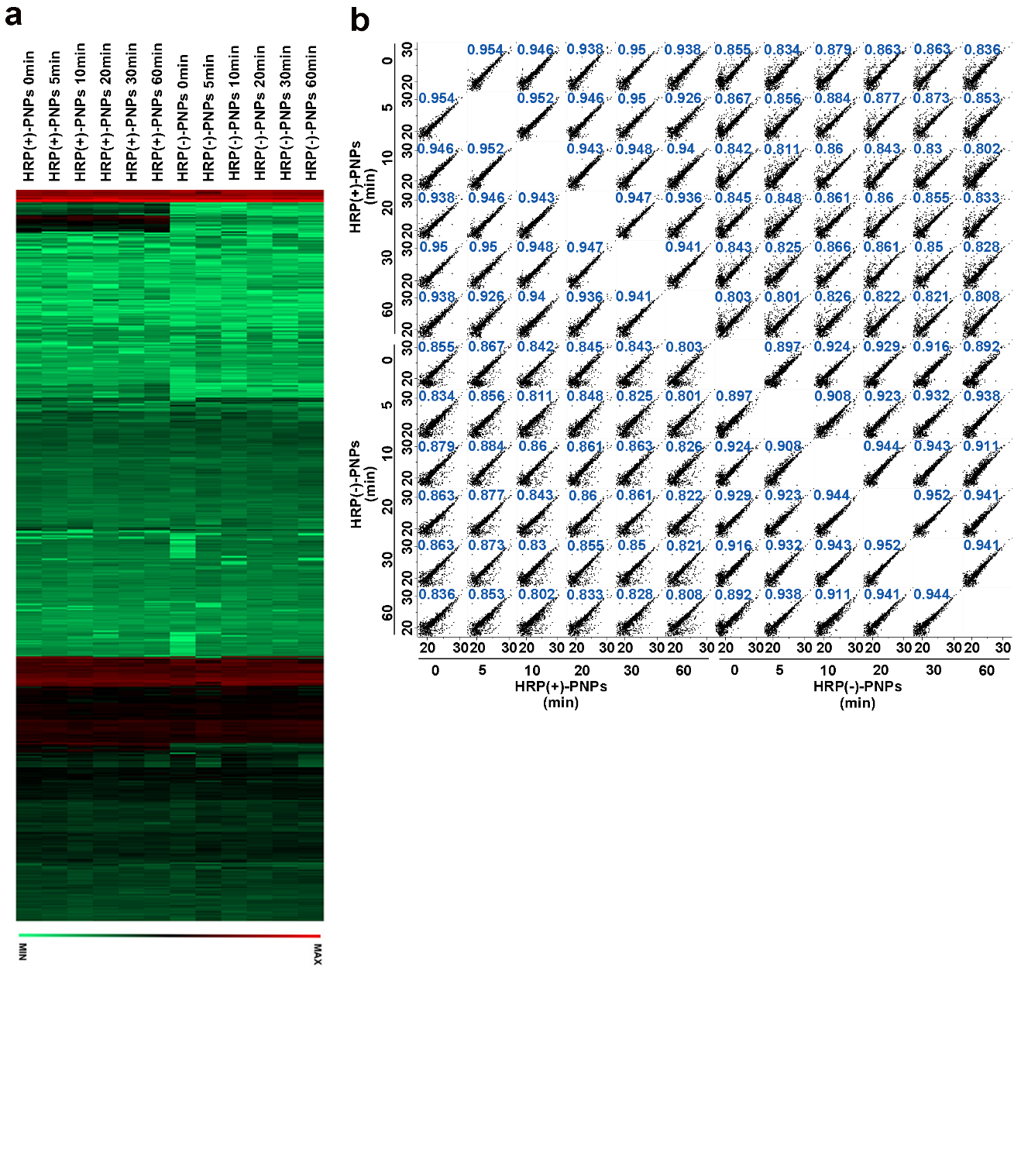


## **Fig. S10** (**a**) Heatmap and (**b**) scatter plot of the Pearson correlation coefficient of enriched total proteins in 12 groups


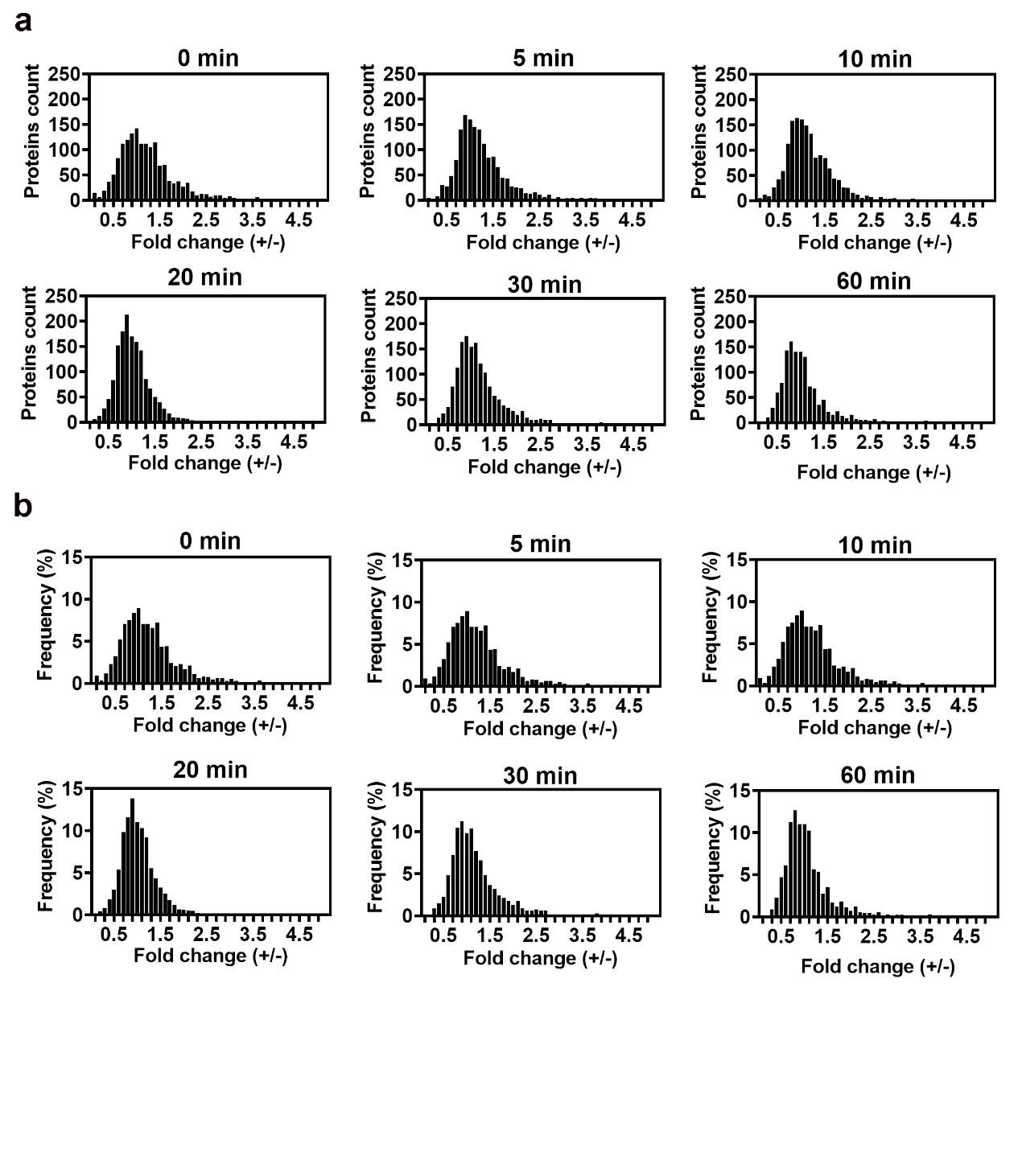


## **Fig. S11** (**a**) Proteins count and (**b**) frequency distribution histogram of protein abundance at 6 time points

##
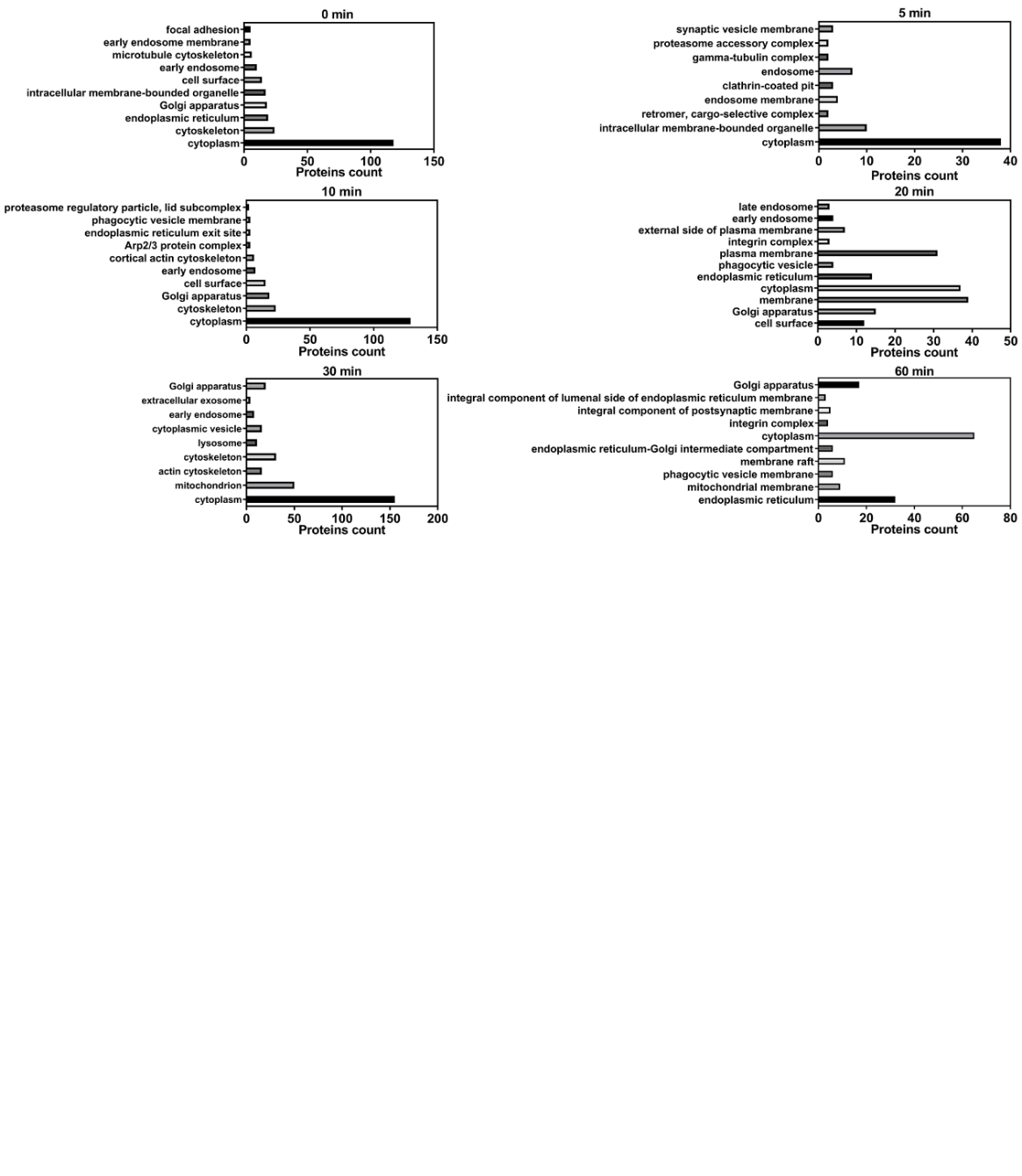
**Fig. S12** Diagram illustrating the GO analysis of the true-positive proteins at 6 time points

##
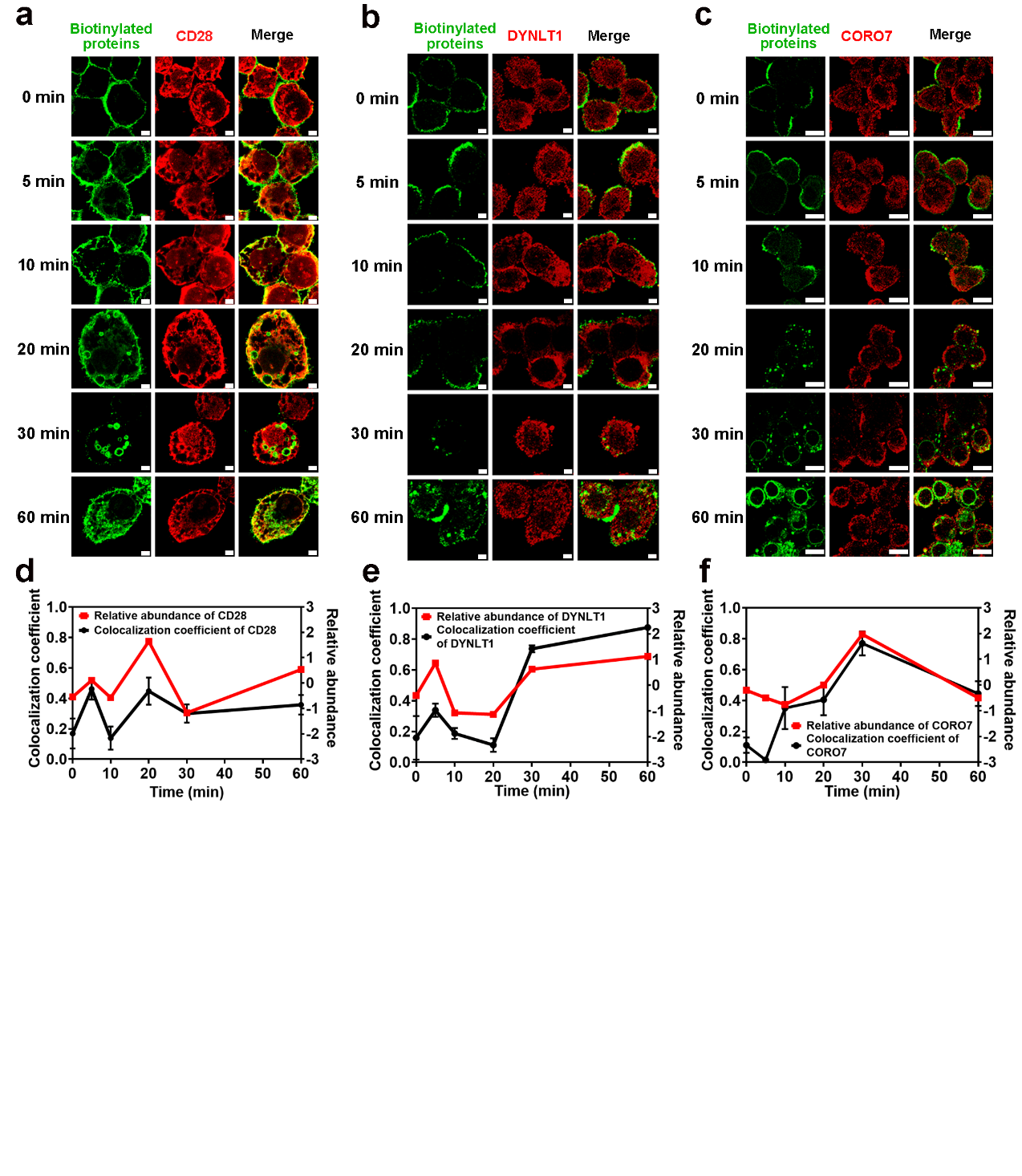
**Fig. S13** Fluorescence imaging of (**a**) CD28, (**b**) DYNLT1, (**c**) CORO7 and biotinylated proteins (scale bar, 2 μm). (**d**) Trend of relative abundance and colocalization assessed by regions between CD28, (**e**) DYNLT1, (**f**) CORO7 and biotinylated proteins over time (n=50)


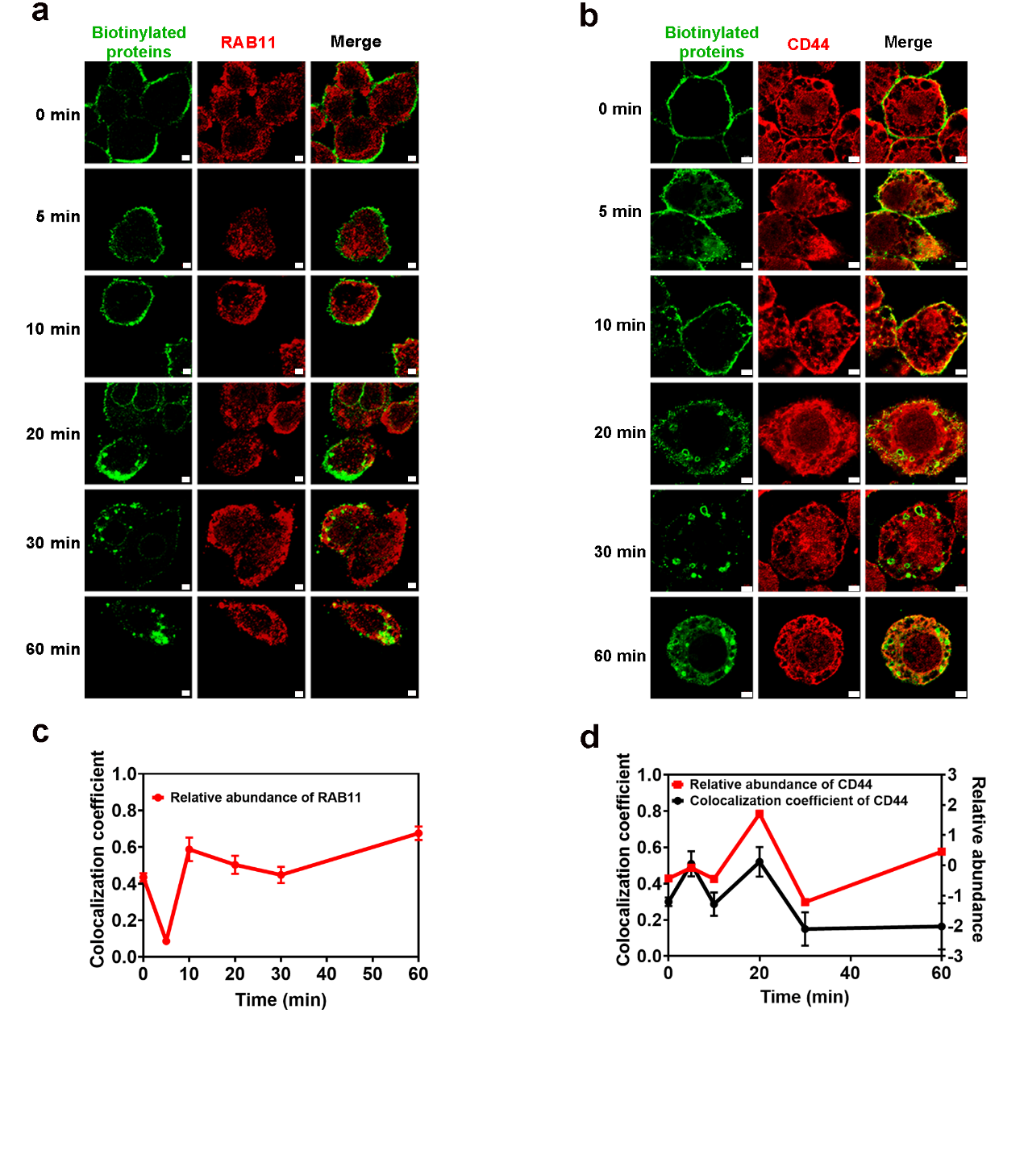


## **Fig. S14** (**a**) Fluorescence imaging of RAB11, (**b**) CD44 and biotinylated proteins (scale bar, 2 μm). (**c**) Trend of colocalization assessed by regions of RAB11 and biotinylated proteins over time. (**d**) Trend of colocalization assessed by regions and relative abundance between CD44 and biotinylated proteins over time (n=50)


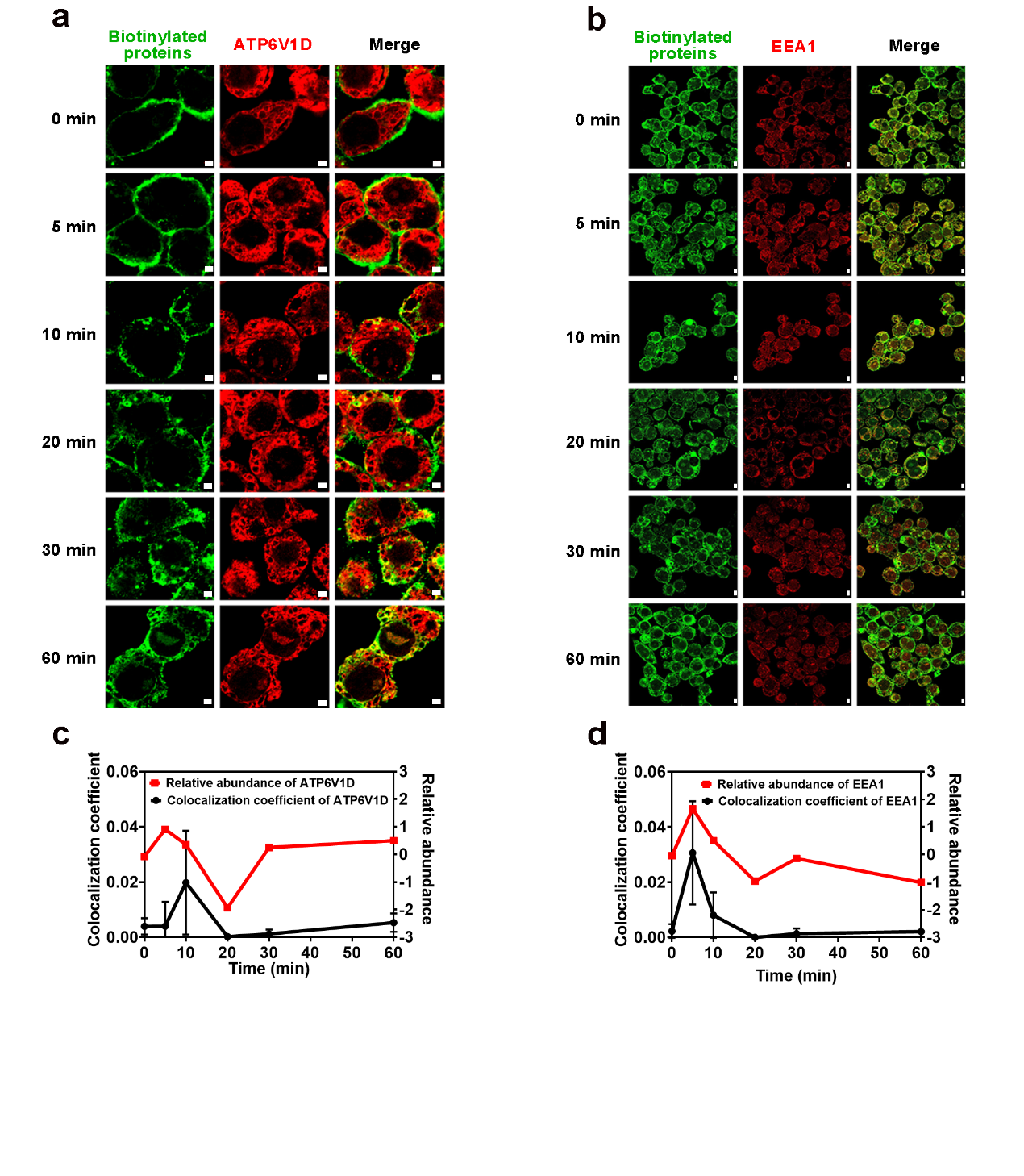


## **Fig. S15** (**a**) Fluorescence imaging of ATP6V1D, (**b**) EEA1 and biotinylated proteins (scale bar, 2 μm). Trend of colocalization assessed by regions and relative abundance between (**c**) ATP6V1D, (**d**) EEA1 and biotinylated proteins over time (n=50)

**Fig. S16** Phototoxicities of Ce6@PPNs in J774A.1 cells upon illumination at 660 nm (200 mW/cm^2^) evaluated by CCK-8 assay (n=3)
