# Supplementary material for: Molecular Mechanisms of Intracellular Delivery of Nanoparticles Monitored by an Enzyme-Induced Proximity Labeling
Source: Nanomicro Lett. 2024 Feb 1;16:103. doi: 10.1007/s40820-023-01313-0 (PMC10834923; doi:10.1007/s40820-023-01313-0)
Supplement: Supplementary file 2 — (PDF 2802 KB) [file 40820_2023_1313_MOESM2_ESM.pdf]

Table S1 Original proteomics data of 12 groups

[illegible]

[illegible]	36247000	23	F6XAZ3;Q07417	F6XAZ3;Q07417	[illegible]	Acads
0	5470500	13	[illegible]	[illegible]	FCH domain only protein 2	Fcho2
[illegible]	377180	2	[illegible]	[illegible]	Protein scribble homolog	Scrib
[illegible]	983540	2	F6RK81;Q9CQE7;Q9CQE7-2	F6RK81;Q9CQE7;Q9CQE7-2	[illegible]	Ergic3
0	21964000	7	F6RPJ9;Q9JHR7	F6RPJ9;Q9JHR7	Insulin-degrading enzyme	Ide
[illegible]	15206000	11	F6RSH1;F8WHM5;Q61543	F6RSH1;F8WHM5;Q61543	Golgi apparatus protein 1	Glg1
[illegible]	18893000	13	Q91ZP6-2;Q91ZP6;F6RT43;M0QWK1	Q91ZP6-2;Q91ZP6;F6RT43;M0QWK1	NEDD4 family-interacting protein 2	Ndfip2
[illegible]	25486000	6	F6SAC3	F6SAC3	Glucose-6-phosphate isomerase	Gm1840
[illegible]	4795900	10	[illegible]	[illegible]	[illegible]	Braf;Araf;Raf1
[illegible]	8031700	3	F6T2H5;Q6NSR8	F6T2H5;Q6NSR8	Probable aminopeptidase NPEPL1	Npepl1
1	0	0	F6WN43;F6UL52	F6WN43;F6UL52		Hscb
0	73622000	43	F6V084;Q8VBT0	F6V084;Q8VBT0	Thioredoxin-related transmembrane protein 1	Tmx1
[illegible]	18117000	3	F6V6T4;Q9R0Q3;Q8BPI2	F6V6T4;Q9R0Q3;Q8BPI2	Transmembrane emp24 domain-containing protein 2	Tmed2
[illegible]	30350000	9	G5E919;F7CDT0;F6WMC0;P61082	G5E919;F7CDT0;F6WMC0;P61082	NEDD8-conjugating enzyme Ubc12	Ube2m
0	377650000	111	O70370;F6WR04	O70370;F6WR04	Cathepsin S	Ctss
[illegible]	8723800	7	F6XGN0;Q9QY81	F6XGN0;Q9QY81	Nuclear pore membrane glycoprotein 210	Nup210
0	5427200	4	F6XQZ4;Q8BWZ3-2;Q8BWZ3	F6XQZ4;Q8BWZ3-2;Q8BWZ3	N-alpha-acetyltransferase 25, NatB auxiliary subunit	Naa25
[illegible]	11761000	1	[illegible]	F6YB25;Q9CSU0-3;Q9CSU0-2;Q9CSU0;F7DAY5	[illegible]	Rprd1b
[illegible]	2748300	5	F6YBV1;Q8K0V4	F6YBV1;Q8K0V4	CCR4-NOT transcription complex subunit 3	Cnot3
0	1628000000	355	P62270;F6YVP7;S4R1N6	P62270;F6YVP7;S4R1N6	40S ribosomal protein S18	Rps18;Gm10260
0	8570000	15	F6YY88;Q9CQT1;F8WI95	F6YY88;Q9CQT1;F8WI95	Methylthioribose-1-phosphate isomerase	Mri1
0	7274700	3	[illegible]	[illegible]	Misshapen-like kinase 1	Mink1
0	11573000	1	F7B227;O35459	F7B227;O35459	[illegible]	Ech1
0	1447900	2	F7B2B4;Q9D1K2	F7B2B4;Q9D1K2	V-type proton ATPase subunit F	Atp6v1f
0	32421000	23	F7B5B5;Q8VHM5;A2AW41;S4R1E9	F7B5B5;Q8VHM5;A2AW41		Hnrnpr
[illegible]	2986500	5	F7BUQ6;Q8CD26	F7BUQ6;Q8CD26	Solute carrier family 35 member E1	Slc35e1
0	9444300	1	F7BX26;Q60676	F7BX26;Q60676	[illegible]	Ppp5c
0	48219000	25	F7CHQ7;Q9D6Z1	F7CHQ7;Q9D6Z1	Nucleolar protein 56	Nop56
[illegible]	5202300	3	F7CXS7;Q9CQN7	F7CXS7;Q9CQN7	39S ribosomal protein L41, mitochondrial	Gm6434;Mrpl41
0	2151200	4	[illegible]	Q8JZR2;Q64010-2;F7D232;Q5NDS0;Q64010	Adapter molecule crk	Crk
0	20567000	13	[illegible]	Q6A4J8-3;Q6A4J8;F8VPX1;Q6A4J8-2;E9PXY8	[illegible]	Usp7
[illegible]	4296300	3	F8VPZ3	F8VPZ3	Ubiquitin carboxyl-terminal hydrolase	Usp32
0	682830	1	Q8VI36-2;Q8VI36;F8VQ28	Q8VI36-2;Q8VI36;F8VQ28	Paxillin	Pxn
[illegible]	6162500	6	F8VQ29;Q3UQP1;A0A0A6YXJ2	F8VQ29;Q3UQP1		Iqgap3
0	3304500	4	F8VQ65;O08632	F8VQ65;O08632	Keratin-associated protein 19-5	Krtap19-5
0	20723000	14	Q8BTY2-2;Q8BTY2;F8VQC9	Q8BTY2-2;Q8BTY2;F8VQC9	Sodium bicarbonate cotransporter 3	Slc4a7
1	0	0	F8VQM5	F8VQM5		Sis
0	90446000	39	F8WGG3;Q9JK81;F7A3N3	F8WGG3;Q9JK81;F7A3N3	UPF0160 protein MYG1, mitochondrial	Myg1
0	73019000	48	Q64324;F8WGM5	Q64324;F8WGM5	Syntaxin-binding protein 2	Stxbp2
0	33572000	5	[illegible]	[illegible]	[illegible]	Ahcyl2;Ahcyl1
0	0	1	F8WGV3	F8WGV3		Slc26a10
0	51866000	41	S4R293;Q09014;F8WH69	S4R293;Q09014;F8WH69	Neutrophil cytosol factor 1	Ncf1
0	584740000	375	Q8CIE6;F8WHL2;F6XIN3	Q8CIE6;F8WHL2	Coatomer subunit alpha;Xenin;Proxenin	Copa
0	35834000	40	Q569Z5-2;F8WHR6;Q569Z5	Q569Z5-2;F8WHR6;Q569Z5	Probable ATP-dependent RNA helicase DDX46	Ddx46
0	189480000	4	[illegible]	Q4VA53-2;Q4VA53-3;F8WHU5;Q4VA53	Sister chromatid cohesion protein PDS5 homolog B	Pds5b
0	4572500	8	F8WHU8;Q9D8T7;Q9D8T7-2	F8WHU8;Q9D8T7;Q9D8T7-2	[illegible]	Slirp
0	2050600	7	Q6P8I4;F8WI88;D3YWA4;Q3TMB9	Q6P8I4;F8WI88;D3YWA4;Q3TMB9	PEST proteolytic signal-containing nuclear protein	Pcnp
0	46780000	10	P10923;F8WIP8	P10923;F8WIP8	Osteopontin	Spp1
0	82100000	29	F8WIT2;P14824	F8WIT2;P14824	Annexin;Annexin A6	Anxa6
0	249560000	116	[illegible]	[illegible]	Dynamin-2	Dnm2
0	53409000	23	[illegible]	F8WIX8;Q64523;Q6GSS7	[illegible]	Hist1h2al;Hist2h2ac;Hist2h2aa1
[illegible]	2008700	2	Q61702;F8WJ05	Q61702;F8WJ05	Inter-alpha-trypsin inhibitor heavy chain H1	Itih1
[illegible]	244170	1	Q3UMY5-3;Q3UMY5;F8WJ93	Q3UMY5-3;Q3UMY5;F8WJ93	Echinoderm microtubule-associated protein-like 4	Eml4
0	13541000	4	Q9ESV0;F8WJA0	Q9ESV0;F8WJA0	ATP-dependent RNA helicase DDX24	Ddx24
0	12089000	1	F8WJI3;Q8R3Q6	F8WJI3;Q8R3Q6	Coiled-coil domain-containing protein 58	Ccdc58
0	342220000	92	F8WJK8;Q99L47;E9Q1V0;E9Q1X9	F8WJK8;Q99L47;E9Q1V0	Hsc70-interacting protein	St13
0	631540000	214	P62897;G3UWG1;CON__P62894;P00015	P62897;G3UWG1	Cytochrome c, somatic	Cycs;Gm10108
[illegible]	6870600	4	[illegible]	[illegible]	28S ribosomal protein S29, mitochondrial	Dap3
0	5994300	24	[illegible]	[illegible]	[illegible]	Gigyf2
[illegible]	1261400	1	G3UWV6;O35083	G3UWV6;O35083	1-acyl-sn-glycerol-3-phosphate acyltransferase alpha	Agpat1
0	8386900	42	G3UZ82;G3UWZ0;O88379	G3UZ82;G3UWZ0;O88379	[illegible]	Baz1a
0	1285400000	169	G3UX26;Q60930;D3YZT5;D3YUN8	G3UX26;Q60930;D3YZT5	Voltage-dependent anion-selective channel protein 2	Vdac2
0	98616000	52	[illegible]	Q8JZL74;G3UXA6;Q8BHD7-2;Q8BHD7;G3UZ01	Polypyrimidine tract-binding protein 3	Ptbp3
[illegible]	31839000	17	[illegible]	[illegible]	Casein kinase II subunit beta	Csnk2b
0	117720000	58	Q9D7G0;Q9CS42;G3UXL2	Q9D7G0;Q9CS42;G3UXL2	[illegible]	Prps1;Prps2;Prps1l3
0	58157000	33	[illegible]	Q99LD4;Q3MIA8;G3UXW9;Q99LD4-2;B1ATU4	COP9 signalosome complex subunit 1	Gps1
0	66104000	17	G3UXX3;Q64105;Q91XH5;G3UZ79	G3UXX3;Q64105;Q91XH5	Sepiapterin reductase	Spr
0	273790000	84	[illegible]	G3UXZ5;P97371;G3X9K9;G3UXY0;G3UWN9	Proteasome activator complex subunit 1	Psme1
0	36117000	18	[illegible]	[illegible]	Polyadenylate-binding protein 2	Pabpn1;Gm20521
[illegible]	0	0	G3UYG3	G3UYG3		Fap
[illegible]	1090700	1	G3UYL4;Q3U1Z5-2;Q3U1Z5	G3UYL4;Q3U1Z5-2;Q3U1Z5	G-protein-signaling modulator 3	Gpsm3
[illegible]	53913000	7	[illegible]	Q8CFZ0;G3UYP0;P63280	SUMO-conjugating enzyme UBC9	Ube2i
0	140340000	54	G3UYV7;P62858;I3QNN8	G3UYV7;P62858	40S ribosomal protein S28	Rps28
0	166000000	55	G3X8R0;Q60870	G3X8R0		Reep5
0	4931600	3	Q3UA37;G3X8R5;A0A0A6YXZ6;Q8BUN3	Q3UA37;G3X8R5;A0A0A6YXZ6;Q8BUN3	Glutamine-rich protein 1	Qrich1
0	1372000	24	[illegible]	Q9CQV7;Q9CQV7-3;Q9CQV7-2;G3X851	[illegible]	Dnajc19
0	20477000	4	H3BJ39;Q0P678;H3BIW0;G3X8T2	H3BJ39;Q0P678;H3BIW0;G3X8T2	Zinc finger CCCH domain-containing protein 18	Zc3h18
0	691980000	249	P16675;G3X8T3;A2A5J9;A2A5J8	P16675;G3X8T3	[illegible]	Ctsa
0	31673000	27	Q920Q4;G3X8X7;Q920Q4-2;A2BI90	Q920Q4;G3X8X7;Q920Q4-2	[illegible]	Vps16
0	136500000	78	[illegible]	G3X8Y3;A0A0A6YW80;Q80UM3	N-alpha-acetyltransferase 15, NatA auxiliary subunit	Naa15
0	103760000	50	Q9QUN7;G3X8Y8	Q9QUN7;G3X8Y8	Toll-like receptor 2;Toll-like receptor	Tlr2
[illegible]	[illegible]	[illegible]	[illegible]	[illegible]	[illegible]	[illegible]

[illegible]	[illegible]	1	[illegible]	[illegible]	Ribosomal RNA processing protein 1 homolog A	Rrp1
[illegible]	18796000	10	P56391	P56391	Cytochrome c oxidase subunit 6B1	Cox6b1
[illegible]	21317000	16	P56394	P56394	Cytochrome c oxidase copper chaperone	Cox17
0	358090000	153	Q3U4W8;P56399;D3Z4K7;D3YYA5	Q3U4W8;P56399	[illegible]	Usp5
0	9441500000	1400	P56480	P56480	ATP synthase subunit beta, mitochondrial	Atp5b
0	10070000	11	[illegible]	[illegible]	[illegible]	Ctbp2;Ctbp1
[illegible]	30175000	8	P56677	P56677	Suppressor of tumorigenicity 14 protein homolog	St14
0	21398000	44	P56812;D3Z7Q5	P56812	Programmed cell death protein 5	Pdcd5
0	607500000	156	[illegible]	Q8CFQ9;P56959;G3UXT7	RNA-binding protein FUS	Fus
0	154860000	84	P57716	P57716	Nicastrin	Ncstn
0	45418000	7	P57746	P57746	V-type proton ATPase subunit D	Atp6v1d
0	660980000	207	P57759;F8WJI4;F8WIM7;D6RG87	P57759	Endoplasmic reticulum resident protein 29	Erp29
0	88646000	24	[illegible]	P57776-2;Q91VK2;F6ZRU0;D3Z7N2;D3YY68	Elongation factor 1-delta	Eef1d
0	295350000	115	[illegible]	P57780	Alpha-actinin-4	Actn4
0	63771000	29	P57784;G5E883	P57784	U2 small nuclear ribonucleoprotein A	Snrpa1
[illegible]	3048000	5	P58064	P58064	28S ribosomal protein S6, mitochondrial	Mrps6
0	21288000	5	P58242	P58242	Acid sphingomyelinase-like phosphodiesterase 3b	Smpdl3b
0	4071900000	1109	P58252;G3UXK8	P58252	Elongation factor 2	Eef2
0	27730000	11	Q9JKB1;P58321	Q9JKB1;P58321	[illegible]	Uchl3;Uchl4
0	18940000	20	[illegible]	P58389	Serine/threonine-protein phosphatase 2A activator	Ppp2r4
0	55225000	23	P58681	P58681	Toll-like receptor 7	Tlr7
0	25630000	20	P58742	P58742	Aladin	Aaas
[illegible]	1432100	0	P58854	P58854	Gamma-tubulin complex component 3	Tubgcp3
0	22493000	14	P59017	P59017	Bcl-2-like protein 13	Bcl2l13
0	621080000	123	P59999;E9PWA7	P59999;E9PWA7	Actin-related protein 2/3 complex subunit 4	Arpc4
[illegible]	8202500	8	P60060;G3UWH0	P60060;G3UWH0	Protein transport protein Sec61 subunit gamma	Sec61g
0	192400000	109	P60122;D3YW60	P60122;D3YW60	RuvB-like 1	Ruvbl1
0	414670000	138	P60229	P60229	Eukaryotic translation initiation factor 3 subunit E	Eif3e
0	789840000	245	P60335	P60335	Poly(rC)-binding protein 1	Pcbp1
0	25124000	19	P60670-2;P60670	P60670-2;P60670	Nuclear protein localization protein 4 homolog	Nploc4
0	649730000	102	[illegible]	P60710;E9Q1F2;E9Q5F4	[illegible]	Actb
0	444490000	144	[illegible]	P60766	Cell division control protein 42 homolog	Cdc42
0	3894700000	735	[illegible]	P60843	Eukaryotic initiation factor 4A-I	Eif4a1
0	770030000	143	P60867	P60867	40S ribosomal protein S20	Rps20
[illegible]	20682000	3	P60904	P60904	DnaJ homolog subfamily C member 5	Dnajc5
0	206700000	85	P61027;D3YU54	P61027	Ras-related protein Rab-10	Rab10
0	162080000	81	P61028	P61028	Ras-related protein Rab-8B	Rab8b
0	56284000	14	P61079;Q6ZWY6;P62838	P61079;Q6ZWY6;P62838	[illegible]	Ube2d3;Ube2d2b;Ube2d2
0	140680000	54	P61089	P61089	Ubiquitin-conjugating enzyme E2 N	Ube2n
0	613350000	198	P61161	P61161	Actin-related protein 2	Actr2
0	151800000	63	P61164	P61164	Alpha-centractin	Actr1a
0	43247000	22	P61202;A2AQE4;P61202-2	P61202;A2AQE4;P61202-2	COP9 signalosome complex subunit 2	Cops2
0	636240000	226	P84078;P61205;D3YV25;E9Q2C2	P84078;P61205	ADP-ribosylation factor 1;ADP-ribosylation factor 3	Arf1;Arf3
0	28919000	11	P61211;F8WIB1	P61211;F8WIB1	ADP-ribosylation factor-like protein 1	Arl1
0	170970000	66	P61222	P61222	ATP-binding cassette sub-family E member 1	Abce1
0	51435000	16	P61226;Q80ZJ1-2;Q8BU31;Q80ZJ1	P61226;Q80ZJ1-2;Q8BU31;Q80ZJ1	[illegible]	Rap2b;Rap2a;Rap2c
0	1546900000	350	P61255;B1ARA3;B1ARA5	P61255;B1ARA3;B1ARA5	60S ribosomal protein L26	Rpl26
0	77179000	53	P61290;A2A4J3;A2A4J1	P61290;A2A4J3;A2A4J1	Proteasome activator complex subunit 3	Psme3
0	1491900000	205	P61358;A2A4Q0	P61358;A2A4Q0	60S ribosomal protein L27	Rpl27
0	58737000	39	P61514	P61514	60S ribosomal protein L37a	Rpl37a
0	101400000	56	P61620;A2ATT9;Q9CYJ6;Q9JLR1	P61620	Protein transport protein Sec61 subunit alpha isoform 1	Sec61a1
[illegible]	659580	1	P61750;E9Q798;F6UF89	P61750	ADP-ribosylation factor 4	Arf4
0	22027000	9	P61759;Q3TIR6	P61759;Q3TIR6	Prefoldin subunit 3	Vbp1
0	98003000	36	P61804	P61804	[illegible]	Dad1
0	121570000	38	P61924	P61924	Coatomer subunit zeta-1	Copz1
0	24079000	15	P61961	P61961	Ubiquitin-fold modifier 1	Ufm1
[illegible]	33142000	34	P61965	P61965	WD repeat-containing protein 5	Wdr5
0	17691000	3	P61967	P61967	AP-1 complex subunit sigma-1A	Ap1s1
0	2083400000	467	[illegible]	[illegible]	Heterogeneous nuclear ribonucleoprotein K	Hnrnpk;Gm7964
0	976140000	215	P61982	P61982	[illegible]	Ywhag
0	23458000	10	P62075	P62075	[illegible]	Timm13
0	1767300000	397	P62082;F6SVV1	P62082;F6SVV1	40S ribosomal protein S7	Rps7;Gm9493
0	158210000	27	P62137	P62137	[illegible]	Ppp1ca
0	318400000	119	P62192	P62192	26S protease regulatory subunit 4	Psmc1
0	443920000	156	P62196;Q8K1K2	P62196;Q8K1K2	26S protease regulatory subunit 8	Psmc5
0	556810000	53	P62204;Q3UKW2;P20801	P62204;Q3UKW2	Calmodulin	Calm1

0	27521000	33	[illegible]	[illegible]		Sec24b
0.0046707	10523000	7	Q810A7-2;Q810A7	Q810A7-2;Q810A7	ATP-dependent RNA helicase DDX42	Ddx42
0	90029000	59	Q810B6;Q810B6-2	Q810B6	Ankyrin repeat and FYVE domain-containing protein 1	Ankfy1
0.0065994	14996000	4	Q810D6	Q810D6	Glutamate-rich WD repeat-containing protein 1	Grwd1
0	66957000	25	Q810U4-2;Q810U4;Q810U4-3	Q810U4-2;Q810U4;Q810U4-3	Neuronal cell adhesion molecule	Nrcam
0	9567000	3	Q8BFQ4	Q8BFQ4	WD repeat-containing protein 82	Wdr82
0	145990000	103	Q8BFR4	Q8BFR4	N-acetylglucosamine-6-sulfatase	Gns
0.0025818	256920	1	Q8BFV2	Q8BFV2	PCI domain-containing protein 2	Pcid2
0.0047642	2557400000	11	Q8BFZ3;E9Q3M9	Q8BFZ3	Beta-actin-like protein 2	Actbl2
0	56192000	76	Q8BFZ9	Q8BFZ9	Erlin-2	Erlin2
0	475530000	151	Q8BG07-2;Q8BG07	Q8BG07-2;Q8BG07	Phospholipase D4	Pld4
0	1050700	3	Q8BG30	Q8BG30	Negative elongation factor A	Nelfa
0	345100000	146	[illegible]	Q8BG32	26S proteasome non-ATPase regulatory subunit 11	Psmd11
0.005223	4849000	3	Q8BG36	Q8BG36	Doublesex- and mab-3-related transcription factor 2	Dmrt2
0.0013216	9622100	2	Q8BG60-2;Q8BG60	Q8BG60-2;Q8BG60	Thioredoxin-interacting protein	Txnip
0	969820	6	Q8BG67;Q8BG67-2	Q8BG67;Q8BG67-2	Protein EFR3 homolog A	Efr3a
0	42051000	14	Q8BG81;Q3UDD3;F6VR84	Q8BG81;Q3UDD3;F6VR84	Polymerase delta-interacting protein 3	Poldip3
1	5673000	0	Q8BGB5	Q8BGB5	LIM domain-containing protein 2	Limd2
0.0044861	49063000	6	Q8BGB7-2;Q8BGB7	Q8BGB7-2;Q8BGB7	Enolase-phosphatase E1	Enoph1
0	374510000	251	Q8BGD9	Q8BGD9	Eukaryotic translation initiation factor 4B	Eif4b
0	81263000	26	Q8BGH2	Q8BGH2	[illegible]	Samm50
0	1926700000	629	[illegible]	Q8BGJ5;Q922I7;Q8C8S8;P17225;E9QMW9	Polypyrimidine tract-binding protein 1	Ptbp1
0.0069552	373220	1	Q8BGK6	Q8BGK6	Y+L amino acid transporter 2	Slc7a6
0	444240000	190	Q8BGQ7	Q8BGQ7	Alanine--tRNA ligase, cytoplasmic	Aars
0.0045662	5158500	3	Q8BGS0-2;Q8BGS0	Q8BGS0-2;Q8BGS0	Protein MAK16 homolog	Mak16
0	507510000	224	Q8BH04;Q9Z2V4	Q8BH04	[illegible]	Pck2
0	24657000	16	Q8BH24	Q8BH24	Transmembrane 9 superfamily member 4	Tm9sf4
0	220230000	104	Q8BH40;O70439	Q8BH40;O70439	Syntaxin-7	Stx7
0	27265000	5	Q8BH43;Q8R5H6	Q8BH43;Q8R5H6	[illegible]	Wasf2;Wasf1
0	131060000	54	Q8BH59;V9GXX9	Q8BH59	Calcium-binding mitochondrial carrier protein Aralar1	Slc25a12
0.0044603	42044000	9	Q8BH74;E9Q4V9	Q8BH74;E9Q4V9	Nuclear pore complex protein Nup107	Nup107
0.0047755	7562800	9	Q8BH78;Q8BHF5;Q99P72-1	Q8BH78;Q8BHF5		Rtn4
0	109100000	40	Q9QY76;Q8BH80	Q9QY76;Q8BH80	[illegible]	Vapb
0	69696000	27	Q8BH95;F6T930	Q8BH95;F6T930	Enoyl-CoA hydratase, mitochondrial	Echs1
0	28646000	39	Q8BHC4;B0QZX9	Q8BHC4	Dephospho-CoA kinase domain-containing protein	Dcakd
0	22101000	13	Q8BHC7	Q8BHC7	Rhomboid-related protein 4	Rhbdd1
0	52034000	19	Q8BHD0	Q8BHD0	Ras-related protein Rab-39A	Rab39a
0	35343000	11	Q8BHE0	Q8BHE0	Proline-rich protein 11	Prr11
0.0044823	6327500	2	Q8BHF7;F8WI04	Q8BHF7	[illegible]	Pgs1
0	888340000	320	Q8BHN3;Q8BHN3-2;Q8BHN3-3	Q8BHN3;Q8BHN3-2;Q8BHN3-3	Neutral alpha-glucosidase AB	Ganab
0	80432000	38	Q8BI84;Q8BI84-2;J3KMG3;Q8BI84-3	Q8BI84;Q8BI84-2	Melanoma inhibitory activity protein 3	Mia3
0.0047416	235780	4	Q8BIF9	Q8BIF9	Zinc finger protein 787	Znf787
0	38822000	36	Q8BIG7;H3BJ37	Q8BIG7	[illegible]	Comtd1
0	94341000	37	Q8BIJ6;E9PWN2;E9PWN3	Q8BIJ6	Isoleucine--tRNA ligase, mitochondrial	Iars2
0.0013298	2114600	2	Q8BIW1	Q8BIW1	Protein prune homolog	Prune
0.0044683	5458900	2	Q8BJ03-2;Q8BJ03	Q8BJ03-2;Q8BJ03	[illegible]	Cox15
0.0041442	2998300	1	Q8BJ05-2;Q8BJ05-3;Q8BJ05	Q8BJ05-2;Q8BJ05-3;Q8BJ05	Zinc finger CCCH domain-containing protein 14	Zc3h14
0.0029536	23896000	5	Q8BJ48;D3Z6C9	Q8BJ48;D3Z6C9	[illegible]	Nagpa
0	183350000	77	Q8BJ71;Q8BJ71-2	Q8BJ71;Q8BJ71-2	Nuclear pore complex protein Nup93	Nup93
0	20095000	22	Q8BJS4-3;Q8BJS4-2;Q8BJS4	Q8BJS4-3;Q8BJS4-2;Q8BJS4	SUN domain-containing protein 2	Sun2
0.0046447	3025400	6	Q8BJU0-2;Q8BJU0	Q8BJU0-2;Q8BJU0	[illegible]	Sgta
0	159580000	79	Q8BJW6;D6RFN2;D6RGA6	Q8BJW6	Eukaryotic translation initiation factor 2A	Eif2a
0.0080956	33752000	6	Q8BJW6-2;D3YZZ6	Q8BJW6-2	Eukaryotic translation initiation factor 2A	Eif2a
0	33206000	11	Q8BJY1;F7BA91	Q8BJY1	26S proteasome non-ATPase regulatory subunit 5	Psmd5
0	6761800	14	Q8BJZ4	Q8BJZ4	28S ribosomal protein S35, mitochondrial	Mrps35
0	139810000	57	Q8BK35	Q8BK35		Glscr2
0	258820000	60	Q8BK64	Q8BK64	[illegible]	Ahsa1
0	221520000	98	Q8BK67;A2AWQ2	Q8BK67	Protein RCC2	Rcc2
0	20352000	15	Q8BK72	Q8BK72	28S ribosomal protein S27, mitochondrial	Mrps27
0	415130000	215	Q8BKC5;Q8BKC5-2;Q8BIV3	Q8BKC5;Q8BKC5-2	Importin-5	Ipo5
0	25384000	10	Q8BKE6	Q8BKE6	Cytochrome P450 20A1	Cyp20a1
0	124240000	32	Q8BL66	Q8BL66	Early endosome antigen 1	Eea1
0	126900000	47	Q8BL97-3;Q8BL97-4;Q8BL97-2;Q8BL97	Q8BL97-3;Q8BL97-4;Q8BL97-2;Q8BL97	Serine/arginine-rich splicing factor 7	Srsf7
0	1745300000	513	Q8BLF1;Q8BYQ0;D6RGP7	Q8BLF1	Neutral cholesterol ester hydrolase 1	Nceh1
0.0098897	6567000	7	Q8BLH7	Q8BLH7	HIRA-interacting protein 3	Hirip3
0	37384000	17	Q8BLU0	Q8BLU0		Rrt2
0	10613000	6	Q8BMC4	Q8BMC4	Nucleolar protein 9	Nop9
0	215680000	97	Q8BMD8	Q8BMD8	[illegible]	Slc25a24
0	232420000	96	Q8BMF4	Q8BMF4	[illegible]	Dlat

[illegible]

0	4869400	4	Q9D7M1	Q9D7M1	Glucose-induced degradation protein 8 homolog	Gid8
0	18973000	21	Q9D7N3	Q9D7N3	28S ribosomal protein S9, mitochondrial	Mrps9
0	23390000	4	Q9D7N9	Q9D7N9	Adipocyte plasma membrane-associated protein	Apmap
0	312810000	95	Q9D7S7-2;Q9D7S7	Q9D7S7-2;Q9D7S7	60S ribosomal protein L22-like 1	Rpl22l1
[illegible]002551	1228100	1	Q9D7X8	Q9D7X8	Gamma-glutamylcyclotransferase	Ggct
0	17763000	3	Q9D819	Q9D819	Inorganic pyrophosphatase	Ppa1
[illegible]045851	8467400	17	Q9D823	Q9D823	60S ribosomal protein L37	Rpl37
0	271810000	116	Q9D880	Q9D880	[illegible]	Timm50
0	46335000	31	[illegible]	Q9D883;G3UW94	Splicing factor U2AF 35 kDa subunit	U2af1
0	130560000	20	Q9D8B3	Q9D8B3	Charged multivesicular body protein 4b	Chmp4b
1	0	0	Q9D8C3	Q9D8C3	[illegible]	Alg13
[illegible]047801	9857500	4	Q9D8C4;D6RFB1	Q9D8C4;D6RFB1	Interferon-induced 35 kDa protein homolog	Ifi35
0	6421000000	1018	Q9D8E6	Q9D8E6	60S ribosomal protein L4	Rpl4
0	21444000	10	Q9D8M4	Q9D8M4	60S ribosomal protein L7-like 1	Rpl7l1
0	2172300000	402	Q9D8N0	Q9D8N0	Elongation factor 1-gamma	Eef1g
0	52526000	25	Q9D8S4	Q9D8S4	Oligoribonuclease, mitochondrial	Rexo2
[illegible]046773	13030000	3	Q9D8T2	Q9D8T2	Gasdermin-D	Gsdmdc1
0	476860000	129	Q9D8U8	Q9D8U8	Sorting nexin-5	Snx5
0	214180000	81	Q9D8W5;B1AT36;Q3TRH2	Q9D8W5;B1AT36;Q3TRH2	26S proteasome non-ATPase regulatory subunit 12	Psmd12
0	1622200	4	Q9D8Y1	Q9D8Y1	Transmembrane protein 126A	Tmem126a
0	13308000	12	Q9D937;A0A087WQJ5;A0A087WRC2	Q9D937;A0A087WQJ5;A0A087WRC2		1810009A15Rik
0	340380000	116	Q9D964	Q9D964	Glycine amidinotransferase, mitochondrial	Gatm
[illegible]047037	52077000	6	Q9DAR7;D6RFQ0;Q3T8W9	Q9DAR7;D6RFQ0;Q3T8W9	m7GpppX diphosphatase	Dcps
0	19434000	2	Q9DAU1-2;Q9DAU1	Q9DAU1-2;Q9DAU1	Protein canopy homolog 3	Cnpy3
[illegible]029611	6311000	12	Q9DAY9	Q9DAY9		Npm1
0	108150000	42	Q9DB05;P28663	Q9DB05	Alpha-soluble NSF attachment protein	Napa
0	223640000	96	Q9DB15	Q9DB15	39S ribosomal protein L12, mitochondrial	Mrpl12
0	617430000	214	Q9DB20;F7D3P8;F6XVM5	Q9DB20	ATP synthase subunit O, mitochondrial	Atp5o
0	32221000	21	Q9DB25;D6RCG2	Q9DB25;D6RCG2	Dolichyl-phosphate beta-glucosyltransferase	Alg5
0	217870000	102	[illegible]	Q9DB73;G3UZG6;Q9DB73-2	NADH-cytochrome b5 reductase 1	Cyb5r1
0	1297900000	326	Q9DB77	Q9DB77	Cytochrome b-c1 complex subunit 2, mitochondrial	Uqcrc2
[illegible]047596	981100	4	Q9DB96	Q9DB96	Neuroguidin	Ngdn
0	9830700	2	Q9DBB8	Q9DBB8	Trans-1,2-dihydrobenzene-1,2-diol dehydrogenase	Dhdh
0	80724000	54	Q9DBC7;A2AI69	Q9DBC7	[illegible]	Prkar1a
0	33354000	19	Q9DBD5;Q9D4T3	Q9DBD5;Q9D4T3	Proline-, glutamic acid- and leucine-rich protein 1	Pelp1
0	13435000	6	Q9DBE9	Q9DBE9	pre-rRNA processing protein FTSJ3	Ftsj3
0	7404400	9	Q9DBG1	Q9DBG1	Sterol 26-hydroxylase, mitochondrial	Cyp27a1
0	318380000	131	Q9DBG5	Q9DBG5	Perilipin-3	Plin3
0	982550000	318	Q9DBG6;A2ACG7	Q9DBG6;A2ACG7	[illegible]	Rpn2
0	47993000	36	Q9DBG7;F6VF36	Q9DBG7	Signal recognition particle receptor subunit alpha	Srpr
0	9827700	23	Q9DBH0	Q9DBH0	NEDD4-like E3 ubiquitin-protein ligase WWP2	Wwp2
0	45135000	8	Q9DBH5	Q9DBH5	Vesicular integral-membrane protein VIP36	Lman2
0	2851800000	324	Q9DBJ1;O70250	Q9DBJ1	Phosphoglycerate mutase 1	Pgam1
0	61654000	38	Q9DBL1;E9Q5L3	Q9DBL1;E9Q5L3	[illegible]	Acadsb
0	22469000	12	Q9DBL7	Q9DBL7	[illegible]	Coasy
0	94140000	51	Q9DBP5	Q9DBP5	UMP-CMP kinase	Cmpk1
[illegible]013204	15524000	10	Q9DBR0	Q9DBR0	A-kinase anchor protein 8	Akap8
0	85320000	37	Q9DBR1-2;Q9DBR1	Q9DBR1-2;Q9DBR1	5'-3' exoribonuclease 2	Xrn2
0	255520000	132	Q9DBS1	Q9DBS1	Transmembrane protein 43	Tmem43
0	18479000	9	Q9DBY8	Q9DBY8	Nuclear valosin-containing protein-like	Nvl
0	62529000	39	Q9DBZ5	Q9DBZ5	Eukaryotic translation initiation factor 3 subunit K	Eif3k
0	75402000	20	Q9DC16;F6SLP4	Q9DC16;F6SLP4	[illegible]	Ergic1
0	32002000	15	Q9DC23	Q9DC23	DnaJ homolog subfamily C member 10	Dnajc10
[illegible]013268	56919000	6	Q9DC29	Q9DC29	[illegible]	Abcb6
0	45169000	42	Q9DC37	Q9DC37	[illegible]	Mfsd1
[illegible]001328	115120000	6	Q9DC48	Q9DC48	Pre-mRNA-processing factor 17	Cdc40
0	49373000	33	Q9DC51	Q9DC51	Guanine nucleotide-binding protein G(k) subunit alpha	Gnai3
0	114410000	74	[illegible]	Q9DC53	Copine-8	Cpne8
0	13846000	20	Q9DC61;A2AIW9	Q9DC61;A2AIW9	Mitochondrial-processing peptidase subunit alpha	Pmpca
0	155010000	121	Q9DC69	Q9DC69	[illegible]	Ndufa9
0	109730000	50	Q9DCA5;F6WEP7	Q9DCA5	Ribosome biogenesis protein BRX1 homolog	Brix1
0	1037800000	298	Q9DCD0	Q9DCD0	6-phosphogluconate dehydrogenase, decarboxylating	Pgd
[illegible]047015	12256000	4	Q9DCD6;B1AR50;Q8R3R8	Q9DCD6;B1AR50;Q8R3R8	[illegible]	Gabarap;Gabarapl1
0	60453000	28	Q9DCF9-2;Q9DCF9	Q9DCF9-2;Q9DCF9	Translocon-associated protein subunit gamma	Ssr3
0	229490000	96	Q9DCH4	Q9DCH4	Eukaryotic translation initiation factor 3 subunit F	Eif3f
[illegible]045167	15606000	7	Q9DCJ5	Q9DCJ5	[illegible]	Ndufa8

[illegible]
