# Supplementary material for: Molecular Mechanisms of Intracellular Delivery of Nanoparticles Monitored by an Enzyme-Induced Proximity Labeling
Source: Nanomicro Lett. 2024 Feb 1;16:103. doi: 10.1007/s40820-023-01313-0 (PMC10834923; doi:10.1007/s40820-023-01313-0)
Supplement: Supplementary file 3 — (PDF 1087 KB) [file 40820_2023_1313_MOESM3_ESM.pdf]

Table S2 True positive proteins at 0 min, 5 min, 10 min, 20 min, 30 min, 60 min

ProteinIDs	MajorityproteinI	Proteinna	Genenam	HRP(+)-PNP 0min-1 LFQintensityA1	HRP(+)-PNP 0min-2 LFQintensityA2	HRP(-)-PNP 0min-1 LFQintensityA3	HRP(-)-PNP 0min-2 LFQintensityA4	HRP(+)-PNP 5min-1 LFQintensityB1	HRP(+)-PNP 5min-2 LFQintensityB2	HRP(-)-PNP 5min-1 LFQintensityB3	HRP(-)-PNP 5min-2 LFQintensityB4	HRP(+)-PNP 10min-1 LFQintensityC1	HRP(+)-PNP 10min-2 LFQintensityC2	HRP(-)-PNP 10min-1 LFQintensityC3	HRP(-)-PNP 10min-2 LFQintensityC4	HRP(+)-PNP 30min-1 LFQintensityE1	HRP(+)-PNP 20min-2 LFQintensityD2	HRP(-)-PNP 20min-1 LFQintensityD3	HRP(-)-PNP 20min-2 LFQintensityD4	HRP(+)-PNP 20min-1 LFQintensityD1	HRP(+)-PNP 30min-2 LFQintensityE2	HRP(-)-PNP 30min-1 LFQintensityE3	HRP(-)-PNP 30min-2 LFQintensityE4	HRP(+)-PNP 60min-1 LFQintensityF1	HRP(+)-PNP 60min-2 LFQintensityF2	HRP(-)-PNP 60min-1 LFQintensityF3	HRP(-)-PNP 60min-2 LFQintensityF4	Proteinnames	Subcellular location [CC]	Organism	molecular function	Relative abundance 0 min	5 min	10 min	20 min	30 min	60 min	cluster-10
P21447;P06795;P21	P21447;P06795	Multidrug	Abcb1a;A	1841400	933710	0	0	1621500	1623600	0	1221600	1310300	931610	1295900	913930	645500	0	0	0	749810	0	1180700	555310	2529400	602410	0	1970000	Multidrugresistanceprote	Cell membrane; Multichannel membr	Mus musculus (Mouse)	translocase	0.49493	1.06088	-0.1471	-1.2922	-1.041	0.92446	1
[illegible]	[illegible]	Bcl-2hom	Bak1	4812000	4963200	1481500	8073900	8871900	7047500	3631600	6313100	6354600	6067400	1636300	2438600	4891200	0	5672900	0	2749600	4661800	4397000	1511400	5419300	5455300	0	0	Bcl-2homologousantagor	Mitochondria; Endoplasmic reticulum;	Mus musculus (Mouse)	Bcl-2 homologous antagonist/killer	-0.4328	1.68484	0.47947	-0.4303	-1.2474	-0.0539	1
Q8JZN5	Q8JZN5	Acyl-CoA	Acad9	2545100	3680900	2442900	3015300	2871000	4191100	2212800	3039600	2901600	3765200	2766800	2437100	2513700	1625300	4595300	2112900	2816900	2256300	4086900	857190	3492700	2035000	1231200	1187900	Acyl-CoAdehydrogenase	Mitochondria;	Mus musculus (Mouse)	oxidoreductase	0.40882	1.17948	0.81512	-1.5148	-0.6538	-0.2348	1
[illegible]	[illegible]	Coiled-co	Ccdc47	9422800	7036800	5135300	3016100	9772100	9224400	3482800	5110100	7638500	8047300	5244000	3589800	8221800	5192800	8123700	4029200	2316700	2569500	1630700	3866800	9519000	7235600	4171000	4919800	Coiled-coildomain-cont	Endoplasmic reticulum membrane;	Mus musculus (Mouse)	PAT complex subunit CCDC47	0.42029	0.92962	0.26493	-0.1911	-1.9033	0.47951	1
[illegible]	[illegible]	Proteaso	Ecm29	2879500	2017900	1780100	677240	3739900	1854400	1808600	1084800	2424600	2030000	1316600	1182500	2874500	1338100	2880800	1266600	2320500	2651200	2371700	568820	3059700	2595300	1544700	864970	Proteasome-associated	Cytoskeleton; Endocrine body;	Mus musculus (Mouse)	proteinase	-0.1146	1.07944	-0.8732	-1.2878	0.01274	1.18343	1
[illegible]	[illegible]	ERmemb	Emc1	6986500	6053200	5644200	3638300	6733600	5946900	5924400	6396500	6794300	6310700	5519600	3931300	5925300	1622200	6996800	4526100	4487100	4486000	6720700	2077500	7797600	4517200	0	3197200	ERmembraneprotein	endoplasmic reticulum	Mus musculus (Mouse)	ER membrane protein complex subunit 1	0.73567	0.58578	0.76292	-1.5562	-0.9613	0.43317	1
Q99JY0;D3YXU1	Q99JY0;D3YXU1	Trifunctio	Hadhb	5396800	9649900	8894400	6857000	7012200	11733000	7159000	10191000	8834400	10776000	9277800	7396100	5868300	3454900	6222600	3271800	4985300	5487100	11659000	4602300	6837300	10781000	3363700	5382400	Trifunctionalenzymesub	Endoplasmic reticulum; mitochondria	Mus musculus (Mouse)	Trifunctional enzyme subunit β, mitochondri	-0.0205	0.82907	1.02782	-1.3353	-1.0713	0.57021	1
[illegible]	[illegible]	Probable	Lmbrd1	0	0	0	3550400	1771900	5692300	0	2470600	1971100	3764000	1753500	2015200	0	0	0	0	0	0	1713000	0	0	2213700	0	1685700	Probablelysosomalcobal	Lysosome; Endoplasmic reticulum	Mus musculus (Mouse)	[illegible]	-0.8745	1.34743	0.83271	-0.8745	-0.8745	0.44343	1
[illegible]	[illegible]	Aldehyde	Aldh3b1	1706900	1798200	0	0	1571200	1677500	0	1624500	1795600	0	0	1759200	0	0	0	1875600	0	0	0	0	2003000	0	0	0	Aldehydedehydrogenase	Cell membrane; It mainly exists in	Mus musculus (Mouse)	oxidoreductase	0.59576	0.45855	0.64183	-1.28	-1.28	0.86381	1
D3YU17;Q8VCM8	D3YU17;Q8VCM	Nicalin	Ncln	1844100	4404800	1781600	1394400	3749100	3141900	1522800	2107600	3971700	3364200	2749000	0	1586100	0	2698800	0	942260	1051200	2082900	0	4724300	2944300	0	0	Nicalin	Endoplasmic reticulum membrane;	Mus musculus (Mouse)	Components of ribosome-related transpor	0.29323	0.56328	0.7504	-1.0007	-1.4965	0.89032	1
O35604	O35604	Niemann	Npc1	1806200	4094400	1668400	1663200	5476600	4275500	1441400	2335100	3054600	4902100	2382700	1366300	4583600	1801100	1566500	927870	1502000	2399000	2677000	1276200	2288800	4163100	1122400	1425700	Niemann-PickC1protein	Endocrine body; Lysosome; Cell	Mus musculus (Mouse)	transport protein	-0.4169	1.53209	0.62354	-0.172	-1.4288	-0.138	1
[illegible]	P61620	Proteintr	Sec61a1	5453900	8019000	2558400	1688700	7606300	4551300	3596600	2383500	6821900	6069100	1235400	1651700	3808200	1761600	6860700	0	2645900	3510500	1849000	823970	8078700	2579100	0	0	ProteintransportproteinS	Endoplasmic reticulum; Phagocytic	Mus musculus (Mouse)	Developmental protein; transport protein	0.96082	0.58039	0.79251	-1.325	-1.1553	0.14661	1
O55242;O55242-2	[illegible]	Sigmano	Sigmar1	4578700	2354100	0	2772500	4425400	2746800	2739000	2883500	3208200	2224200	2507000	2414300	3023700	1683300	1706200	2252700	2819800	2589100	1878000	0	3399100	2781100	0	0	Sigmanon-opioidintrace	Endoplasmic reticulum; Cell membrane	Mus musculus (Mouse)	receptor	0.99977	1.24894	-0.5619	-1.3169	-0.5864	0.21644	1
P46978;D3YZN5	P46978	Dolichyl	Stt3a	12692000	9950400	9729700	4953700	16557000	7674200	11902000	12441000	16273000	9451000	8842200	11200000	11378000	7128300	10952000	14664000	10473000	6387800	11758000	10515000	16719000	10731000	8590500	6044600	Dolichyl-diphosphooligo	endoplasmic reticulum	Mus musculus (Mouse)	Glycosyl transferase	0.01771	0.40162	0.76233	-0.9817	-1.3793	1.17939	1
Q64310;E0CXD9	Q64310	Surfeitlo	Surf4	4374700	3105600	2188200	1857700	4922700	2154600	2802000	3272900	3656200	2090200	3319000	1298300	2898200	2024300	3639000	0	1638900	3427100	2903400	1229100	5011600	2469100	1022100	2585100	Surfeitlocusprotein4	Endoplasmic reticulum; Golgi apparatus	Mus musculus (Mouse)		0.99298	0.65522	-0.4602	-1.1508	-1.0305	0.99331	1
P58681	P58681	Toll-like	Tlr7	4620900	3832600	4524800	3485100	3833000	0	2658700	4522900	3973900	2588900	3604400	4085300	3314100	3302100	0	2973200	3192200	2549500	3238900	3448200	5081000	3046100	3267100	3908800	Toll-likereceptor7	Lysosome; Endoplasmic reticulum;	Mus musculus (Mouse)	receptor	1.19805	0.44864	-0.6012	-0.5504	-1.3826	0.88744	1
[illegible]	[illegible]	Transpor	Tnpo3	2817900	2958800	1052700	1141800	2263900	1858600	1121200	1094700	1516600	1811000	1206900	1782900	1045900	0	1894300	0	1220900	1915400	1858600	1196600	3022000	2129700	0	0	Transportin-3	Nucleus; Cytoplasm; It is located in	Mus musculus (Mouse)	transport protein	1.34997	0.13781	-0.4447	-1.3502	-0.5849	0.89199	1
Q9ER38	Q9ER38	Torsin-3	Tor3a	4003100	3043900	1797200	2682700	2642600	3733700	2768300	2967700	2639100	2001200	4174300	2013100	3415400	1769600	1651400	0	2500200	3359600	1990300	1545200	0	3229200	0	2532000	Torsin-3A	endoplasmic reticulum	Mus musculus (Mouse)	Torsin-3A; Atpase activity	1.25931	0.50464	-1.4487	-0.8358	-0.0765	0.59702	1
[illegible]																																						

Q9QYR9;O55137	Q9QYR9;O5513	[illegible]	Acot2;Ac	1759700	2246700	896020	1054600	2632200
P68033;P68134;P63	P68033;P68134;	[illegible]	Actc1;Act	101600000	154010000	47089000	42847000	54913000
D3Z0B9;Q571I9;F6R	D3Z0B9;Q571I9	[illegible]	Aldh16a1	3540200	2313000	2717500	0	2745400
F8QEH3;E9PZE5;P9	F8QEH3;E9PZE5	[illegible]	Anp32e	4727700	5860400	2795100	0	3989500
Q61210-2;Q61210	Q61210-2;Q612	[illegible]	Arhgef1	2818500	2573700	2848400	0	2150600
Q9Z1G3	Q9Z1G3	[illegible]	Atp6v1c1	9216000	7944700	6386200	5300100	6313200
P57746	P57746	[illegible]	Atp6v1d	1838000	2270000	2043800	0	2227200
Q5SUH7;Q99KN9;Q	Q5SUH7;Q99KN	[illegible]	Clint1	7429900	10135000	6380600	8057700	6341000
Q6P8N8;Q9JHS4	Q6P8N8;Q9JHS4	[illegible]	Clpx	809770	813080	1484500	978690	862940
Q9DBL7	Q9DBL7	[illegible]	Coasy	1605500	1655700	0	1131600	0
Q9DB73;G3UZG6;Q	Q9DB73;G3UZG	[illegible]	Cyb5r1	9185400	14855000	3661900	4536600	10937000
P54823	P54823	[illegible]	Ddx6	6751700	6918000	5559000	0	6008400
O88508;G3UX43;G3	O88508;G3UX43	[illegible]	Dnmt3a	1436000	1182300	0	969310	1341900
Q99JX4;A2A701;A2	Q99JX4;A2A701;	[illegible]	Eif3m	7872000	7704300	1897900	2612100	4258600
Q76LS9-2;Q76LS9	Q76LS9-2;Q76L	[illegible]	Fam63a	1815200	1046800	1520700	0	0
Q62446	Q62446	[illegible]	Fkbp3	2238100	3725500	2551200	3485700	2647900
P77NI1;A0A087WSF	P77NI1;A0A087	[illegible]	Fn1	1280300	905100	0	0	0
P84078;P61205;D3	P84078;P61205	[illegible]	Arf1;Arf3	38157000	46476000	15172000	24619000	28263000
Q9CPU0	Q9CPU0	[illegible]	Glo1	4384800	4964100	1222300	6203200	2404000
P62331	P62331	[illegible]	Arf6	4166100	3765400	1194100	1482300	2287200
Q91YI0;E0CY49;E0	Q91YI0;E0CY49;	[illegible]	Asl	3901000	2062100	982160	1157300	2435000
Q64521;A2AQR0	Q64521;A2AQR	[illegible]	Gpd2	0	1274900	0	878650	1149300
Q8VE99	Q8VE99	[illegible]	Ccdc115	0	2432000	0	2721700	1650100
Q9QXK3-4;Q9QXK	Q9QXK3-4;Q9Q	[illegible]	Copg2	64269000	64452000	62768000	16806000	65491000
Q99KV1	Q99KV1	[illegible]	Dnajb11	3948800	5144200	4453400	9400500	2816400
Q9EQ06;Q9EQ06-2	Q9EQ06	[illegible]	Hsd17b1	2333000	3203900	1799500	1450500	2323300
Q8VD75	Q8VD75	[illegible]	Hip1	0	0	1417500	0	1284300
Q921F4;Q921F4-4	Q921F4;Q921F4	[illegible]	Hnrnpll	1406000	0	0	1272200	1363600
Q9DBH5	Q9DBH5	[illegible]	Lman2	0	0	0	0	0
Q99M31;Q99M31-2	Q99M31;Q99M3	[illegible]	Hspa14	1641500	1630400	0	0	0
P70404	P70404	[illegible]	Idh3g	5387100	8454100	7376400	3052200	4699200
P11928;Q8K469;Q0	P11928;Q8K469	[illegible]	Oas1a;O	5275600	4257500	4942900	2964400	3468900
Q80UJ7	Q80UJ7	[illegible]	Rab3gap	1288900	523700	1059100	0	1377300
Q9JHL0-2;Q9JHL0	Q9JHL0-2;Q9JH	[illegible]	Lat2	1867900	2654100	2380200	3689100	2282300
P17427	P17427	[illegible]	Ap2a2	1458700	1881800	1372400	0	1541200
D3YXW1;Q8R045-2	D3YXW1;Q8R04	[illegible]	Llph	767140	2966700	4651100	0	2132700
Q9QYA2;G3UY77	Q9QYA2	[illegible]	Tomm40	7359800	11230000	4630700	0	7638100
F8Q8P6;Q8VCW4;F	F8Q8P6;Q8VCW	[illegible]	Unc93b1	4873200	6184800	3756100	2959100	5563200
Q97170-4;Q97170	Q97170-4;Q971	[illegible]	Uso1	2699300	3657800	2284200	0	2643000

Q3TTY5;CON__Q3T[illegible]	Q3TTY5;CON__([illegible]	Keratin [illegible]	Krt2	3640200	3369500	0	0	0
P06151-GFF8NF-D3[illegible]	P06151;GFF8NF[illegible]	L-lactate[illegible]	Ldha	443650000	581720000	191360000	168940000	275700000
P16045	P16045	Galectin[illegible]	Lgals1	23970000	41147000	18021000	43433000	10602000
Q99N69	Q99N69	Lupaxin[illegible]	Lpxn	6093400	7268000	2493100	3342700	4805000
E0CZE0;Q8VBW6	E0CZE0;Q8VBW[illegible]	NEDD8[illegible]	Nae1	1188200	1797800	0	0	0
Q99KQ4	Q99KQ4	Nicotinar[illegible]	Nampt	16485000	18286000	10664000	6699500	11876000
Q62433;E9Q514;E9[illegible]	Q62433	Protein N[illegible]	Ndrg1	14807000	13290000	8161800	7616200	6536500
Q9CZ30;Q9CZ30-2[illegible]	Q9CZ30;Q9CZ3[illegible]	Obg-like[illegible]	Ola1	5874600	8789300	3094700	4368100	3957700
P97369;A8XU21	P97369;A8XU21	Neutroph[illegible]	Ncf4	4967200	6890900	3025600	2833000	3984900
P12382	P12382	6-phosph[illegible]	Pfkl	6738800	7780000	2081200	0	2862500
Q9D819	Q9D819	Inorganic[illegible]	Ppa1	0	3376100	0	0	0
P68372	P68372	Tubulin b[illegible]	Tubb4b	79594000	98071000	52270000	52711000	64450000
Q64514-2;Q64514[illegible]	Q64514-2;Q645[illegible]	Tripeptid[illegible]	Tpp2	10541000	8812900	4883000	2036100	5479000
P68368;A0A0A0MQ[illegible]	P68368;A0A0A0[illegible]	Tubulin al[illegible]	Tuba4a	7029500	6967400	5155600	6072200	7300600
F6UFG6;D3YYE1;D3[illegible]	F6UFG6;D3YYE1[illegible]	Acidic leu[illegible]	Anp32a	2146500	5154500	0	1978000	2196600
Q8R180;Q8R2E9	Q8R180	ERO1-lik[illegible]	Ero1l	29556000	27192000	9990700	9280800	16826000
Q9DC51	Q9DC51	Guanine[illegible]	Gnai3	3856000	3015100	5109400	2427000	2116600
Q9D0I9	Q9D0I9	Arginine[illegible]	Rars	46291000	43209000	22620000	6470400	42485000
Q8BMJ2	Q8BMJ2	Leucine[illegible]	Lars	38713000	35252000	15384000	572830	33064000
Q9ERE7;D3YVR4	Q9ERE7;D3YVR[illegible]	LDLR cha[illegible]	Mesdc2	1533900	2504200	0	1538700	1230000
P63005;P63005-2;[illegible]	P63005;P63005[illegible]	Platelet[illegible]	Pafah1b1	8410400	6443500	5188900	0	2811100
Q9DCD0	Q9DCD0	6-phosph[illegible]	Pgd	92796000	62117000	42210000	24008000	60395000
P52480	P52480	Pyruvate[illegible]	Pkm	1232500000	1183300000	514100000	436060000	907320000
P62830;A2A6F8	P62830	60S ribos[illegible]	Rpl23	39917000	49928000	20187000	17470000	28422000
P62889	P62889	60S ribos[illegible]	Rpl30	22674000	25362000	12198000	16936000	15840000
Q80YR5	Q80YR5	Scaffold[illegible]	Safb2	440510	387250	0	0	0
Q64337-2;Q64337[illegible]	Q64337-2;Q643[illegible]	Sequesto[illegible]	Sqstm1	21638000	24540000	5365600	3995500	8232800
Q91YJ2	Q91YJ2	Sorting n[illegible]	Snx4	1796600	1204000	1641300	821900	982850
Q9D0R2	Q9D0R2	Threonin[illegible]	Tars	28341000	25515000	13867000	6410700	20226000
Q9CZW5	Q9CZW5	Mitochon[illegible]	Tomm70	4315300	2107500	3389300	3257200	0
A2AN08-2;A2AN08[illegible]	A2AN08-2;A2AN[illegible]	E3 ubiquit[illegible]	Ubr4	837380	759420	0	0	760070
P32921-2;P32921	P32921-2;P329[illegible]	Tryptoph[illegible]	Wars	28170000	25014000	7705600	7396100	18593000
P61222	P61222	ATP-bin[illegible]	Abce1	10195000	9539800	6818500	4131500	9938200
Q6P542	Q6P542	ATP-bin[illegible]	Abcf1	3257900	6556400	3273800	3526100	2566200
Q99JW2	Q99JW2	Aminoac[illegible]	Acy1	1043700	0	0	1049500	1085800
Q9JII6;B1AXW3	Q9JII6	Alcohol d[illegible]	Akr1a1	14812000	24565000	9357600	15692000	15280000
P45377	P45377	Aldose r[illegible]	Akr1b8	12463000	22000000	5561700	7212800	9212500
P05064;A6ZI44;D3Y[illegible]	P05064;A6ZI44;[illegible]	Fructose[illegible]	Aldoa;Al[illegible]	556530000	832620000	300970000	378580000	430880000
Q35639	Q35639	Annexin A[illegible]	Anxa3	25213000	41901000	17651000	32430000	26685000

[illegible]
