# Supplementary material for: Molecular Mechanisms of Intracellular Delivery of Nanoparticles Monitored by an Enzyme-Induced Proximity Labeling
Source: Nanomicro Lett. 2024 Feb 1;16:103. doi: 10.1007/s40820-023-01313-0 (PMC10834923; doi:10.1007/s40820-023-01313-0)
Supplement: Supplementary file 4 — (PDF 400 KB) [file 40820_2023_1313_MOESM4_ESM.pdf]

Table S3 True positive proteins classified according to organelle function

Early endosome

Genenames	LFQintensityA1	LFQintensityA2	LFQintensityB1	LFQintensityB2	LFQintensityC1	LFQintensityC2	LFQintensityE1	LFQintensityD2	LFQintensityD1	LFQintensityE2	LFQintensityF1	LFQintensityF2
Sh3gl1;Sh3gl2	1410000	2071200	1065000	2225300	0	0	722520	0	0	0	1092100	0
Rab31	1276500	2070900	0	2305300	0	1672700	2265300	0	1399100	1677200	0	1723500
Eea1	4323900	7484900	4655700	13537000	4523900	9336000	3052900	5319300	4091800	7340900	4306800	3873600
Vps16	2164600	1874500	2854000	0	2149200	2125600	2006800	0	1670200	1560700	1902900	1854900
Wdfy4	754250	619620	707670	2540400	596870	560890	640560	522690	0	1136600	924500	672470
Ehd1	15452000	17264000	11919000	14249000	13252000	11559000	11425000	10703000	13524000	15589000	9707100	15315000
Snx4	1796600	1204000	982850	1309600	967650	1036200	879130	995510	911320	957050	1090600	908030
Derl1	2186100	2649900	2749100	2174600	2927900	2324700	3488500	2704800	2095800	2777900	3464000	2957600
Rftn1	6245300	6289600	3072300	5441200	7516400	6757500	6558900	8358200	5126500	4142700	7014400	4927500
Vps26a	3278800	4662200	3050400	5536100	3431300	4091300	5402400	0	3119900	3121300	2600700	5320400
Arf6	4166100	3765400	2287200	4226600	3910100	3631300	1736800	1331700	3935700	2004200	4729700	1465100
Rab21	3704900	2463500	0	3743600	4309800	0	4068700	4255400	4751700	4109100	3675500	2479700
Coro1a	290060000	232450000	127270000	196570000	199760000	226010000	127200000	163060000	265350000	273870000	193300000	144190000
Nae1	1188200	1797800	0	0	1070300	1256500	0	0	1104900	1095300	1278600	1207100
Mapk3	2465500	4422500	2267600	4330700	2869700	4109000	1797400	3297200	4607600	4664200	2111200	0

Late endosome

Genenames	LFQintensityA1	LFQintensityA2	LFQintensityB1	LFQintensityB2	LFQintensityC1	LFQintensityC2	LFQintensityE1	LFQintensityD2	LFQintensityD1	LFQintensityE2	LFQintensityF1	LFQintensityF2
Tcirg1	0	4029900	2806800	4617600	0	2863600	0	0	0	0	3486400	3399600
Vps16	2164600	1874500	2854000	0	2149200	2125600	2006800	0	1670200	1560700	1902900	1854900
Ap1b1	8705000	9064100	10646000	11174000	8279400	9118600	6497800	3233300	7441200	9881900	7333900	9535900
H1f0	1878400	2732500	3379200	5658500	0	1776900	3755700	0	5234500	4563700	3378700	2904200
Rab21	3704900	2463500	0	3743600	4309800	0	4068700	4255400	4751700	4109100	3675500	2479700
Gapdh;Gm7293	1406900000	1725500000	984950000	1229900000	1140300000	1211200000	947860000	887680000	1390900000	1269300000	1161700000	936630000
Sqstm1	21638000	24540000	8232800	17240000	13096000	11874000	14420000	15039000	21147000	21866000	8531000	11225000
Lamtor1	1363800	0	0	4132100	1656500	1715400	1383000	0	1324900	1432200	1423300	1504800
Anxa6	2756100	8272100	2999200	6110800	2751400	3638300	3070200	4308300	5828900	6102400	2366600	3285800
Chmp4b	5806300	8059500	3182800	9463300	5235700	9779200	5206300	7749800	14771000	13246000	3084800	5507500
Mapk3	2465500	4422500	2267600	4330700	2869700	4109000	1797400	3297200	4607600	4664200	2111200	0
Grn	0	2400500	2286600	2428200	0	2198400	3097400	2413800	0	2283900	0	0
Derl1	2186100	2649900	2749100	2174600	2927900	2324700	3488500	2704800	2095800	2777900	3464000	2957600
Vti1b	0	2016700	1904800	2393800	1600600	2633400	2184200	2410300	2188000	2077000	0	2138700
	1459568700	1798025700	1028258900	1303367200	1185176200	1265357100	998836000	933091900	1461960800	1343865000	1202458300	983423700

Lysosome

Genenames	LFQintensityA1	LFQintensityA2	LFQintensityB1	LFQintensityB2	LFQintensityC1	LFQintensityC2	LFQintensityE1	LFQintensityD2	LFQintensityD1	LFQintensityE2	LFQintensityF1	LFQintensityF2
Lmbrd1	0	0	1771900	5692300	1971100	3764000	0	0	0	0	0	2213700
Npc1	1806200	4094400	5476600	4275500	3054600	4902100	4583600	1801100	1502000	2399000	2288800	4163100
Sec61a1	5453900	8019000	7606300	4551300	6821900	6069100	3808200	1761600	2645900	3510500	8078700	2579100
Sigmar1	4578700	2354100	4425400	2746800	3208200	2224200	3023700	1683300	2819800	2589100	3399100	2781100
Tlr7	4620900	3832600	3833000	0	3973900	2588900	3314100	3302100	3192200	2549500	5081000	3046100
Btk	3999800	3556900	3541000	3073900	2802500	2867900	2813900	2119100	1723500	1977000	3609700	2478700
Gpnmb	8067800	6282700	10862000	13876000	12106000	17295000	5013000	2807500	5956500	3814700	7615200	5293400
Cltc	337320000	263460000	307880000	142410000	350650000	250570000	252060000	225510000	219680000	223900000	344470000	268440000
Sh3gl1;Sh3gl2	1410000	2071200	1065000	2225300	0	0	722520	0	0	0	1092100	0
Tcirg1	0	4029900	2806800	4617600	0	2863600	0	0	0	0	3486400	3399600
Fcgr3	2691400	0	13818000	0	0	0	0	0	0	0	8202200	10093000
Atp6v0c	5003300	7807700	6948700	11104000	6832100	10306000	5136400	6289000	6100000	6907600	6888700	5157900
Tlr13	3257900	1883800	2435800	3111900	2695300	2096500	1847400	0	1894000	1199600	3038000	1762500
H2-D1	247470000	171050000	183720000	139460000	176280000	133870000	178310000	192490000	132440000	93152000	223630000	133140000
H2-L	217200000	106510000	143450000	64349000	126340000	64055000	129410000	146350000	87362000	67044000	140870000	113350000
Atp6v0a1	2233200	1783200	2530200	1944900	2829200	2348600	2664600	2325700	2080400	0	3899000	2364400
Cdipt	3253100	3182400	3402600	3815000	3589100	3703700	3864900	2657100	3272200	2013100	5665800	3672700
H2-K1	33131000	31238000	29815000	32153000	24174000	29010000	35687000	25721000	26458000	26083000	35321000	34761000
Gak	0	106360	0	0	96880	135260	106800	0	56836	0	219040	0
Pgrmc1	0	2554400	2104800	3043100	2042600	3232500	3119200	0	0	2042800	1746100	2966300
Scfd1	3766300	3455600	4940900	2958700	5264600	4644800	5211000	4057800	3538800	3344300	3447300	3411400
Dync1li1	2059600	2069100	2209300	2498700	2390000	2149800	2411700	2697700	1521600	2406400	2785300	3046900
Gm15448	1097700	470750	3139500	540430	642700	705390	2149500	156770	389030	386720	2279700	1257000
Rab31	1276500	2070900	0	2305300	0	1672700	2265300	0	1399100	1677200	0	1723500
Neo1	7006700	2820800	17118000	5285000	7624900	3792900	12459000	9662900	1801800	2329400	13791000	11717000
Nrcam	6575500	3840400	8845900	4261600	5464600	4166900	7145200	5318800	2743800	2222500	10252000	4468800
Tm9sf4	1428900	1812800	1586000	1503100	1548700	1506600	1600200	0	1359800	0	1529300	0
Apobr	1406300	3740900	1890500	6167200	0	3285500	2311600	2201900	1769100	3196900	1733500	3070600
Arl1	1376900	3433800	1603700	2757800	1405100	0	1365700	0	1028300	0	0	1089800
Snap23	0	0	0	1619100	1519500	1550500	0	0	0	0	0	0
Arf5	3451400	3100700	0	3237200	0	0	3387000	2845700	2883500	0	0	0
Glb1	1213400	2198700	1020000	3627700	1505100	1778200	604110	0	0	570420	1705500	640000
Srpr	4377500	2565800	3208800	4512200	2921600	2305200	1417500	3169300	1695200	0	2199300	2529200
Stx16	205120	0	0	840320	0	512250	0	0	87848	86104	182680	0
Tmed5	0	1563200	0	5279600	803560	1292500	0	0	0	0	0	0
Fyn	0	333360	977580	758830	661590	719230	668230	0	0	398280	0	0
Gba	3174100	4659500	3984600	7737000	2884600	5693000	1985600	0	1177000	1708300	3875300	1113400
Eea1	4323900	7484900	4655700	13537000	4523900	9336000	3052900	5319300	4091800	7340900	4306800	3873600
Ap1b1	8705000	9064100	10646000	11174000	8279400	9118600	6497800	3233300	7441200	9881900	7333900	9535900
Actr1b	1191300	1433400	1485500	3859500	1323300	1579700	0	0	803430	1218600	1153900	0
Blmh	4145900	2636100	0	7134800	0	3900900	3774600	0	4869300	0	4231800	0
Gabarap;Gabarapl1	0	0	0	1212100	0	1083100	0	0	1047600	758020	0	0
Serpinb6;Serpinb6a	14681000	12073000	15041000	15016000	14945000	13380000	11706000	10785000	11825000	14248000	13322000	12589000
Slc4a7	1337900	1477400	963370	1742700	970950	943410	1617600	970540	730750	965910	971300	833760
Snx12	869700	1980100	0	2239900	835630	1122400	751440	601940	1069000	1074400	0	834970
Snx9	2653500	1686500	2292300	0	1986300	1597800	0	2142400	1857800	2064000	1723400	2090900
Tsnax	441620	975270	387210	1696500	557790	1216700	455700	0	264540	355570	0	0
Wdfy4	754250	619620	707670	2540400	596870	560890	640560	522690	0	1136600	924500	672470
Cops2	1967100	3069800	1803400	3642100	1697600	3403700	1639300	1357200	3040400	1582200	0	1129400
Coro1b	2424400	5430900	0	5436600	2247000	3566200	0	0	1954400	4262000	2325200	0
Ppt1	0	6352000	4313300	7494500	4248200	3979600	3325400	2942200	4255700	5091200	0	3763500
Dnm1l	3890600	3125600	4508300	3276400	2653300	2691000	1939800	0	2854600	4216000	3208900	0
Anxa5	170480000	308090000	187920000	331330000	174600000	228950000	154800000	126090000	205750000	256070000	126400000	188400000
Osbp	931830	1104600	960400	2152400	1076700	1113200	0	0	1159700	0	0	0
Park7	2728100	5488000	4199200	6036800	3750400	4729000	1748300	4784400	4486100	3798600	1964300	3397200
Rhoa	1780900	5543800	1586100	5352600	2441000	4192200	1642700	2524500	2757600	3097600	2922500	2148900
Rraga;Rragb	2526100	2387300	2344100	3171200	2084000	3250200	2050200	0	1287300	1655000	2231200	2578000
Otub1	5271100	4607200	4635600	7184300	3453700	3968100	2329600	2420900	3893900	5571700	2639900	2862700
Plek	8635200	12689000	3632400	15386000	7806000	8228100	2775700	2433300	7610200	5953200	4998100	3253000
Lamtor1	1363800	0	0	4132100	1656500	1715400	1383000	0	1324900	1432200	1423300	1504800
Ube2l3	7468800	13854000	5528700	15918000	7733700	9686100	4633800	4237000	8077100	7265300	6456700	4267300
Rragc;Rragd	1697300	3817300	1837600	6193900	2085300	2326500	0	0	3337300	2534000	2309600	0
Smap;1110004F10Rik	0	3437800	0	6294400	0	2745000	3034100	2926400	3756400	4417300	0	0
Twf2	2167300	3813200	2133300	3977000	2205000	2361400	1944600	1456000	2051900	2619900	1931600	1882600
Rab35	0	1443600	1922300	1789300	0	1328000	0	0	1489800	1430800	0	0
Vav3	0	0	1507800	0	1457000	1363700	0	0	1233000	0	0	0
Arhgef1	2818500	2573700	2150600	2224900	3357500	2468100	2059700	2126600	2566600	2314100	2130900	0
Atp6v1c1	9216000	7944700	6313200	9451200	10217000	8635900	7403400	7978500	7312800	6432500	9535000	6796000
Atp6v1d	1838000	2270000	2227200	4042500	2622800	2436000	0	0	2308200	2512100	2047600	3317100
Clint1	7429900	10135000	6341000	10858000	9370000	11464000	5930200	6944500	7558700	6088700	5774400	4900600
Arf6	4166100	3765400	2287200	4226600	3910100	3631300	1736800	1331700	3935700	2004200	4729700	1465100
Arf1;Arf3	38157000	46476000	28263000	52977000	34178000	39371000	17060000	14337000	28387000	24101000	49669000	17520000
Ccdc115	0	2432000	1650100	2575700	1751000	2014000	1598000	1369600	1629600	1798600	1745400	1756800
Copg2	64269000	64452000	65491000	53015000	50822000	61088000	31171000	45801000	44083000	47688000	56491000	40133000
Hip1	0	0	1284300	994040	1321900	0	0	0	989450	935460	1125100	0
Lman2	0	0	0	2716000	2111100	0	0	0	1863500	0	1985900	2086700
Rab3gap1	1288900	523700	1377300	1283500	1163800	1375100	0	0	1281000	1211800	1077400	0
Ap2a2	1458700	1881800	1541200	1425900	1563800	1540200	1474500	1011400	1474600	1447200	0	1450300
Unc93b1	4873200	6184800	5563200	4755400	6240100	5183700	4112400	4786800	5642400	3460500	5303300	4078900
Uso1	2699300	3657800	2643000	3043000	3054400	4261100	2512400	2541000	2764200	2731300	3348000	2055600
Naga	843430	841900	415380	1101300	0	928520	455080	0	344500	161240	491300	0
Pip4k2a	262890	0	234960	0	589500	0	339530	314250	330060	0	0	330190
Slc7a5	2418900	3252200	1912500	2676500	0	2417500	0	0	2579000	0	2737700	0
Sphk2	839930	929660	938280	1085300	737910	1211200	0	0	0	901060	894970	0
Coro7	1443500	2192800	0	1505000	1490900	961940	1843600	2221500	2469800	5781500	1226100	1787100
Golim4	301970	0	284470	58457	485630	80120	501430	593420	655700	539680	604240	0
H1f0	1878400	2732500	3379200	5658500	0	1776900	3755700	0	5234500	4563700	3378700	2904200
Huwe1	1909700	782860	0	0	1166800	644440	1041000	1172400	998410	0	1211500	1033400
Anxa7	9427900	11948000	8151000	9822300	10692000	9211400	9055700	17734000	11263000	12046000	9092400	10138000
Bsg	4398900	5623800	4732700	4187000	2225000	5604500	4565900	6214000	5565900	3932800	0	3608700
Capn2	3198400	2876800	2154600	597100	2570600	2991100	3699300	5641900	2392800	3470500	2267400	3377900
Cybb	4687700	6874500	4268600	4715400	6008300	5097700	7681700	8138000	7129800	7271400	5604000	7183500
Myo18a	426930	418800	0	0	644050	426190	706760	876230	466500	835030	0	0
Myo1g	3405100	3057300	3644500	1969400	3490000	3743500	3999100	3827800	3163400	3819700	0	3164300
Anxa11	2679000	2163300	0	0	0	0	0	0	0	0	0	0
Tmed9	15552000	12272000	13161000	21451000	11742000	13404000	17742000	19205000	17325000	16660000	11618000	17575000
Plcg2	13740000	7359600	7842800	6387300	10515000	7403600	8381900	10935000	10377000	11572000	8178700	8293200
Coro1a	290060000	232450000	127270000	196570000	199760000	226010000	127200000	163060000	265350000	273870000	193300000	144190000
septin8	2412600	2713000	2044600	2681900	0	0	2301900	0	3549900	2313300	2087900	0
Snx18	2509400	2628600	2184200	2368100	2255400	2204900	2798100	4185700	3022800	3596400	0	2554900
Got1	25979000	40381000	21492000	35827000	22043000	27742000	15615000	21554000	35391000	34254000	18131000	21290000
Ube2d3;Ube2d2b;Ube2d2	0	0	0	0	0	0	0	0	4335100	4125800	0	3131900
Vav1	8787700	7816900	5960600	4426900	7824200	5473400	6729500	13367000	7092400	8661000	5673900	8227300
Vim	130210000	50727000	149310000	152270000	263010000	107490000	279000000	352000000	287750000	279440000	103000000	111410000
Wwp2	598930	537850	0	0	766330	600000	880810	672700	762720	845750	952870	840850
Actr2	29976000	43767000	23840000	35090000	29748000	33467000	22308000	22395000	32420000	31292000	20909000	22220000
Actr3	61846000	92805000	50536000	79067000	58168000	74536000	53351000	37447000	66476000	71771000	53025000	44328000
Arpc2	20725000	27073000	12418000	32640000	18564000	24287000	6168600	3460400	23909000	18599000	11910000	4210400
Calm1	23074000	39540000	18031000	25052000	30876000	38004000	21799000	9388200	35692000	26647000	22554000	10985000
Cops4	5845500	7523100	5537200	6346000	5229700	6486100	3062600	3139800	5762800	8094900	3899400	3927900
Actr1a	11491000	12095000	7773400	12630000	9827500	9428200	10069000	5303200	10473000	9645500	8554200	9517500
Myh9	355630000	458100000	328880000	397460000	324750000	404470000	408430000	334990000	383510000	563170000	275250000	344380000
Aimp1	16983000	16646000	11536000	16983000	16388000	15128000	11182000	11295000	15466000	14447000	14707000	12417000
Prdx6	14401000	16597000	15163000	18606000	11823000	14957000	9310700	9872300	16882000	18951000	6801700	11845000
Golph3	0	0	0	0	865640	389970	0	0	538010	462960	0	0
Pafah1b2	2631800	3004700	1926400	3674200	2425100	2703100	1330600	0	3110000	2488900	1833000	1286600
Pla2g4a	8180600	9922200	8105600	6736200	5426100	8558900	7045600	3558600	6810700	8816300	6282600	6224900
Sec23b	8534800	12470000	9876000	9662200	12957000	12988000	7260700	10438000	13135000	14211000	8276300	9620900
Dbnl	3999000	11788000	5941600	14220000	13581000	10110000	4227100	8359400	7397700	10258000	5413100	3245000
Rab21	3704900	2463500	0	3743600	4309800	0	4068700	4255400	4751700	4109100	3675500	2479700
Psme1	17219000	21551000	9140900	24242000	11338000	16252000	7619700	9263600	19911000	14779000	13962000	10800000
Tap2	2668200	3600600	2531000	0	2001400	2835400	0	0	0	1532600	0	0
Ap3d1	1162500	1164200	688980	0	1318400	1177700	1045700	1119600	1028500	1262000	1433200	1093500
Arhgef2	19825000	17412000	13765000	10874000	18181000	15393000	14388000	17711000	14582000	14423000	16885000	13231000
Tap1	3352200	3029200	2995600	2372600	2527500	2355500	2408000	2251000	2240000	2994300	3046900	1974200
Dock2	10626000	6967500	8976300	3647300	7908300	6890900	6692100	9399700	6659500	6923500	5429800	7252600
Stxbp2	4563400	3301500	4977300	3609900	5212800	3855600	4290900	5088700	4275700	5231500	4515400	4772000
Gapdh;Gm7293	1406900000	1725500000	984950000	1229900000	1140300000	1211200000	947860000	887680000	1390900000	1269300000	1161700000	936630000
Gnb2l1	116580000	99868000	92762000	75223000	106440000	88618000	98087000	80906000	104370000	103270000	115040000	97357000
Hk2	3680600	3638900	0	1856200	2637900	3046100	2576200	0	2623700	2537000	3393100	2153400
Ehd1	15452000	17264000	11919000	14249000	13252000	11559000	11425000	10703000	13524000	15589000	9707100	15315000
Nae1	1188200	1797800	0	0	1070300	1256500	0	0	1104900	1095300	1278600	1207100
Ncf4	4967200	6890900	3984900	5850300	5004500	5324600	3968200	4695700	4415900	6932400	4715300	4742400
Pfkl	6738800	7780000	2862500	3185400	3402800	2394700	1948000	1897200	7034000	4773400	2821400	0
Tubb4b	79594000	98071000	64450000	71951000	76481000	73885000	71543000	69370000	70488000	85278000	63930000	72225000
Tpp2	10541000	8812900	5479000	6551900	5887300	6440900	3835800	3739800	4775800	6524600	6009500	4438500
Ero1l	29556000	27192000	16826000	20267000	16562000	19273000	15024000	14928000	19989000	14480000	20797000	14743000
Gnai3	3856000	3015100	2116600	2626500	3783400	2161000	3105100	1821200	3725200	1947200	4469200	2339300
Lamp1	11313000	13663000	9823600	12806000	12995000	13989000	6185000	6477100	11962000	7943900	0	5952500
Mesdc2	1533900	2504200	1230000	1713200	1528400	1637000	1394400	1438800	1551900	1332900	0	1389700
Pafah1b1	8410400	6443500	2811100	0	7498700	3869400	3930900	4978000	5297600	5052800	3572100	3376900
App	0	2162500	0	1408100	0	1169700	0	0	1452500	1511500	0	0
Snx4	1796600	1204000	982850	1309600	967650	1036200	879130	995510	911320	957050	1090600	908030
Ubr4	837380	759420	760070	770840	619440	617980	0	512240	0	919890	620570	935650
Anxa3	25213000	41901000	26685000	54650000	26412000	37706000	25490000	31741000	41864000	47229000	16415000	33739000
Arpc5	9817800	11609000	7890300	19968000	15096000	18389000	10371000	9265100	18844000	19868000	8000800	11934000
Cacybp	12536000	17167000	13798000	17768000	14413000	12229000	13122000	14577000	23952000	25946000	9682700	11786000
Vps29	2507800	2655600	2812100	2999400	3455900	0	0	0	0	2846000	0	0
Cops7b	458150	560230	0	512670	0	489020	364920	470670	454880	408960	0	0
Esd	17275000	28041000	16043000	22603000	15622000	18427000	16358000	13858000	26804000	25429000	10349000	14796000
Lars	38713000	35252000	33064000	22375000	37510000	31094000	27751000	27128000	29237000	37812000	26920000	35872000
Mapk3	2465500	4422500	2267600	4330700	2869700	4109000	1797400	3297200	4607600	4664200	2111200	0
Atg7	3909800	2524700	2580800	2847300	1974600	2639100	2267000	1746800	3826400	3719700	2471100	2482200
Sqstm1	21638000	24540000	8232800	17240000	13096000	11874000	14420000	15039000	21147000	21866000	8531000	11225000
Nudc	11348000	14751000	5608900	21004000	10510000	17075000	5009700	7905600	18741000	19162000	8010400	8389200
Chmp4b	5806300	8059500	3182800	9463300	5235700	9779200	5206300	7749800	14771000	13246000	3084800	5507500
Akr1b1	26205000	43661000	23578000	35043000	29664000	35687000	24839000	27988000	41302000	40601000	18145000	26953000
Grn	0	2400500	2286600	2428200	0	2198400	3097400	2413800	0	2283900	0	0
Rac1;Rac3	4234300	9043800	3222900	7919800	4491900	7810600	4476600	3444900	8173400	6819000	2633000	3594100
Twf1	1861200	1948700	2064500	1953200	2015000	2270400	1968800	1925100	2188700	2144900	1598900	1723300
Zyx	6382600	7689100	6031500	8628300	8725100	6542700	6810100	7437700	8462000	8766000	4172200	6559000
Lilrb4	182390000	85851000	167000000	65652000	122970000	86278000	186010000	152300000	148440000	87972000	217120000	215430000
Ube2v1	4706200	8211200	6380000	3797400	3317900	3382900	6526400	11298000	4447300	4964200	3751100	8198500
C3ar1	3843900	2312300	3067400	0	2448900	2392300	5393000	3184000	2717500	2063800	4796100	2580300
Anpep	2399500	1970700	3111000	4411300	3077600	2457000	5704500	3764000	3175100	2019800	2715200	1812700
Cd28	4778100	0	7300000	0	4659400	0	15409000	10986000	2512600	2208500	12544000	5324000
Cd44	45784000	36075000	70653000	25622000	50693000	30436000	89153000	79257000	29681000	20419000	77862000	39848000
Cd72	31111000	18321000	49949000	9747400	25692000	15769000	60925000	95367000	34350000	28215000	43115000	72792000
Clec12a	4782700	2168400	9996100	1572800	4786900	3366900	8978000	7448100	2887900	1380500	12569000	7619200
Ncf1	3370600	1688800	3479300	3177200	3223600	2249600	3379300	4482700	3101300	3187000	2877600	3256200
Derl1	2186100	2649900	2749100	2174600	2927900	2324700	3488500	2704800	2095800	2777900	3464000	2957600
Fcgr1	40868000	21258000	34165000	15379000	32790000	25550000	58188000	61527000	26662000	14062000	49778000	53695000
Fcgr2;Fcgr2b	71470000	38623000	68049000	47908000	55487000	46529000	88875000	110640000	58118000	52119000	91780000	102620000
Igsf8	136370000	76787000	124740000	44107000	85975000	46563000	184610000	189250000	65917000	28824000	190720000	195040000
Irgm1	3365500	1928800	4294600	1823700	3417500	3023500	4021900	3502000	2824500	0	3858100	3337800
Itga4	5230800	2325400	9997300	4839900	5166300	2415500	8826000	10572000	2768000	3074500	6527500	6278500
Itgal	3228500	1362500	1396000	1123800	2052900	1007300	1862000	3322000	1457100	1072300	2113000	1595400
Itgam	43038000	56476000	52780000	61296000	41790000	46100000	55507000	69961000	39885000	44149000	59520000	47062000
Itgb1	75963000	102280000	75625000	82159000	49782000	79687000	85548000	182360000	54016000	68334000	93713000	135440000
Itgb2	57243000	56276000	56984000	52725000	54134000	41857000	66532000	79691000	44496000	46274000	67340000	55714000
Krt1	976440	0	2194900	0	1853900	0	1318300	7942800	0	1830100	1595800	0
Lphn2	5722800	1809000	5113400	1474800	2523900	2349800	5954800	6641700	2030100	1625400	6359800	2792400
Notch2	10051000	4606700	11924000	4154600	6834500	4144300	13229000	14164000	5924100	3829300	12287000	13276000
Nrp2	19923000	10365000	21444000	6811800	16219000	8815600	24816000	34155000	16094000	12745000	26291000	35802000
Pi4k2a	2030000	1606500	2474100	2307500	2142900	1857600	2831800	3528400	1490700	1657700	1837000	2762600
Plxna1	24027000	16520000	22890000	13040000	18625000	13403000	29045000	24737000	15302000	16783000	30047000	24371000
Plxnb2	262640000	168070000	340790000	137130000	243040000	169390000	421710000	404130000	185250000	141690000	416360000	342440000
Rftn1	6245300	6289600	3072300	5441200	7516400	6757500	6558900	8358200	5126500	4142700	7014400	4927500
Ptprc	69558000	68778000	70984000	67508000	68501000	55291000	77007000	81654000	45699000	56163000	92853000	67012000
Pvr	2117900	1812000	2546500	2145700	2137900	1511000	2860500	2974200	1863100	1612000	2404800	2389200
Tlr2	9307400	3468300	12720000	4059300	7334500	4573100	11248000	14021000	6459400	4734900	12738000	12106000
Atp1a1	205840000	170920000	230180000	174350000	195680000	164580000	248970000	264830000	176980000	153670000	226190000	229270000
Itga5	352110000	158170000	315490000	111490000	192730000	146390000	383550000	400070000	226380000	151590000	376720000	414930000
Nrp1	154960000	74785000	205610000	71719000	125470000	85004000	213050000	215540000	94196000	66175000	255900000	249340000
Vps26a	3278800	4662200	3050400	5536100	3431300	4091300	5402400	0	3119900	3121300	2600700	5320400
Dctn1	3545900	3467600	0	2444700	3136900	2573500	0	3938400	3385400	2522200	2745200	3893200

Mitochondria

Genenames	LFQintensityA1	LFQintensityA2	LFQintensityB1	LFQintensityB2	LFQintensityC1	LFQintensityC2	LFQintensityE1	LFQintensityD2	LFQintensityD1	LFQintensityE2	LFQintensityF1	LFQintensityF2
Bak1	4812000	4963200	8871900	7047500	6354600	6067400	4891200	0	2749600	4661800	5419300	5455300
Tnpo3	2817900	2958800	2263900	1858600	1516600	1811000	1045900	0	1220900	1915400	3022000	2129700
Glyr1	607190	522220	528650	543850	754850	424990	424050	0	237360	0	879300	334470
Lap3	1454200	1936300	1968300	2238200	1573900	1822600	1334300	1718300	1739400	2119100	2257500	1781200
Lonp1	49105000	46277000	47403000	40911000	43864000	43796000	40824000	39843000	31630000	35722000	42331000	40475000
Mrpl10	1811900	1889800	1088000	1497800	1247800	1815400	931990	0	723140	1228900	2257500	894840
Mtx2	1337500	1913000	1420000	2709100	1217200	2040500	675210	0	1104600	968460	1920800	0
Pmpca	1184300	461220	1143200	0	2095000	649770	602500	0	380360	505110	1280100	303610
Pold1	2401400	2957000	4407600	2572400	2129200	1926300	3150200	946910	1646000	2968500	2160100	3708200
Ptrh2	3188100	4313400	3697900	5413900	3052300	5065800	2059600	840600	2725200	2006300	4241900	2161500
Sccpdh	788860	959830	550180	969170	738330	941340	541000	0	409070	532540	841460	0
Tcirg1	0	4029900	2806800	4617600	0	2863600	0	0	0	0	3486400	3399600
Acadsb	5428900	2418600	3943100	3503200	4287000	3716700	5979000	2430600	2327400	3252000	3980400	2593300
Acox3	2756600	2228600	1985300	0	2281300	1786200	1906900	2445800	1434600	1292800	2759100	1753500
Dpm1	1050600	948720	892610	1105600	1035100	895940	838260	0	791010	512060	3212100	792500
Pgrmc1	0	2554400	2104800	3043100	2042600	3232500	3119200	0	0	2042800	1746100	2966300
Comtd1	2318200	2288100	2545800	2371300	1945400	2547900	2468500	1326000	1522400	1764400	0	2848300
Dock10	3957600	5024100	6567100	3954300	6413900	5392300	5954400	6654200	3679300	3667000	5585200	4760300
Mcl1	388740	458550	574840	820770	731760	764760	624500	0	489660	529460	1037000	760170
Mrps31	1839800	1799900	2395900	1993500	1822600	2135400	1865200	1140100	1762300	1667900	3367400	3001200
Ndufa10	2358700	2107000	2303800	2550400	2182100	2124400	1679600	0	2158700	1285300	4820800	2265100
Tmem14c	999960	2333000	1106400	2466500	1219200	1375800	943680	1401300	893580	428380	2603100	0
Uqcrq	1846700	0	0	3577200	2429200	2422700	2920000	3408600	2398400	2593500	0	3204000
Abcb7	2749700	2154700	3979700	2611500	1891600	2404300	1918200	0	1868800	2012700	2510900	1722800
Ak3	1312800	1409300	0	4116600	0	1392300	1069100	675300	985040	991790	1168300	0
Mgst3	2329200	2785500	4510000	4024600	2868400	3716300	1424500	570030	2250700	1828500	3370100	1678400
Xdh	4282300	5149500	4640800	7009800	3578100	3871900	1813200	1349900	3102600	1757800	3509400	1074600
Fyn	0	333360	977580	758830	661590	719230	668230	0	0	398280	0	0
Glb1	1213400	2198700	1020000	3627700	1505100	1778200	604110	0	0	570420	1705500	640000
Gtf3c4	295650	248820	453630	345170	342360	365720	260580	0	0	0	0	0
Ivd	999950	1665500	0	1880500	0	1412600	0	0	0	0	0	0
Mat2b	0	0	2020300	3205900	1887800	2153000	0	0	1485900	1669100	1627300	1613400
Mecr	1836800	1550000	0	3233400	0	0	0	0	0	0	0	0
Mrpl28	677610	1371000	1170000	1975600	1609500	990760	0	0	0	0	0	0
Nt5c	1708700	1948900	0	5627200	1589300	1778500	0	0	1437000	0	0	0
Timm17a	2132000	3131000	0	2880100	2562400	0	2114000	2273900	0	0	0	0
Uqcrb	2004800	4112900	2125700	4916400	2119900	1890400	2137900	0	2087100	0	0	1914200
Ciapin1	2157900	6050100	1889700	5425200	1823300	3558900	1792900	1529300	1582500	3724600	1881400	1450400
Cse1l	27700000	27867000	20577000	32612000	19728000	20224000	14555000	19157000	20790000	21892000	19944000	11775000
Ddx1	9835200	11420000	16463000	11643000	9815400	10466000	7459700	5527600	9127300	11683000	11608000	8161300
Dnm1l	3890600	3125600	4508300	3276400	2653300	2691000	1939800	0	2854600	4216000	3208900	0
Park7	2728100	5488000	4199200	6036800	3750400	4729000	1748300	4784400	4486100	3798600	1964300	3397200
Ndufv2	1972900	2677900	0	2784000	0	0	0	0	0	2076100	0	0
Nme1	5303200	8553700	4915500	9578400	4682700	3343700	4435300	0	4279900	7382800	5097300	3643700
Pmpcb	0	1326600	0	1912100	0	1164000	0	0	702060	1148000	0	0
Ube2l3	7468800	13854000	5528700	15918000	7733700	9686100	4633800	4237000	8077100	7265300	6456700	4267300
Snrpd1	9642300	10284000	6837500	22530000	8809300	9132500	11062000	1899100	9799100	9220000	5629100	6198100
Spr	2322400	4814200	0	5550800	3114100	4142500	2475000	0	4344000	4795900	2089500	3226100
Abcc3	0	763510	0	721010	851570	0	766240	0	743180	0	827100	663550
Atp6v1d	1838000	2270000	2227200	4042500	2622800	2436000	0	0	2308200	2512100	2047600	3317100
Clpx	809770	813080	862940	723060	848270	470050	0	0	1023400	723630	976010	0
Coasy	1605500	1655700	0	1487900	1442500	1773000	0	0	1539000	1205400	1233400	907940
Cyb5r1	9185400	14855000	10937000	15433000	13176000	17404000	10497000	11196000	14311000	12579000	12453000	14541000
Ddx6	6751700	6918000	6008400	7547500	7156400	7942200	5348200	7304500	6711400	5385400	6061100	5270000
Dnmt3a	1436000	1182300	1341900	1220500	1554800	1546900	1767100	1036600	1371900	1326000	1403000	1309600
Asl	3901000	2062100	2435000	3172800	2506300	2273300	1231000	1384800	2032400	2793000	2018900	3030500
Gpd2	0	1274900	1149300	1059200	939810	1027700	874420	921580	1019800	834020	889560	908790
Hsd17b11	2333000	3203900	2323300	3339200	1807500	2422500	0	0	1769100	1669300	4022600	2247400
Idh3g	5387100	8454100	4699200	9347100	7764700	7951000	3663500	4131300	6014000	4789100	8393800	4656300
Tomm40	7359800	11230000	7638100	6720900	7964900	11115000	6738600	4027000	8467200	7542200	8012100	6852000
Mrpl46	967250	2014100	1283600	1838500	934250	1445600	0	0	935860	1503100	0	1503600
Pck2	30827000	30171000	27114000	29709000	31550000	29624000	27168000	22165000	25276000	24969000	26910000	25938000
Qars	7522000	8090200	5793100	5284900	7804100	7471600	4749600	2109400	6076200	6297000	7887300	3855500
Rnh1	58983000	64699000	55225000	62713000	76254000	62318000	59957000	47529000	63228000	56981000	63153000	52817000
Sphk2	839930	929660	938280	1085300	737910	1211200	0	0	0	901060	894970	0
Tsfm	2320800	2833300	2544500	2820500	0	2878000	2499200	0	2629000	2319600	0	2457600
Uqcrfs1	2020200	2477800	1760400	2996200	1851500	2532700	1752000	0	2307500	2018200	2411000	2227500
Apool	4529800	3535200	3456600	3036800	4800600	3816800	4566700	5035200	4716900	3444400	4619600	0
Dhfr	2986400	2356800	0	0	0	3105900	0	2605000	4030200	2518600	0	0
H1f0	1878400	2732500	3379200	5658500	0	1776900	3755700	0	5234500	4563700	3378700	2904200
Mpst	0	2581400	2677700	2257900	0	2528200	3679400	0	2915600	2879300	0	2417800
Mrpl40	313730	397220	304470	655220	405620	422790	575110	883300	0	933470	320360	519090
Cybb	4687700	6874500	4268600	4715400	6008300	5097700	7681700	8138000	7129800	7271400	5604000	7183500
Fads1	0	2559700	0	1954500	0	2613100	3415600	3216200	2881400	0	2596300	3181900
Nfkb1	0	1376300	0	1154700	0	896800	1445400	0	1224100	2719800	0	2431600
Nop56	2833100	2521500	3361000	2895100	2676900	2221000	4289300	2407400	3361300	2552400	2246200	2174500
Pak2	3184200	3543200	3409500	4008000	4237200	4788000	4443900	6981100	4583100	4601800	2473700	3794500
Parl	1350300	1082900	0	0	1539300	1126200	1674900	0	1312100	971120	1519600	1177700
Prkaca	9669000	8424300	9402600	8056500	9418900	8381600	10510000	15775000	9911100	9781300	9754300	9169300
Prr11	1888500	0	1816800	0	2218800	1728900	2748500	3589900	3060800	0	2404400	3316200
Rps27	11230000	15614000	4621600	14485000	11135000	15140000	10796000	18040000	17257000	14847000	10676000	8417400
Rps27l	14458000	14474000	9777300	9643100	13608000	13575000	15804000	17410000	15669000	16457000	18350000	12425000
Sh3bgrl3	2685800	6846200	3534200	8456200	2379800	4403000	6246300	8432100	4707700	7890400	1237900	7513300
Slk	888450	925150	394380	1021200	1168500	731240	814570	1490800	1364400	1200000	921770	557040
Snrpg	1792300	1147100	0	1305900	2236500	1716700	2128300	2188500	2956600	3200400	0	1981600
Wwp2	598930	537850	0	0	766330	600000	880810	672700	762720	845750	952870	840850
Yars	6464000	5529200	3751300	5423600	5612300	4893900	6484200	7278200	7392700	6281400	4069500	4826700
Aldoc	8755700	15353000	7389600	14403000	10032000	11087000	7748100	8197500	13305000	12367000	4930500	9403000
Bckdha	723050	1119900	1053200	882980	0	1709500	0	0	1051700	1215600	0	0
Cdk18	3304800	2502700	3408500	1255300	2384300	1514900	0	714990	1723800	2683500	1562300	1252700
Ckb	65898000	104210000	57986000	113000000	62261000	84798000	46840000	35939000	82531000	96744000	47489000	63618000
Cth	9593100	16696000	10320000	15721000	10064000	13953000	5067700	4747700	13381000	16272000	6864200	8064700
Glod4	1289700	4982700	1225600	3779600	1975200	3113700	1520400	1750800	3129700	2811900	1279100	1370200
Hk3	23565000	22277000	21933000	17809000	24583000	22358000	18836000	22096000	19841000	23282000	18748000	19181000
Impa1	3222400	7175200	2208100	7077500	4320800	5680500	2284300	2889400	3902500	3541600	0	2127100
Nars	29369000	28961000	23412000	24707000	27105000	26688000	23292000	19121000	28029000	26799000	21046000	20830000
Pnp	2624200	3087600	2075700	3048500	2792400	2765000	0	2195000	2954500	2488600	1927700	2757000
Sars	46660000	65309000	33584000	58289000	34295000	38846000	27225000	21662000	49692000	49528000	35734000	25383000
Stat3	2961300	0	1634300	0	1550400	2157100	0	0	1240200	0	0	0
Psmb3	5985700	8780300	3347700	9557900	6653400	7414700	2970000	2781600	5394600	5917200	5058800	2838000
Aars	29064000	29817000	22248000	25620000	22578000	26674000	19692000	24138000	22620000	29688000	16672000	22601000
Aldh9a1	11638000	17019000	9782500	11380000	14560000	11599000	12467000	9268000	15815000	17551000	8813400	12873000
Asns	15055000	12244000	5805500	8334700	9848800	8241100	8216900	9343400	11422000	10829000	13648000	8650500
Ddx39a	6708200	4729600	4356500	8146100	3921600	2656600	6279500	1267700	3549700	9940800	6475600	3889600
Dlat	17050000	15947000	9705800	13287000	15065000	15740000	11254000	13303000	14921000	12646000	14178000	12274000
Dpp3	0	5554800	3963700	3963200	5157200	3822000	4219900	3261700	5517700	5644700	3440000	2898200
Drg2	612800	541100	0	471480	520590	478270	411160	542640	517820	527580	605250	478460
Eif3e	28989000	20165000	27089000	17857000	25212000	19145000	22598000	23495000	27235000	24780000	24142000	23030000
Hk1	15283000	14549000	13715000	14491000	12710000	13179000	12321000	14731000	13908000	14953000	13845000	14011000
Iars	12510000	8401400	9428500	3790400	10372000	7271500	8533900	5356600	9196500	10298000	9486100	10298000
Ldha	443650000	581720000	275700000	404340000	319840000	321440000	266840000	324990000	410070000	425770000	322300000	320820000
Nae1	1188200	1797800	0	0	0	1070300	1256500	0	1104900	1095300	1278600	1207100
Rars	46291000	43209000	42485000	28473000	44177000	36225000	39483000	40454000	39704000	41520000	37279000	39724000
Tomm70a	4315300	2107500	0	2101000	2317200	2066600	2316200	0	2544400	2389300	2312300	2138000
Ubr4	837380	759420	760070	770840	619440	617980	0	512240	0	919890	620570	935650
Abce1	10195000	9539800	9938200	11188000	9816400	8768400	6181400	10665000	13012000	12700000	9864700	7760100
Acy1	1043700	0	1085800	1325600	979590	1021800	987900	528940	1047600	1911200	811960	978740
Akr1b8	12463000	22000000	9212500	17531000	11151000	15128000	11375000	15745000	16770000	19362000	6151100	12074000
Cab39	4329900	5904400	2899900	6345500	2844500	3350300	2740800	3776800	7139600	4329600	0	3018500
Cops7b	458150	560230	0	512670	0	489020	364920	470670	454880	408960	0	0
Dcps	2929900	3174100	0	4689100	2730200	3348200	2886800	3059300	3302300	3004700	0	0
Mars	17475000	17076000	17146000	10671000	13550000	14282000	11469000	20223000	16061000	16141000	10485000	13120000
Mdh1	24468000	40672000	23719000	39775000	28533000	31709000	28974000	27125000	35095000	41009000	25570000	26296000
Myef2	8091800	4862400	6518800	6279100	5067900	5351400	7560100	6436000	7419300	9917900	4012000	3670600
Myg1	5609300	5488900	5161300	6933700	6488600	5135500	4940200	4667900	6986000	7163900	3385700	5258400
Prdx1	158220000	254990000	156320000	236190000	170930000	188330000	178220000	162520000	215630000	239890000	98344000	176820000
Atp5d	5730600	1997900	2101900	7080500	8848800	2024700	3284000	3492900	10786000	5026400	6482100	2769100
Bax	4377300	6368600	5084600	7825000	4830700	6301500	3560300	4515600	6763300	6322100	3501500	3986300
Grn	0	2400500	2286600	2428200	0	2198400	3097400	2413800	0	2283900	0	0
Srsf7	4565000	6113300	6500700	12721000	4785200	6070400	7083600	5321500	7633400	9928800	0	3270900
Txndc17	716120	1836300	651990	1872900	892370	1214200	657120	753340	1728800	1799300	0	675470
Dlst	21231000	19712000	16566000	16290000	17137000	18733000	23492000	27440000	19227000	16208000	21709000	24602000
Hat1	0	0	0	0	0	0	786660	597100	0	0	0	786550
Mrpl43	4036700	2425100	4470200	2170900	4243400	4262000	12982000	8926900	4014400	3958400	3832900	15396000
Pccb	135990000	98786000	115350000	70025000	111890000	92848000	112120000	260030000	45991000	52133000	136350000	190050000
Rpl37	0	0	0	0	0	0	1451300	6785400	0	360420	0	1699300
St14	2865800	2171300	4895800	2052200	2043700	1775000	5986100	3711900	1958100	1664900	3358400	2519300
Nrp1	154960000	74785000	205610000	71719000	125470000	85004000	213050000	215540000	94196000	66175000	255900000	249340000
Vat1	1826300	2919700	4110700	4921700	2357200	5885300	2470700	2554500	2531900	2384300	2602800	2840300

Phagsome

Genenames	LFQintensityA1	LFQintensityA2	LFQintensityB1	LFQintensityB2	LFQintensityC1	LFQintensityC2	LFQintensityE1	LFQintensityD2	LFQintensityD1	LFQintensityE2	LFQintensityF1	LFQintensityF2
Ecm29;AI314180	2879500	2017900	3739900	1854400	2424600	2030000	2874500	1338100	2320500	2651200	3059700	2595300
Lmbrd1	0	0	1771900	5692300	1971100	3764000	0	0	0	0	0	2213700
Npc1	1806200	4094400	5476600	4275500	3054600	4902100	4583600	1801100	1502000	2399000	2288800	4163100
Sec61a1	5453900	8019000	7606300	4551300	6821900	6069100	3808200	1761600	2645900	3510500	8078700	2579100
Sigmar1	4578700	2354100	4425400	2746800	3208200	2224200	3023700	1683300	2819800	2589100	3399100	2781100
Tlr7	4620900	3832600	3833000	0	3973900	2588900	3314100	3302100	3192200	2549500	5081000	3046100
Btk	3999800	3556900	3541000	3073900	2802500	2867900	2813900	2119100	1723500	1977000	3609700	2478700
Gpnmb	8067800	6282700	10862000	13876000	12106000	17295000	5013000	2807500	5956500	3814700	7615200	5293400
Cltc	337320000	263460000	307880000	142410000	350650000	250570000	252060000	225510000	219680000	223900000	344470000	268440000
Sh3gl1;Sh3gl2	1410000	2071200	1065000	2225300	0	0	722520	0	0	0	1092100	0
Rraga;Rragb	2526100	2387300	2344100	3171200	2084000	3250200	2050200	0	1287300	1655000	2231200	2578000
Fcgr3	2691400	0	13818000	0	0	0	0	0	0	0	8202200	10093000
Tcirg1	0	4029900	2806800	4617600	0	2863600	0	0	0	0	3486400	3399600
H2-D1	247470000	171050000	183720000	139460000	176280000	133870000	178310000	192490000	132440000	93152000	223630000	133140000
Atp6v0a1	2233200	1783200	2530200	1944900	2829200	2348600	2664600	2325700	2080400	0	3899000	2364400
Cdipt	3253100	3182400	3402600	3815000	3589100	3703700	3864900	2657100	3272200	2013100	5665800	3672700
H2-K1	33131000	31238000	29815000	32153000	24174000	29010000	35687000	25721000	26458000	26083000	35321000	34761000
Gak	0	106360	0	0	96880	135260	106800	0	56836	0	219040	0
Pgrmc1	0	2554400	2104800	3043100	2042600	3232500	3119200	0	0	2042800	1746100	2966300
Scfd1	3766300	3455600	4940900	2958700	5264600	4644800	5211000	4057800	3538800	3344300	3447300	3411400
Dync1li1	2059600	2069100	2209300	2498700	2390000	2149800	2411700	2697700	1521600	2406400	2785300	3046900
Neo1	7006700	2820800	17118000	5285000	7624900	3792900	12459000	9662900	1801800	2329400	13791000	11717000
Tm9sf4	1428900	1812800	1586000	1503100	1548700	1506600	1600200	0	1359800	0	1529300	0
Apobr	1406300	3740900	1890500	6167200	0	3285500	2311600	2201900	1769100	3196900	1733500	3070600
Arl1	1376900	3433800	1603700	2757800	1405100	0	1365700	0	1028300	0	0	1089800
Atp6v0c	5003300	7807700	6948700	11104000	6832100	10306000	5136400	6289000	6100000	6907600	6888700	5157900
Arf5	3451400	3100700	0	3237200	0	0	3387000	2845700	2883500	0	0	0
Snap23	0	0	0	1619100	1519500	1550500	0	0	0	0	0	0
Srpr	4377500	2565800	3208800	4512200	2921600	2305200	1417500	3169300	1695200	0	2199300	2529200
Stx16	205120	0	0	840320	0	512250	0	0	87848	86104	182680	0
Tmed5	0	1563200	0	5279600	803560	1292500	0	0	0	0	0	0
Fyn	0	333360	977580	758830	661590	719230	668230	0	0	398280	0	0
Glb1	1213400	2198700	1020000	3627700	1505100	1778200	604110	0	0	570420	1705500	640000
Lamtor1	1363800	0	0	4132100	1656500	1715400	1383000	0	1324900	1432200	1423300	1504800
Serpinb6;Serpinb6a	14681000	12073000	15041000	15016000	14945000	13380000	11706000	10785000	11825000	14248000	13322000	12589000
Slc4a7	1337900	1477400	963370	1742700	970950	943410	1617600	970540	730750	965910	971300	833760
Snx12	869700	1980100	0	2239900	835630	1122400	751440	601940	1069000	1074400	0	834970
Tsnax	441620	975270	387210	1696500	557790	1216700	455700	0	264540	355570	0	0
Cops2	1967100	3069800	1803400	3642100	1697600	3403700	1639300	1357200	3040400	1582200	0	1129400
Ap1b1	8705000	9064100	10646000	11174000	8279400	9118600	6497800	3233300	7441200	9881900	7333900	9535900
Dnm1l	3890600	3125600	4508300	3276400	2653300	2691000	1939800	0	2854600	4216000	3208900	0
Anxa5	170480000	308090000	187920000	331330000	174600000	228950000	154800000	126090000	205750000	256070000	126400000	188400000
Osbp	931830	1104600	960400	2152400	1076700	1113200	0	0	1159700	0	0	0
Park7	2728100	5488000	4199200	6036800	3750400	4729000	1748300	4784400	4486100	3798600	1964300	3397200
Rhoa	1780900	5543800	1586100	5352600	2441000	4192200	1642700	2524500	2757600	3097600	2922500	2148900
Gabarap;Gabarapl1	0	0	0	1212100	0	1083100	0	0	1047600	758020	0	0
Otub1	5271100	4607200	4635600	7184300	3453700	3968100	2329600	2420900	3893900	5571700	2639900	2862700
Ppt1	0	6352000	4313300	7494500	4248200	3979600	3325400	2942200	4255700	5091200	0	3763500
Ube2l3	7468800	13854000	5528700	15918000	7733700	9686100	4633800	4237000	8077100	7265300	6456700	4267300
Rragc;Rragd	1697300	3817300	1837600	6193900	2085300	2326500	0	0	3337300	2534000	2309600	0
Smap;1110004F10Rik	0	3437800	0	6294400	0	2745000	3034100	2926400	3756400	4417300	0	0
Twf2	2167300	3813200	2133300	3977000	2205000	2361400	1944600	1456000	2051900	2619900	1931600	1882600
Rab35	0	1443600	1922300	1789300	0	1328000	0	0	1489800	1430800	0	0
Vav3	0	0	1507800	0	1457000	1363700	0	0	1233000	0	0	0
Arhgef1	2818500	2573700	2150600	2224900	3357500	2468100	2059700	2126600	2566600	2314100	2130900	0
Atp6v1c1	9216000	7944700	6313200	9451200	10217000	8635900	7403400	7978500	7312800	6432500	9535000	6796000
Atp6v1d	1838000	2270000	2227200	4042500	2622800	2436000	0	0	2308200	2512100	2047600	3317100
Clint1	7429900	10135000	6341000	10858000	9370000	11464000	5930200	6944500	7558700	6088700	5774400	4900600
Arf1;Arf3	38157000	46476000	28263000	52977000	34178000	39371000	17060000	14337000	28387000	24101000	49669000	17520000
Ccdc115	0	2432000	1650100	2575700	1751000	2014000	1598000	1369600	1629600	1798600	1745400	1756800
Copg2	64269000	64452000	65491000	53015000	50822000	61088000	31171000	45801000	44083000	47688000	56491000	40133000
Hip1	0	0	1284300	994040	1321900	0	0	0	989450	935460	1125100	0
Lman2	0	0	0	2716000	2111100	0	0	0	1863500	0	1985900	2086700
Rab3gap1	1288900	523700	1377300	1283500	1163800	1375100	0	0	1281000	1211800	1077400	0
Unc93b1	4873200	6184800	5563200	4755400	6240100	5183700	4112400	4786800	5642400	3460500	5303300	4078900
Pip4k2a	262890	0	234960	0	589500	0	339530	314250	330060	0	0	330190
Slc7a5	2418900	3252200	1912500	2676500	0	2417500	0	0	2579000	0	2737700	0
Sphk2	839930	929660	938280	1085300	737910	1211200	0	0	0	901060	894970	0
Coro7	1443500	2192800	0	1505000	1490900	961940	1843600	2221500	2469800	5781500	1226100	1787100
Golim4	301970	0	284470	58457	485630	80120	501430	593420	655700	539680	604240	0
H1f0	1878400	2732500	3379200	5658500	0	1776900	3755700	0	5234500	4563700	3378700	2904200
Huwe1	1909700	782860	0	0	1166800	644440	1041000	1172400	998410	0	1211500	1033400
Anxa7	9427900	11948000	8151000	9822300	10692000	9211400	9055700	17734000	11263000	12046000	9092400	10138000
Bsg	4398900	5623800	4732700	4187000	2225000	5604500	4565900	6214000	5565900	3932800	0	3608700
Capn2	3198400	2876800	2154600	597100	2570600	2991100	3699300	5641900	2392800	3470500	2267400	3377900
Cybb	4687700	6874500	4268600	4715400	6008300	5097700	7681700	8138000	7129800	7271400	5604000	7183500
Myo18a	426930	418800	0	0	644050	426190	706760	876230	466500	835030	0	0
Rab21	3704900	2463500	0	3743600	4309800	0	4068700	4255400	4751700	4109100	3675500	2479700
Tmed9	15552000	12272000	13161000	21451000	11742000	13404000	17742000	19205000	17325000	16660000	11618000	17575000
Plcg2	13740000	7359600	7842800	6387300	10515000	7403600	8381900	10935000	10377000	11572000	8178700	8293200
Ap3d1	1162500	1164200	688980	0	1318400	1177700	1045700	1119600	1028500	1262000	1433200	1093500
Ube2d3;Ube2d2b;Ube2d2	0	0	0	0	0	0	0	0	4335100	4125800	0	3131900
Vav1	8787700	7816900	5960600	4426900	7824200	5473400	6729500	13367000	7092400	8661000	5673900	8227300
Vim	130210000	50727000	149310000	152270000	263010000	107490000	279000000	352000000	287750000	279440000	103000000	111410000
Wwp2	598930	537850	0	0	766330	600000	880810	672700	762720	845750	952870	840850
Arpc2	20725000	27073000	12418000	32640000	18564000	24287000	6168600	3460400	23909000	18599000	11910000	4210400
Calm1	23074000	39540000	18031000	25052000	30876000	38004000	21799000	9388200	35692000	26647000	22554000	10985000
Cops4	5845500	7523100	5537200	6346000	5229700	6486100	3062600	3139800	5762800	8094900	3899400	3927900
Actr1a	11491000	12095000	7773400	12630000	9827500	9428200	10069000	5303200	10473000	9645500	8554200	9517500
Lamp1	11313000	13663000	9823600	12806000	12995000	13989000	6185000	6477100	11962000	7943900	0	5952500
Aimp1	16983000	16646000	11536000	16983000	16388000	15128000	11182000	11295000	15466000	14447000	14707000	12417000
App	0	2162500	0	1408100	0	1169700	0	0	1452500	1511500	0	0
Golph3	0	0	0	0	865640	389970	0	0	538010	462960	0	0
Pafah1b2	2631800	3004700	1926400	3674200	2425100	2703100	1330600	0	3110000	2488900	1833000	1286600
Pla2g4a	8180600	9922200	8105600	6736200	5426100	8558900	7045600	3558600	6810700	8816300	6282600	6224900
Sec23b	8534800	12470000	9876000	9662200	12957000	12988000	7260700	10438000	13135000	14211000	8276300	9620900
Dbnl	3999000	11788000	5941600	14220000	13581000	10110000	4227100	8359400	7397700	10258000	5413100	3245000
Vps29	2507800	2655600	2812100	2999400	3455900	0	0	0	0	2846000	0	0
Psme1	17219000	21551000	9140900	24242000	11338000	16252000	7619700	9263600	19911000	14779000	13962000	10800000
Arhgef2	19825000	17412000	13765000	10874000	18181000	15393000	14388000	17711000	14582000	14423000	16885000	13231000
Tpp2	10541000	8812900	5479000	6551900	5887300	6440900	3835800	3739800	4775800	6524600	6009500	4438500
Ero1l	29556000	27192000	16826000	20267000	16562000	19273000	15024000	14928000	19989000	14480000	20797000	14743000
Gnai3	3856000	3015100	2116600	2626500	3783400	2161000	3105100	1821200	3725200	1947200	4469200	2339300
Lars	38713000	35252000	33064000	22375000	37510000	31094000	27751000	27128000	29237000	37812000	26920000	35872000
Pafah1b1	8410400	6443500	2811100	0	7498700	3869400	3930900	4978000	5297600	5052800	3572100	3376900
Sqstm1	21638000	24540000	8232800	17240000	13096000	11874000	14420000	15039000	21147000	21866000	8531000	11225000
Snx4	1796600	1204000	982850	1309600	967650	1036200	879130	995510	911320	957050	1090600	908030
Ubr4	837380	759420	760070	770840	619440	617980	0	512240	0	919890	620570	935650
Anxa3	25213000	41901000	26685000	54650000	26412000	37706000	25490000	31741000	41864000	47229000	16415000	33739000
Arpc5	9817800	11609000	7890300	19968000	15096000	18389000	10371000	9265100	18844000	19868000	8000800	11934000
Cacybp	12536000	17167000	13798000	17768000	14413000	12229000	13122000	14577000	23952000	25946000	9682700	11786000
Chmp4b	5806300	8059500	3182800	9463300	5235700	9779200	5206300	7749800	14771000	13246000	3084800	5507500
Cops7b	458150	560230	0	512670	0	489020	364920	470670	454880	408960	0	0
Esd	17275000	28041000	16043000	22603000	15622000	18427000	16358000	13858000	26804000	25429000	10349000	14796000
Got1	25979000	40381000	21492000	35827000	22043000	27742000	15615000	21554000	35391000	34254000	18131000	21290000
Mapk3	2465500	4422500	2267600	4330700	2869700	4109000	1797400	3297200	4607600	4664200	2111200	0
Atg7	3909800	2524700	2580800	2847300	1974600	2639100	2267000	1746800	3826400	3719700	2471100	2482200
Myh9	355630000	458100000	328880000	397460000	324750000	404470000	408430000	334990000	383510000	563170000	275250000	344380000
Nudc	11348000	14751000	5608900	21004000	10510000	17075000	5009700	7905600	18741000	19162000	8010400	8389200
Prdx6	14401000	16597000	15163000	18606000	11823000	14957000	9310700	9872300	16882000	18951000	6801700	11845000
Akr1b1	26205000	43661000	23578000	35043000	29664000	35687000	24839000	27988000	41302000	40601000	18145000	26953000
Grn	0	2400500	2286600	2428200	0	2198400	3097400	2413800	0	2283900	0	0
Twf1	1861200	1948700	2064500	1953200	2015000	2270400	1968800	1925100	2188700	2144900	1598900	1723300
Zyx	6382600	7689100	6031500	8628300	8725100	6542700	6810100	7437700	8462000	8766000	4172200	6559000
Ube2v1	4706200	8211200	6380000	3797400	3317900	3382900	6526400	11298000	4447300	4964200	3751100	8198500
Anpep	2399500	1970700	3111000	4411300	3077600	2457000	5704500	3764000	3175100	2019800	2715200	1812700
Cd28	4778100	0	7300000	0	4659400	0	15409000	10986000	2512600	2208500	12544000	5324000
Cd44	45784000	36075000	70653000	25622000	50693000	30436000	89153000	79257000	29681000	20419000	77862000	39848000
Ncf1	3370600	1688800	3479300	3177200	3223600	2249600	3379300	4482700	3101300	3187000	2877600	3256200
Derl1	2186100	2649900	2749100	2174600	2927900	2324700	3488500	2704800	2095800	2777900	3464000	2957600
Fcgr2;Fcgr2b	71470000	38623000	68049000	47908000	55487000	46529000	88875000	110640000	58118000	52119000	91780000	102620000
Irgm1	3365500	1928800	4294600	1823700	3417500	3023500	4021900	3502000	2824500	0	3858100	3337800
Itga4	5230800	2325400	9997300	4839900	5166300	2415500	8826000	10572000	2768000	3074500	6527500	6278500
Itgam	43038000	56476000	52780000	61296000	41790000	46100000	55507000	69961000	39885000	44149000	59520000	47062000
Itgb2	57243000	56276000	56984000	52725000	54134000	41857000	66532000	79691000	44496000	46274000	67340000	55714000
Krt1	976440	0	2194900	0	1853900	0	1318300	7942800	0	1830100	1595800	0
Notch2	10051000	4606700	11924000	4154600	6834500	4144300	13229000	14164000	5924100	3829300	12287000	13276000
Pi4k2a	2030000	1606500	2474100	2307500	2142900	1857600	2831800	3528400	1490700	1657700	1837000	2762600
Rftn1	6245300	6289600	3072300	5441200	7516400	6757500	6558900	8358200	5126500	4142700	7014400	4927500
Ptprc	69558000	68778000	70984000	67508000	68501000	55291000	77007000	81654000	45699000	56163000	92853000	67012000
Pvr	2117900	1812000	2546500	2145700	2137900	1511000	2860500	2974200	1863100	1612000	2404800	2389200
Atp1a1	205840000	170920000	230180000	174350000	195680000	164580000	248970000	264830000	176980000	153670000	226190000	229270000
Nrp1	154960000	74785000	205610000	71719000	125470000	85004000	213050000	215540000	94196000	66175000	255900000	249340000

Enzyme

Genenames	LFQintensityA1	LFQintensityA2	LFQintensityB1	LFQintensityB2	LFQintensityC1	LFQintensityC2	LFQintensityE1	LFQintensityD2	LFQintensityD1	LFQintensityE2	LFQintensityF1	LFQintensityF2
Ccdc47	9422800	7036800	9772100	9224400	7638500	8047300	8221800	5192800	2316700	2569500	9519000	7235600
Ecm29;AI314180	2879500	2017900	3739900	1854400	2424600	2030000	2874500	1338100	2320500	2651200	3059700	2595300
Npc1	1806200	4094400	5476600	4275500	3054600	4902100	4583600	1801100	1502000	2399000	2288800	4163100
Zmpste24	905150	1716200	0	1613600	981940	2326400	1237600	0	777180	1494500	1263900	1849800
Lnpep	13881000	15995000	12495000	15410000	10237000	11145000	10637000	8799400	9352500	7282800	15438000	8248100
Cand1	4968700	4763800	5376200	4669200	4501600	4943100	4205600	1891500	3044500	4033300	7087900	4611900
Lap3	1454200	1936300	1968300	2238200	1573900	1822600	1334300	1718300	1739400	2119100	2257500	1781200
Lonp1	49105000	46277000	47403000	40911000	43864000	43796000	40824000	39843000	31630000	35722000	42331000	40475000
Cltc	337320000	263460000	307880000	142410000	350650000	250570000	252060000	225510000	219680000	223900000	344470000	268440000
Pmpca	1184300	461220	1143200	0	2095000	649770	602500	0	380360	505110	1280100	303610
Pold1	2401400	2957000	4407600	2572400	2129200	1926300	3150200	946910	1646000	2968500	2160100	3708200
Ptrh2	3188100	4313400	3697900	5413900	3052300	5065800	2059600	840600	2725200	2006300	4241900	2161500
Rraga;Rragb	2526100	2387300	2344100	3171200	2084000	3250200	2050200	0	1287300	1655000	2231200	2578000
Scpep1	6209000	11244000	6917400	15049000	9338300	10412000	6206800	2752100	6118700	5432000	10546000	6802900
Tlr13	3257900	1883800	2435800	3111900	2695300	2096500	1847400	0	1894000	1199600	3038000	1762500
Acox3	2756600	2228600	1985300	0	2281300	1786200	1906900	2445800	1434600	1292800	2759100	1753500
Il6st	1783600	2060600	0	3150000	1264000	1675700	2626100	1707000	1207100	1931400	3399800	2961300
Snrnp200	10540000	6293000	9103300	4424800	6566900	6598400	6822100	6678000	5227400	6961600	6520000	8336000
Atp6v0c	5003300	7807700	6948700	11104000	6832100	10306000	5136400	6289000	6100000	6907600	6888700	5157900
Cops7a	2909700	3236200	3104800	5734900	2612200	3554800	2115300	0	2001500	0	2293400	3454300
Stx16	205120	0	0	840320	0	512250	0	0	87848	86104	182680	0
Ubxn1	1241400	2968000	1344900	4243100	1357100	1395900	1256800	0	1400500	1287600	1075400	0
Eif3f	15525000	17932000	16303000	18311000	11822000	15103000	11247000	10392000	12739000	10687000	15957000	9384700
Xdh	4282300	5149500	4640800	7009800	3578100	3871900	1813200	1349900	3102600	1757800	3509400	1074600
Glb1	1213400	2198700	1020000	3627700	1505100	1778200	604110	0	0	570420	1705500	640000
Nt5c	1708700	1948900	0	5627200	1589300	1778500	0	0	1437000	0	0	0
Prkar2a	1256600	2034900	2717400	3761300	1162300	2520400	1099200	834150	1535800	1756600	1394700	1054200
Psmd14	7582400	11439000	8154700	14727000	8593100	12970000	7223000	6669600	7596800	8575600	6047400	6188100
Blmh	4145900	2636100	0	7134800	0	3900900	3774600	0	4869300	0	4231800	0
Gba	3174100	4659500	3984600	7737000	2884600	5693000	1985600	0	1177000	1708300	3875300	1113400
Serpinb6;Serpinb6a	14681000	12073000	15041000	15016000	14945000	13380000	11706000	10785000	11825000	14248000	13322000	12589000
Sfn	20207000	0	77773000	3794200	0	0	8360400	0	8844700	9068000	3137900	8312400
Snx9	2653500	1686500	2292300	0	1986300	1597800	0	2142400	1857800	2064000	1723400	2090900
Tsnax	441620	975270	387210	1696500	557790	1216700	455700	0	264540	355570	0	0
Acot7	2434900	2349300	0	2202000	929370	1283800	758370	1005700	1838500	1123300	1210500	972590
Cops2	1967100	3069800	1803400	3642100	1697600	3403700	1639300	1357200	3040400	1582200	0	1129400
Coro1b	2424400	5430900	0	5436600	2247000	3566200	0	0	1954400	4262000	2325200	0
Ddb1	2513900	5315900	3192900	6147500	2597000	4143200	1906000	1369900	2695200	4493300	2760800	3535600
Dnm1l	3890600	3125600	4508300	3276400	2653300	2691000	1939800	0	2854600	4216000	3208900	0
Eif5b	3867800	9434900	3368500	14530000	3707700	9258100	3122400	5994100	2127400	9273400	2763700	5164600
Osbp	931830	1104600	960400	2152400	1076700	1113200	0	0	1159700	0	0	0
Park7	2728100	5488000	4199200	6036800	3750400	4729000	1748300	4784400	4486100	3798600	1964300	3397200
Rhoa	1780900	5543800	1586100	5352600	2441000	4192200	1642700	2524500	2757600	3097600	2922500	2148900
Psmd7	13146000	16139000	15863000	20106000	13554000	16318000	9031900	8357700	11612000	15269000	12034000	12430000
Srp54;Srp54c	7492600	12873000	0	18138000	9937900	7427000	0	0	8959300	0	7205300	6981000
Gabarap;Gabarapl1	0	0	0	1212100	0	1083100	0	0	1047600	758020	0	0
Tsn	1675600	3344400	0	4985800	0	3139400	0	0	1274700	3347500	0	1514600
Hint1	26453000	39153000	14860000	43713000	16185000	24819000	18829000	24223000	18429000	29282000	9998000	26571000
Otub1	5271100	4607200	4635600	7184300	3453700	3968100	2329600	2420900	3893900	5571700	2639900	2862700
Pcnp	0	2018900	0	1863000	0	1423000	0	0	1411800	1415200	1141600	0
Pgls	13199000	15796000	10311000	17073000	10272000	12597000	7514000	7726400	11025000	12722000	11637000	9187100
Pmpcb	0	1326600	0	1912100	0	1164000	0	0	702060	1148000	0	0
Ppt1	0	6352000	4313300	7494500	4248200	3979600	3325400	2942200	4255700	5091200	0	3763500
Psmd8	6030200	9680800	4892400	10963000	5953200	8441200	4014200	4475200	7040300	6512900	4819100	4088500
Ube2l3	7468800	13854000	5528700	15918000	7733700	9686100	4633800	4237000	8077100	7265300	6456700	4267300
Rps7;Gm9493	104910000	121730000	86344000	152120000	95051000	105350000	88711000	90214000	95264000	101750000	79803000	87146000
Rragc;Rragd	1697300	3817300	1837600	6193900	2085300	2326500	0	0	3337300	2534000	2309600	0
Psmb1	7294400	11349000	7473100	14426000	7689300	10919000	7079900	3447500	8906600	10204000	6810600	8447000
Psmb8	0	3187900	0	5136500	2301000	0	0	0	2963900	3460700	0	0
Psmd1	8276100	10111000	12021000	12441000	7042600	10764000	6547200	4034000	8682900	10185000	6245700	7364800
Rpl23	39917000	49928000	28422000	32352000	40608000	38201000	35318000	42030000	35314000	36495000	42889000	36560000
Cacybp	12536000	17167000	13798000	17768000	14413000	12229000	13122000	14577000	23952000	25946000	9682700	11786000
Cops7b	458150	560230	0	512670	0	489020	364920	470670	454880	408960	0	0
Dcps	2929900	3174100	0	4689100	2730200	3348200	2886800	3059300	3302300	3004700	0	0
Dhx9	18097000	14534000	13649000	16136000	11650000	8812600	13813000	11420000	13930000	14743000	5952800	6861800
Dpp9	725330	1075200	1429400	707550	1199300	1047400	979690	638560	822200	1530500	656200	922760
Enoph1	0	0	5691800	0	5122600	3352300	0	0	0	11907000	0	0
Esd	17275000	28041000	16043000	22603000	15622000	18427000	16358000	13858000	26804000	25429000	10349000	14796000
Ppm1g	2962600	4583000	3480800	6306900	2590900	4591600	3145800	3517800	4668600	5657700	0	3087200
Prep	0	5085300	0	4307000	0	0	0	0	5842900	5216600	0	0
Prmt1	3171500	4681700	3866700	3852800	3855100	3877000	0	2762300	4379500	4940900	0	3549400
Ide	1021100	1175900	805720	1023200	963530	1101500	1012000	0	1089500	1333600	0	0
Uba1	14574000	19711000	15362000	17272000	12120000	17123000	10973000	10905000	17651000	21181000	9702300	13213000
Ube2v1	4706200	8211200	6380000	3797400	3317900	3382900	6526400	11298000	4447300	4964200	3751100	8198500
Apobec1	10019000	8931400	13728000	9212800	16959000	13636000	18632000	16942000	9233100	11989000	12330000	11811000
Ddx3y	5389600	2402500	4363500	1787100	5762900	2389700	4949900	0	3364200	3364200	4237400	5632300
Nup155	5228700	3797700	5887100	3612100	5187700	5295100	7596800	5023500	4694500	4379300	8313500	5736900
Rpl37	0	0	0	0	0	0	1451300	6785400	0	360420	0	1699300
Serpinc1	2394000	2354300	2445500	1929000	3194400	3683600	3181500	2705500	3140200	1232800	3373300	3377700
St14	2865800	2171300	4895800	2052200	2043700	1775000	5986100	3711900	1958100	1664900	3358400	2519300
Gnl3	833810	0	0	0	0	849720	1834400	0	842070	2583300	1082100	0
Pepd	2995900	4809600	2515700	4433500	4059100	3561300	1516400	1399300	5642000	2202400	2309600	1595500
Psmc6	28442000	29462000	22199000	26743000	23939000	25739000	18875000	16862000	25893000	26541000	25252000	21081000
Psme1	17219000	21551000	9140900	24242000	11338000	16252000	7619700	9263600	19911000	14779000	13962000	10800000
Gars	63891000	63364000	41230000	48125000	51167000	46137000	41514000	50847000	56004000	53387000	48075000	40282000
Gart	7241100	6830800	4950100	3388000	4299200	4599400	4084900	4322400	5806300	6194200	4255900	3702600
Ppa1	0	3376100	0	0	1225100	1581000	0	0	1499600	1670600	0	0
Chmp4b	5806300	8059500	3182800	9463300	5235700	9779200	5206300	7749800	14771000	13246000	3084800	5507500
Dnase2a;Dnase2	1430300	1594300	0	1693400	1415300	1447000	1382600	0	1173700	0	0	0
Prdx6	14401000	16597000	15163000	18606000	11823000	14957000	9310700	9872300	16882000	18951000	6801700	11845000
Grn	0	2400500	2286600	2428200	0	2198400	3097400	2413800	0	2283900	0	0
Nploc4	0	1699100	0	2283600	1799500	1373900	966740	0	0	3176400	1317300	0
Pebp1	7808900	10868000	5982900	13554000	9442800	11430000	4993400	8819100	14057000	12616000	4830400	5964700
Sgta	0	0	0	0	0	194890	0	0	269950	789550	0	0
Abcc3	0	763510	0	721010	851570	0	766240	0	743180	0	827100	663550
Acot2;Acot1	1759700	2246700	2632200	2603100	4824700	2587900	2557900	872110	1629700	1415500	1562800	965920
Ddx6	6751700	6918000	6008400	7547500	7156400	7942200	5348200	7304500	6711400	5385400	6061100	5270000
Fam63a	1815200	1046800	0	1740700	1595100	1739500	0	0	1402900	1546700	1851500	0
Ap2a2	1458700	1881800	1541200	1425900	1563800	1540200	1474500	1011400	1474600	1447200	0	1450300
Uso1	2699300	3657800	2643000	3043000	3054400	4261100	2512400	2541000	2764200	2731300	3348000	2055600
Mrpl46	967250	2014100	1283600	1838500	934250	1445600	0	0	935860	1503100	0	1503600
Naga	843430	841900	415380	1101300	0	928520	455080	0	344500	161240	491300	0
Pcbp2	51451000	50173000	51549000	51584000	46532000	78718000	44233000	25168000	44159000	54471000	50971000	55543000
Psmd12	16747000	15948000	13483000	14539000	14239000	14651000	10879000	5165900	11214000	10688000	14966000	10576000
Psmd13	12441000	16053000	10558000	15658000	11172000	13294000	4508400	4853100	8875500	6129800	14125000	4190600
Psmd6	10064000	8609300	6605900	8489800	9265900	9953200	5151000	5104400	7969500	10188000	9183400	6685100
Qars	7522000	8090200	5793100	5284900	7804100	7471600	4749600	2109400	6076200	6297000	7887300	3855500
Samhd1	3696800	3764800	1804900	3115900	5200700	3745400	1632600	2515500	2216600	2596300	3038000	1723300
Senp3	0	0	0	0	1633100	1691400	0	0	0	0	0	0
Slc7a5	2418900	3252200	1912500	2676500	0	2417500	0	0	2579000	0	2737700	0
Sphk2	839930	929660	938280	1085300	737910	1211200	0	0	0	901060	894970	0
Cad	13678000	8391800	11924000	3994100	10430000	7718600	10450000	13049000	9684900	11833000	10437000	11911000
Cpsf3	0	0	0	0	599200	0	620270	526560	450970	523330	577850	0
Huwe1	1909700	782860	0	0	1166800	644440	1041000	1172400	998410	0	1211500	1033400
Iap	9417100	8746000	7443300	9465500	12581000	7804700	12650000	12952000	8298700	9259700	7042300	8790400
Mgea5	514670	644150	0	0	489790	0	482400	593720	563280	0	0	0
Capn2	3198400	2876800	2154600	597100	2570600	2991100	3699300	5641900	2392800	3470500	2267400	3377900
Rab21	3704900	2463500	0	3743600	4309800	0	4068700	4255400	4751700	4109100	3675500	2479700
Parl	1350300	1082900	0	0	1539300	1126200	1674900	0	1312100	971120	1519600	1177700
Plaa	5213800	3154000	2576900	3002000	4910700	2774000	3686100	6973000	5314600	5882700	2909500	5069000
Pnkp	0	4364800	0	0	4506500	5349600	3092000	3517000	4249000	3886600	0	3512500
Dnm2	15240000	13038000	13722000	9640400	13257000	11601000	12975000	15030000	12653000	15867000	10076000	16128000
Rnf130	1802500	0	0	0	2105400	1343200	2162200	2570900	1397800	1778600	2578500	2346000
Spp1	2814300	2055400	1960500	1966500	1941100	1482800	2695200	3599000	5467100	5013800	1886300	4353800
Arpc2	20725000	27073000	12418000	32640000	18564000	24287000	6168600	3460400	23909000	18599000	11910000	4210400
Cops4	5845500	7523100	5537200	6346000	5229700	6486100	3062600	3139800	5762800	8094900	3899400	3927900
Pafah1b2	2631800	3004700	1926400	3674200	2425100	2703100	1330600	0	3110000	2488900	1833000	1286600
Pla2g4a	8180600	9922200	8105600	6736200	5426100	8558900	7045600	3558600	6810700	8816300	6282600	6224900
Pls3	5565100	10815000	5560400	10753000	6315500	9324000	4935900	3881000	9436300	8738500	4716200	4487100
Ppp2ca;Ppp2cb	9901200	8467300	0	8814000	7178700	8661500	0	3891300	12211000	5596600	7067200	4354500
Ppp2r1a	34798000	52639000	30089000	49535000	32443000	41951000	21332000	24402000	40481000	29359000	33472000	23651000
Vps29	2507800	2655600	2812100	2999400	3455900	0	0	0	0	2846000	0	0
Rnpep	16107000	21601000	16763000	16652000	15934000	17570000	12662000	10037000	17684000	22740000	12629000	14421000
Psmb3	5985700	8780300	3347700	9557900	6653400	7414700	2970000	2781600	5394600	5917200	5058800	2838000
Ddx39a	6708200	4729600	4356500	8146100	3921600	2656600	6279500	1267700	3549700	9940800	6475600	3889600
Dpp3	0	5554800	3963700	3963200	5157200	3822000	4219900	3261700	5517700	5644700	3440000	2898200
Tpp2	10541000	8812900	5479000	6551900	5887300	6440900	3835800	3739800	4775800	6524600	6009500	4438500
Pafah1b1	8410400	6443500	2811100	0	7498700	3869400	3930900	4978000	5297600	5052800	3572100	3376900
Sqstm1	21638000	24540000	8232800	17240000	13096000	11874000	14420000	15039000	21147000	21866000	8531000	11225000
Hist1h2bc;Hist2h2bb;Hist1h2bh	37235000	40582000	171590000	53794000	44231000	44603000	180660000	118910000	150680000	125930000	140000000	99347000
Ube2d3;Ube2d2b;Ube2d2	0	0	0	0	0	0	0	0	4335100	4125800	0	3131900
Wwp2	598930	537850	0	0	766330	600000	880810	672700	762720	845750	952870	840850
Impa1	3222400	7175200	2208100	7077500	4320800	5680500	2284300	2889400	3902500	3541600	0	2127100
Upf1	2737400	3699600	2542900	2223100	3924300	3431500	2342000	2762000	3278400	3430000	2811000	1984200
Usp5	18983000	22139000	18689000	22362000	18697000	20171000	14846000	12733000	19516000	25853000	16750000	17139000
Xpnpep1	6796100	6837200	4596300	5639000	5027500	6597500	2914300	3557500	8187200	7576600	4764300	4271000
Drg2	612800	541100	0	471480	520590	478270	411160	542640	517820	527580	605250	478460
Eif4a1	237460000	219130000	186730000	221400000	213640000	193120000	178570000	206080000	213140000	210470000	193790000	187120000
Ola1	5874600	8789300	3957700	5690200	4082600	5292000	4024500	5454200	6222300	4771900	3306300	4953300
Ubr4	837380	759420	760070	770840	619440	617980	0	512240	0	919890	620570	935650
Abce1	10195000	9539800	9938200	11188000	9816400	8768400	6181400	10665000	13012000	12700000	9864700	7760100
Acy1	1043700	0	1085800	1325600	979590	1021800	987900	528940	1047600	1911200	811960	978740
Eno1	235710000	306940000	162830000	288900000	212580000	235450000	179990000	209020000	284940000	278150000	158940000	209610000
Atg7	3909800	2524700	2580800	2847300	1974600	2639100	2267000	1746800	3826400	3719700	2471100	2482200
Bax	4377300	6368600	5084600	7825000	4830700	6301500	3560300	4515600	6763300	6322100	3501500	3986300
Cast	207320	221260	0	132540	0	93246	0	0	288050	630880	0	0
Anpep	2399500	1970700	3111000	4411300	3077600	2457000	5704500	3764000	3175100	2019800	2715200	1812700
Derl1	2186100	2649900	2749100	2174600	2927900	2324700	3488500	2704800	2095800	2777900	3464000	2957600
Irgm1	3365500	1928800	4294600	1823700	3417500	3023500	4021900	3502000	2824500	0	3858100	3337800
Spcs2	2475800	2128800	3256100	5132300	4246200	2426100	8185300	3811800	2190400	5525200	3687300	4531900
Mbd3	442320	412310	0	0	0	0	0	0	0	0	0	0

ER

Genenames	LFQintensityA1	LFQintensityA2	LFQintensityB1	LFQintensityB2	LFQintensityC1	LFQintensityC2	LFQintensityE1	LFQintensityD2	LFQintensityD1	LFQintensityE2	LFQintensityF1	LFQintensityF2
Ecm29;AI314180	2879500	2017900	3739900	1854400	2424600	2030000	2874500	1338100	2320500	2651200	3059700	2595300
Tor3a	4003100	3043900	2642600	3733700	2639100	2001200	3415400	1769600	2500200	3359600	0	3229200
Tnpo3	2817900	2958800	2263900	1858600	1516600	1811000	1045900	0	1220900	1915400	3022000	2129700
Sigmar1	4578700	2354100	4425400	2746800	3208200	2224200	3023700	1683300	2819800	2589100	3399100	2781100
Emc1	6986500	6053200	6733600	5946900	6794300	6310700	5925300	1622200	4487100	4486000	7797600	4517200
Surf4	4374700	3105600	4922700	2154600	3656200	2090200	2898200	2024300	1638900	3427100	5011600	2469100
Hadhb	5396800	9649900	7012200	11733000	8834400	10776000	5868300	3454900	4985300	5487100	6837300	10781000
Sec61a1	5453900	8019000	7606300	4551300	6821900	6069100	3808200	1761600	2645900	3510500	8078700	2579100
Bak1	4812000	4963200	8871900	7047500	6354600	6067400	4891200	0	2749600	4661800	5419300	5455300
Zmpste24	905150	1716200	0	1613600	981940	2326400	1237600	0	777180	1494500	1263900	1849800
Stt3a	12692000	9950400	16557000	7674200	16273000	9451000	11378000	7128300	10473000	6387800	16719000	10731000
Npc1	1806200	4094400	5476600	4275500	3054600	4902100	4583600	1801100	1502000	2399000	2288800	4163100
Ncln	1844100	4404800	3749100	3141900	3971700	3364200	1586100	0	942260	1051200	4724300	2944300
Ccdc47	9422800	7036800	9772100	9224400	7638500	8047300	8221800	5192800	2316700	2569500	9519000	7235600
Cept1	644470	389080	2837400	0	732650	564790	2640800	1722800	963520	1585800	696050	745090
Lrrc40	743130	991900	829330	650090	754550	675100	0	677110	643280	818870	0	904920
Tecr	5672200	6139300	4717100	3182500	8488400	5873000	7515600	5287600	4982300	4321300	10489000	6963100
Scfd1	3766300	3455600	4940900	2958700	5264600	4644800	5211000	4057800	3538800	3344300	3447300	3411400
Dpm1	1050600	948720	892610	1105600	1035100	895940	838260	0	791010	512060	3212100	792500
H2-K1	33131000	31238000	29815000	32153000	24174000	29010000	35687000	25721000	26458000	26083000	35321000	34761000
Cdipt	3253100	3182400	3402600	3815000	3589100	3703700	3864900	2657100	3272200	2013100	5665800	3672700
Lmf2	9153300	3951700	4482900	2992400	7056600	4951400	4199800	6857000	3534700	2862300	5845500	4237500
Pgrmc1	0	2554400	2104800	3043100	2042600	3232500	3119200	0	0	2042800	1746100	2966300
H2-D1	247470000	171050000	183720000	139460000	176280000	133870000	178310000	192490000	132440000	93152000	223630000	133140000
Wdfy4	754250	619620	707670	2540400	596870	560890	640560	522690	0	1136600	924500	672470
Arf5	3451400	3100700	0	3237200	0	0	3387000	2845700	2883500	0	0	0
Dnajc3	2840800	2961200	2379500	5224600	2987100	2405100	2079200	1909300	2851100	2225800	2748200	0
Ubxn1	1241400	2968000	1344900	4243100	1357100	1395900	1256800	0	1400500	1287600	1075400	0
Mgst3	2329200	2785500	4510000	4024600	2868400	3716300	1424500	570030	2250700	1828500	3370100	1678400
Xdh	4282300	5149500	4640800	7009800	3578100	3871900	1813200	1349900	3102600	1757800	3509400	1074600
Arl6ip5	2272500	4903700	2502800	6491700	3929400	4035300	1953700	2069800	2283300	2948600	3311300	2247800
Stx16	205120	0	0	840320	0	512250	0	0	87848	86104	182680	0
Tspo	5207100	4103000	3660500	7521000	2952600	4005900	1633100	1107400	1524100	1227200	2988900	577020
Pigt	1036900	711470	991790	759440	973240	620350	794160	0	713790	0	0	711760
Gba	3174100	4659500	3984600	7737000	2884600	5693000	1985600	0	1177000	1708300	3875300	1113400
Srpr	4377500	2565800	3208800	4512200	2921600	2305200	1417500	3169300	1695200	0	2199300	2529200
Osbp	931830	1104600	960400	2152400	1076700	1113200	0	0	1159700	0	0	0
Srp54;Srp54c	7492600	12873000	0	18138000	9937900	7427000	0	0	8959300	0	7205300	6981000
Tsn	1675600	3344400	0	4985800	0	3139400	0	0	1274700	3347500	0	1514600
Rhoa	1780900	5543800	1586100	5352600	2441000	4192200	1642700	2524500	2757600	3097600	2922500	2148900
Fam129a	16291000	16317000	16104000	18681000	16822000	15433000	13013000	12947000	13834000	15888000	13149000	14168000
Rab3gap1	1288900	523700	1377300	1283500	1163800	1375100	0	0	1281000	1211800	1077400	0
Lman2	0	0	0	2716000	2111100	0	0	0	1863500	0	1985900	2086700
Asl	3901000	2062100	2435000	3172800	2506300	2273300	1231000	1384800	2032400	2793000	2018900	3030500
Oas1a;Oas1g	5275600	4257500	3468900	5837200	4523500	6500800	0	0	3303400	3435000	6743600	4119600
Hsd17b11	2333000	3203900	2323300	3339200	1807500	2422500	0	0	1769100	1669300	4022600	2247400
Dnajb11	3948800	5144200	2816400	6045900	3544600	4139200	2662900	2295900	3939800	3267000	5169700	4073100
Uso1	2699300	3657800	2643000	3043000	3054400	4261100	2512400	2541000	2764200	2731300	3348000	2055600
Xpo1	13418000	12151000	14561000	8316200	14259000	10657000	9312000	9141700	10853000	11057000	14463000	11358000
Ccdc115	0	2432000	1650100	2575700	1751000	2014000	1598000	1369600	1629600	1798600	1745400	1756800
Unc93b1	4873200	6184800	5563200	4755400	6240100	5183700	4112400	4786800	5642400	3460500	5303300	4078900
Rab21	3704900	2463500	0	3743600	4309800	0	4068700	4255400	4751700	4109100	3675500	2479700
Vim	130210000	50727000	149310000	152270000	263010000	107490000	279000000	352000000	287750000	279440000	103000000	111410000
Rpl5	91495000	112350000	87982000	92036000	108650000	91770000	127560000	108630000	105670000	122560000	79852000	112160000
Rcn2	13215000	5429500	6730400	10006000	8299900	7428300	9584400	7223400	10370000	10318000	10813000	11144000
Tmed9	15552000	12272000	13161000	21451000	11742000	13404000	17742000	19205000	17325000	16660000	11618000	17575000
Fads1	0	2559700	0	1954500	0	2613100	3415600	3216200	2881400	0	2596300	3181900
Rps29;Gm10126	6374900	13597000	10062000	16144000	13864000	14438000	14993000	20915000	14712000	13598000	7740700	12006000
Tmx3	4267900	3510400	3883400	3803100	5425700	4162600	4745500	3600900	4718700	4064800	4057700	5097000
Sec23b	8534800	12470000	9876000	9662200	12957000	12988000	7260700	10438000	13135000	14211000	8276300	9620900
G6pdx	43768000	57243000	34901000	47844000	41488000	44355000	29499000	35779000	51626000	48624000	32960000	38492000
Kiaa1598	0	2019000	0	1877200	0	2636500	0	0	2653500	2633700	0	0
Calm1	23074000	39540000	18031000	25052000	30876000	38004000	21799000	9388200	35692000	26647000	22554000	10985000
Pafah1b2	2631800	3004700	1926400	3674200	2425100	2703100	1330600	0	3110000	2488900	1833000	1286600
Pla2g4a	8180600	9922200	8105600	6736200	5426100	8558900	7045600	3558600	6810700	8816300	6282600	6224900
App	0	2162500	0	1408100	0	1169700	0	0	1452500	1511500	0	0
Aimp1	16983000	16646000	11536000	16983000	16388000	15128000	11182000	11295000	15466000	14447000	14707000	12417000
Tap2	2668200	3600600	2531000	0	2001400	2835400	0	0	0	1532600	0	0
Sqstm1	21638000	24540000	8232800	17240000	13096000	11874000	14420000	15039000	21147000	21866000	8531000	11225000
Pkm	1232500000	1183300000	907320000	823130000	902760000	811460000	871800000	983280000	1055000000	1234700000	820840000	957730000
Lars	38713000	35252000	33064000	22375000	37510000	31094000	27751000	27128000	29237000	37812000	26920000	35872000
Pgd	92796000	62117000	60395000	48824000	61742000	50412000	47773000	60190000	61557000	66180000	60190000	58952000
Pafah1b1	8410400	6443500	2811100	0	7498700	3869400	3930900	4978000	5297600	5052800	3572100	3376900
Gnai3	3856000	3015100	2116600	2626500	3783400	2161000	3105100	1821200	3725200	1947200	4469200	2339300
Ero1l	29556000	27192000	16826000	20267000	16562000	19273000	15024000	14928000	19989000	14480000	20797000	14743000
Safb2	440510	387250	0	142590	0	0	0	0	0	0	0	0
Rps12	21578000	31401000	24758000	27022000	21965000	28567000	27732000	21004000	34115000	34279000	24981000	24066000
Sgta	0	0	0	0	0	194890	0	0	269950	789550	0	0
Atp5d	5730600	1997900	2101900	7080500	8848800	2024700	3284000	3492900	10786000	5026400	6482100	2769100
Cast	207320	221260	0	132540	0	93246	0	0	288050	630880	0	0
Nploc4	0	1699100	0	2283600	1799500	1373900	966740	0	0	3176400	1317300	0
Tpd52	17806000	31322000	21287000	32719000	18230000	21517000	19936000	25886000	29413000	36597000	14478000	21482000
Pebp1	7808900	10868000	5982900	13554000	9442800	11430000	4993400	8819100	14057000	12616000	4830400	5964700
Aco1	390060	416900	361910	413190	302050	323880	337550	362020	349590	375650	0	0
Rac1;Rac3	4234300	9043800	3222900	7919800	4491900	7810600	4476600	3444900	8173400	6819000	2633000	3594100
Ncf1	3370600	1688800	3479300	3177200	3223600	2249600	3379300	4482700	3101300	3187000	2877600	3256200
Rpl18	306340000	250080000	409690000	186370000	360370000	222600000	602580000	1068000000	372430000	433680000	324640000	517760000
Spcs2	2475800	2128800	3256100	5132300	4246200	2426100	8185300	3811800	2190400	5525200	3687300	4531900
Vti1b	0	2016700	1904800	2393800	1600600	2633400	2184200	2410300	2188000	2077000	0	2138700
Mlec	8393700	4592600	5981600	4170200	6207100	5326300	7291500	8148900	7072600	5134900	5981200	8641100
Rpl34	31803000	24141000	27872000	26610000	45455000	24865000	52622000	65685000	33280000	33609000	31615000	51706000
Anpep	2399500	1970700	3111000	4411300	3077600	2457000	5704500	3764000	3175100	2019800	2715200	1812700
Ccdc88b	24637000	12071000	13349000	3772300	14845000	11161000	9070100	38939000	13203000	13273000	8454300	26183000
Retsat	541910	0	916670	0	0	780660	1167200	1161200	858520	644350	1027400	0
Elovl1	0	0	0	0	2892000	0	11515000	11802000	0	0	0	0
Derl1	2186100	2649900	2749100	2174600	2927900	2324700	3488500	2704800	2095800	2777900	3464000	2957600
Prdx4	121790000	87744000	70728000	91894000	79537000	109660000	101340000	150070000	82648000	94620000	111820000	157150000
Itga5	352110000	158170000	315490000	111490000	192730000	146390000	383550000	400070000	226380000	151590000	376720000	414930000
Asph	48518000	35071000	41680000	34585000	42937000	34639000	46308000	53805000	42220000	33422000	58885000	51179000
Alg5	0	1789200	1808900	1374500	2975300	1795400	4008500	2853400	1505700	0	1754300	2176300
Abcg1	0	0	0	0	2642100	1460300	1898800	0	0	0	0	1883200
Flrt2	4348400	1750900	6333200	1737500	2077300	1751100	4621500	6870100	1646300	2823900	5271500	3226200
Nrp1	154960000	74785000	205610000	71719000	125470000	85004000	213050000	215540000	94196000	66175000	255900000	249340000
Ptgs2	12700000	3647500	6086200	1876300	11061000	2862500	13319000	14156000	3977500	2081100	18291000	9955600
Atp1a1	205840000	170920000	230180000	174350000	195680000	164580000	248970000	264830000	176980000	153670000	226190000	229270000
Dhrs9	1346400	2460000	0	1824500	1702400	2093900	0	2502300	2040400	1604800	0	2162100
	3610025350	3023008100	3201677780	2570890170	3136427400	2554368796	3665623870	4399266350	3243754398	3312613164	3209833830	3561732760

Golgi apparatus

Genenames	LFQintensityA1	LFQintensityA2	LFQintensityB1	LFQintensityB2	LFQintensityC1	LFQintensityC2	LFQintensityE1	LFQintensityD2	LFQintensityD1	LFQintensityE2	LFQintensityF1	LFQintensityF2
Surf4	4374700	3105600	4922700	2154600	3656200	2090200	2898200	2024300	1638900	3427100	5011600	2469100
Cand1	4968700	4763800	5376200	4669200	4501600	4943100	4205600	1891500	3044500	4033300	7087900	4611900
Dynlt1	1679300	1621200	1613200	2469400	1309300	1575000	1156000	1689900	1902700	2033300	1919700	2324700
Gnaq	5200900	5167900	7423300	5825300	6967900	6158000	6269700	3578800	4773700	4234400	5515300	6977500
Cltc	337320000	263460000	307880000	142410000	350650000	250570000	252060000	225510000	219680000	223900000	344470000	268440000
Fcgr3	2691400	0	13818000	0	0	0	0	0	0	0	8202200	10093000
H2-D1	247470000	171050000	183720000	139460000	176280000	133870000	178310000	192490000	132440000	93152000	223630000	133140000
Cdipt	3253100	3182400	3402600	3815000	3589100	3703700	3864900	2657100	3272200	2013100	5665800	3672700
Cept1	644470	389080	2837400	0	732650	564790	2640800	1722800	963520	1585800	696050	745090
H2-K1	33131000	31238000	29815000	32153000	24174000	29010000	35687000	25721000	26458000	26083000	35321000	34761000
Gak	0	106360	0	0	96880	135260	106800	0	56836	0	219040	0
Cd14	40157000	34925000	57529000	41813000	44755000	35429000	56297000	38064000	31209000	20321000	63056000	42979000
Scfd1	3766300	3455600	4940900	2958700	5264600	4644800	5211000	4057800	3538800	3344300	3447300	3411400
Lrp1	178770000	119050000	219640000	117210000	168880000	108190000	193600000	149940000	90154000	80233000	214450000	140560000
Rab31	1276500	2070900	0	2305300	0	1672700	2265300	0	1399100	1677200	0	1723500
Neo1	7006700	2820800	17118000	5285000	7624900	3792900	12459000	9662900	1801800	2329400	13791000	11717000
Arl1	1376900	3433800	1603700	2757800	1405100	0	1365700	0	1028300	0	0	1089800
Atp6v0c	5003300	7807700	6948700	11104000	6832100	10306000	5136400	6289000	6100000	6907600	6888700	5157900
Acbd3	1541400	1868500	0	3720200	1691600	3027900	0	0	1732900	0	1595000	0
Arf5	3451400	3100700	0	3237200	0	0	3387000	2845700	2883500	0	0	0
Snap23	0	0	0	1619100	1519500	1550500	0	0	0	0	0	0
Stx16	205120	0	0	840320	0	512250	0	0	87848	86104	182680	0
Tmed5	0	1563200	0	5279600	803560	1292500	0	0	0	0	0	0
Dpy30	1096700	3325000	1806300	5585100	2076500	5014700	2279800	1916700	1524800	3426000	2321300	2345100
Glb1	1213400	2198700	1020000	3627700	1505100	1778200	604110	0	0	570420	1705500	640000
Sart1	932520	1475300	0	2204100	0	1551700	0	0	0	0	0	0
Scly	1761600	2241200	1537000	3773900	1649300	4066600	0	1046600	1700800	1908700	1652600	1276300
Snx12	869700	1980100	0	2239900	835630	1122400	751440	601940	1069000	1074400	0	834970
Snx9	2653500	1686500	2292300	0	1986300	1597800	0	2142400	1857800	2064000	1723400	2090900
Tsnax	441620	975270	387210	1696500	557790	1216700	455700	0	264540	355570	0	0
Ap1b1	8705000	9064100	10646000	11174000	8279400	9118600	6497800	3233300	7441200	9881900	7333900	9535900
Osbp	931830	1104600	960400	2152400	1076700	1113200	0	0	1159700	0	0	0
Macf1	5976200	7346900	0	10597000	0	8671800	0	0	0	7931200	0	0
Clint1	7429900	10135000	6341000	10858000	9370000	11464000	5930200	6944500	7558700	6088700	5774400	4900600
Ccdc115	0	2432000	1650100	2575700	1751000	2014000	1598000	1369600	1629600	1798600	1745400	1756800
Copg2	64269000	64452000	65491000	53015000	50822000	61088000	31171000	45801000	44083000	47688000	56491000	40133000
Lman2	0	0	0	2716000	2111100	0	0	0	1863500	0	1985900	2086700
Rab3gap1	1288900	523700	1377300	1283500	1163800	1375100	0	0	1281000	1211800	1077400	0
Uso1	2699300	3657800	2643000	3043000	3054400	4261100	2512400	2541000	2764200	2731300	3348000	2055600
Coro7	1443500	2192800	0	1505000	1490900	961940	1843600	2221500	2469800	5781500	1226100	1787100
Exoc4	1181200	1162800	0	1047800	1548100	1309500	1288700	0	1763600	0	1338500	1244200
Golim4	301970	0	284470	58457	485630	80120	501430	593420	655700	539680	604240	0
H1f0	1878400	2732500	3379200	5658500	0	1776900	3755700	0	5234500	4563700	3378700	2904200
Huwe1	1909700	782860	0	0	1166800	644440	1041000	1172400	998410	0	1211500	1033400
Myo18a	426930	418800	0	0	644050	426190	706760	876230	466500	835030	0	0
Rab21	3704900	2463500	0	3743600	4309800	0	4068700	4255400	4751700	4109100	3675500	2479700
Tmed9	15552000	12272000	13161000	21451000	11742000	13404000	17742000	19205000	17325000	16660000	11618000	17575000
Dnm2	15240000	13038000	13722000	9640400	13257000	11601000	12975000	15030000	12653000	15867000	10076000	16128000
Ap3d1	1162500	1164200	688980	0	1318400	1177700	1045700	1119600	1028500	1262000	1433200	1093500
Spp1	2814300	2055400	1960500	1966500	1941100	1482800	2695200	3599000	5467100	5013800	1886300	4353800
Actr1a	11491000	12095000	7773400	12630000	9827500	9428200	10069000	5303200	10473000	9645500	8554200	9517500
Lamp1	11313000	13663000	9823600	12806000	12995000	13989000	6185000	6477100	11962000	7943900	0	5952500
Aimp1	16983000	16646000	11536000	16983000	16388000	15128000	11182000	11295000	15466000	14447000	14707000	12417000
App	0	2162500	0	1408100	0	1169700	0	0	1452500	1511500	0	0
Golph3	0	0	0	0	865640	389970	0	0	538010	462960	0	0
Pafah1b2	2631800	3004700	1926400	3674200	2425100	2703100	1330600	0	3110000	2488900	1833000	1286600
Pla2g4a	8180600	9922200	8105600	6736200	5426100	8558900	7045600	3558600	6810700	8816300	6282600	6224900
Sec23b	8534800	12470000	9876000	9662200	12957000	12988000	7260700	10438000	13135000	14211000	8276300	9620900
Dbnl	3999000	11788000	5941600	14220000	13581000	10110000	4227100	8359400	7397700	10258000	5413100	3245000
Vps29	2507800	2655600	2812100	2999400	3455900	0	0	0	0	2846000	0	0
Ankfy1	6713500	4602700	4806900	4932400	6622600	4131700	4575100	5087100	5461700	5325900	5436500	4230300
Tubb4b	79594000	98071000	64450000	71951000	76481000	73885000	71543000	69370000	70488000	85278000	63930000	72225000
Gnai3	3856000	3015100	2116600	2626500	3783400	2161000	3105100	1821200	3725200	1947200	4469200	2339300
Pafah1b1	8410400	6443500	2811100	0	7498700	3869400	3930900	4978000	5297600	5052800	3572100	3376900
Mapk3	2465500	4422500	2267600	4330700	2869700	4109000	1797400	3297200	4607600	4664200	2111200	0
Nudc	11348000	14751000	5608900	21004000	10510000	17075000	5009700	7905600	18741000	19162000	8010400	8389200
Pebp1	7808900	10868000	5982900	13554000	9442800	11430000	4993400	8819100	14057000	12616000	4830400	5964700
Rac1;Rac3	4234300	9043800	3222900	7919800	4491900	7810600	4476600	3444900	8173400	6819000	2633000	3594100
Rps12	21578000	31401000	24758000	27022000	21965000	28567000	27732000	21004000	34115000	34279000	24981000	24066000
Tpd52	17806000	31322000	21287000	32719000	18230000	21517000	19936000	25886000	29413000	36597000	14478000	21482000
Anpep	2399500	1970700	3111000	4411300	3077600	2457000	5704500	3764000	3175100	2019800	2715200	1812700
Cd44	45784000	36075000	70653000	25622000	50693000	30436000	89153000	79257000	29681000	20419000	77862000	39848000
Irgm1	3365500	1928800	4294600	1823700	3417500	3023500	4021900	3502000	2824500	0	3858100	3337800
Pi4k2a	2030000	1606500	2474100	2307500	2142900	1857600	2831800	3528400	1490700	1657700	1837000	2762600
Tlr2	9307400	3468300	12720000	4059300	7334500	4573100	11248000	14021000	6459400	4734900	12738000	12106000
Atp1a1	205840000	170920000	230180000	174350000	195680000	164580000	248970000	264830000	176980000	153670000	226190000	229270000
Ccdc88b	24637000	12071000	13349000	3772300	14845000	11161000	9070100	38939000	13203000	13273000	8454300	26183000
Itga5	352110000	158170000	315490000	111490000	192730000	146390000	383550000	400070000	226380000	151590000	376720000	414930000
Vps26a	3278800	4662200	3050400	5536100	3431300	4091300	5402400	0	3119900	3121300	2600700	5320400
Dctn1	3545900	3467600	0	2444700	3136900	2573500	0	3938400	3385400	2522200	2745200	3893200
Vti1b	0	2016700	1904800	2393800	1600600	2633400	2184200	2410300	2188000	2077000	0	2138700

Extracellular

Genenames	LFQintensityA1	LFQintensityA2	LFQintensityB1	LFQintensityB2	LFQintensityC1	LFQintensityC2	LFQintensityE1	LFQintensityD2	LFQintensityD1	LFQintensityE2	LFQintensityF1	LFQintensityF2
Apoa1bp	2752400	2493100	0	2655000	0	2388100	2435200	3019700	0	0	0	2070800
Icoslg	976700	525210	2025200	534870	643000	0	1325600	0	0	0	1035100	700450
Il6st	1783600	2060600	0	3150000	1264000	1675700	2626100	1707000	1207100	1931400	3399800	2961300
Krt10	4282400	4028200	6502500	0	4028300	4790400	8436900	2087900	0	2508700	6125000	3627700
Neo1	7006700	2820800	17118000	5285000	7624900	3792900	12459000	9662900	1801800	2329400	13791000	11717000
Nrcam	6575500	3840400	8845900	4261600	5464600	4166900	7145200	5318800	2743800	2222500	10252000	4468800
Apobr	1406300	3740900	1890500	6167200	0	3285500	2311600	2201900	1769100	3196900	1733500	3070600
Cd180	8861400	7988800	6456700	14707000	6823500	9161000	4043500	4314800	5048500	2331200	9307500	2181000
Dnajc8	0	2177700	0	4406100	0	1915600	0	1045900	1501900	1603000	1119900	1163600
Sfn	20207000	0	77773000	3794200	0	0	8360400	0	8844700	9068000	3137900	8312400
Snx9	2653500	1686500	2292300	0	1986300	1597800	0	2142400	1857800	2064000	1723400	2090900
Lgals3	150570000	262720000	194200000	345390000	213440000	250610000	157260000	175870000	215020000	250590000	138830000	190070000
Anxa5	170480000	308090000	187920000	331330000	174600000	228950000	154800000	126090000	205750000	256070000	126400000	188400000
Fabp5	33345000	77611000	38089000	99425000	52610000	70194000	43633000	32121000	50308000	61933000	38788000	42795000
Rsu1	4610300	7091600	4101400	7297900	2719100	5767600	2523900	2947300	5065600	3663600	3860500	2464300
Rab35	0	1443600	1922300	1789300	0	1328000	0	0	1489800	1430800	0	0
Fn1	1280300	905100	0	725350	2244700	0	0	0	0	0	0	0
Exoc4	1181200	1162800	0	1047800	1548100	1309500	1288700	0	1763600	0	1338500	1244200
Plaa	5213800	3154000	2576900	3002000	4910700	2774000	3686100	6973000	5314600	5882700	2909500	5069000
Spp1	2814300	2055400	1960500	1966500	1941100	1482800	2695200	3599000	5467100	5013800	1886300	4353800
Wwp2	598930	537850	0	0	766330	600000	880810	672700	762720	845750	952870	840850
Cap1	122280000	189350000	108550000	155390000	111030000	136570000	90481000	71768000	154490000	162470000	78989000	92239000
Ckb	65898000	104210000	57986000	113000000	62261000	84798000	46840000	35939000	82531000	96744000	47489000	63618000
Aimp1	16983000	16646000	11536000	16983000	16388000	15128000	11182000	11295000	15466000	14447000	14707000	12417000
Pfn1	76068000	113500000	55841000	109520000	72629000	85127000	65491000	66664000	93370000	96510000	53163000	67804000
Pafah1b2	2631800	3004700	1926400	3674200	2425100	2703100	1330600	0	3110000	2488900	1833000	1286600
Pla2g4a	8180600	9922200	8105600	6736200	5426100	8558900	7045600	3558600	6810700	8816300	6282600	6224900
Pycard	2627700	5122000	2184700	4833600	2512100	3283300	2083800	1405900	3596000	4966800	2277100	1951400
Pnp	2624200	3087600	2075700	3048500	2792400	2765000	0	2195000	2954500	2488600	1927700	2757000
Rnpep	16107000	21601000	16763000	16652000	15934000	17570000	12662000	10037000	17684000	22740000	12629000	14421000
Gars	63891000	63364000	41230000	48125000	51167000	46137000	41514000	50847000	56004000	53387000	48075000	40282000
Lgals1	23970000	41147000	10602000	31519000	26696000	37632000	19755000	26456000	37023000	35248000	32476000	14860000
Nampt	16485000	18286000	11876000	13306000	11351000	10706000	10999000	13546000	12423000	12782000	12548000	11156000
Gpi	103800000	144600000	93838000	136070000	88987000	100970000	84604000	69328000	124850000	151630000	62578000	100600000
S100a11	1075500	1150700	0	942070	0	0	0	0	1427800	4086100	0	0
Cast	207320	221260	0	132540	0	93246	0	0	288050	630880	0	0
Grn	0	2400500	2286600	2428200	0	2198400	3097400	2413800	0	2283900	0	0
Pebp1	7808900	10868000	5982900	13554000	9442800	11430000	4993400	8819100	14057000	12616000	4830400	5964700
Tln1	172670000	227750000	149560000	200520000	153810000	191460000	138610000	169980000	207900000	249680000	119310000	144050000
Ide	1021100	1175900	805720	1023200	963530	1101500	1012000	0	1089500	1333600	0	0
Alcam	58710000	38305000	75313000	31607000	48484000	41980000	77539000	74955000	41911000	32820000	95880000	67948000
Anpep	2399500	1970700	3111000	4411300	3077600	2457000	5704500	3764000	3175100	2019800	2715200	1812700
Cd44	45784000	36075000	70653000	25622000	50693000	30436000	89153000	79257000	29681000	20419000	77862000	39848000
Cd72	31111000	18321000	49949000	9747400	25692000	15769000	60925000	95367000	34350000	28215000	43115000	72792000
Cr1l	3522300	1934700	7351700	1377100	2853200	1633600	8867000	5916600	1857000	1098300	6490300	4611100
Emilin2	9087900	3592400	8014900	3443500	5389600	2806600	5607100	9561300	5392000	3149200	4074000	4896200
Flrt2	4348400	1750900	6333200	1737500	2077300	1751100	4621500	6870100	1646300	2823900	5271500	3226200
Itgal	3228500	1362500	1396000	1123800	2052900	1007300	1862000	3322000	1457100	1072300	2113000	1595400
Itgam	43038000	56476000	52780000	61296000	41790000	46100000	55507000	69961000	39885000	44149000	59520000	47062000
Rpl39	12598000	10588000	23521000	12037000	0	15708000	22855000	19360000	15419000	19859000	23187000	0
Serpinc1	2394000	2354300	2445500	1929000	3194400	3683600	3181500	2705500	3140200	1232800	3373300	3377700
St14	2865800	2171300	4895800	2052200	2043700	1775000	5986100	3711900	1958100	1664900	3358400	2519300
Itga5	352110000	158170000	315490000	111490000	192730000	146390000	383550000	400070000	226380000	151590000	376720000	414930000
Prdx4	121790000	87744000	70728000	91894000	79537000	109660000	101340000	150070000	82648000	94620000	111820000	157150000
Dctn1	3545900	3467600	0	2444700	3136900	2573500	0	3938400	3385400	2522200	2745200	3893200
	1827461750	2097823820	1826805920	2076604830	1579181260	1777751946	1784310710	1852856500	1843075870	1955289230	1683950470	1882895100

Recptor

Genenames	LFQintensityA1	LFQintensityA2	LFQintensityB1	LFQintensityB2	LFQintensityC1	LFQintensityC2	LFQintensityE1	LFQintensityD2	LFQintensityD1	LFQintensityE2	LFQintensityF1	LFQintensityF2
Cyp20a1	1451000	1465000	1890500	2064300	1763200	2530200	1001400	0	726160	934110	1579600	0
Fcgr3	2691400	0	13818000	0	0	0	0	0	0	0	8202200	10093000
Tlr13	3257900	1883800	2435800	3111900	2695300	2096500	1847400	0	1894000	1199600	3038000	1762500
H2-D1	247470000	171050000	183720000	139460000	176280000	133870000	178310000	192490000	132440000	93152000	223630000	133140000
H2-L	217200000	106510000	143450000	64349000	126340000	64055000	129410000	146350000	87362000	67044000	140870000	113350000
Cd14	40157000	34925000	57529000	41813000	44755000	35429000	56297000	38064000	31209000	20321000	63056000	42979000
Gm15448	1097700	470750	3139500	540430	642700	705390	2149500	156770	389030	386720	2279700	1257000
Il6st	1783600	2060600	0	3150000	1264000	1675700	2626100	1707000	1207100	1931400	3399800	2961300
Lrp1	178770000	119050000	219640000	117210000	168880000	108190000	193600000	149940000	90154000	80233000	214450000	140560000
Neo1	7006700	2820800	17118000	5285000	7624900	3792900	12459000	9662900	1801800	2329400	13791000	11717000
Nrcam	6575500	3840400	8845900	4261600	5464600	4166900	7145200	5318800	2743800	2222500	10252000	4468800
Trpv2	3178700	1491600	1491000	1341000	3004000	1107100	1607500	1329100	1467400	0	4427500	0
Apobr	1406300	3740900	1890500	6167200	0	3285500	2311600	2201900	1769100	3196900	1733500	3070600
Cd180	8861400	7988800	6456700	14707000	6823500	9161000	4043500	4314800	5048500	2331200	9307500	2181000
Tspo	5207100	4103000	3660500	7521000	2952600	4005900	1633100	1107400	1524100	1227200	2988900	577020
Mpp1	5109900	7383800	6745700	8320500	7838400	6661500	5497800	3002600	5465600	6325800	5702500	6354200
Snx9	2653500	1686500	2292300	0	1986300	1597800	0	2142400	1857800	2064000	1723400	2090900
Stk10	1474700	2321000	1387200	2595300	1126800	1202900	1013900	0	0	1078400	1245800	1204700
Apmap	993300	1231400	1160600	4168900	951080	1217900	0	0	0	1301000	0	0
Lgals3	150570000	262720000	194200000	345390000	213440000	250610000	157260000	175870000	215020000	250590000	138830000	190070000
Cse1l	27700000	27867000	20577000	32612000	19728000	20224000	14555000	19157000	20790000	21892000	19944000	11775000
Dnaja2	9273700	11825000	14740000	17000000	8790600	13635000	5208300	7248700	9204900	11897000	8238400	6914300
Anxa5	170480000	308090000	187920000	331330000	174600000	228950000	154800000	126090000	205750000	256070000	126400000	188400000
Ipo9	4464700	3843600	4554200	5051600	3018000	4472400	1948500	2317700	3110500	3791900	3137600	1388900
Plek	8635200	12689000	3632400	15386000	7806000	8228100	2775700	2433300	7610200	5953200	4998100	3253000
Lat2	1867900	2654100	2282300	3538100	1937900	3097800	0	0	1852700	2334300	2320000	2927800
Ap2a2	1458700	1881800	1541200	1425900	1563800	1540200	1474500	1011400	1474600	1447200	0	1450300
Sphk2	839930	929660	938280	1085300	737910	1211200	0	0	0	901060	894970	0
Tm9sf3	827980	2273200	1113900	1033000	633070	1014600	0	0	558480	758870	909660	0
Ano6	2895900	2490600	2636300	2758700	2567200	2811900	3666000	2920100	3674300	2630900	2473600	2772000
Vim	130210000	50727000	149310000	152270000	263010000	107490000	279000000	352000000	287750000	279440000	103000000	111410000
Pls3	5565100	10815000	5560400	10753000	6315500	9324000	4935900	3881000	9436300	8738500	4716200	4487100
S100a6	16674000	23677000	9500500	17308000	13113000	21995000	8966200	12871000	15318000	9920000	7783100	8498400
Clic1	99421000	146750000	81203000	99879000	83911000	88590000	81061000	92520000	108640000	115770000	76691000	85041000
Cpne1	2887300	4652100	2309600	2565600	3149600	2789900	2738400	2325400	3887800	4113100	1643500	3076100
Gnb2l1	116580000	99868000	92762000	75223000	106440000	88618000	98087000	80906000	104370000	103270000	115040000	97357000
Mesdc2	1533900	2504200	1230000	1713200	1528400	1637000	1394400	1438800	1551900	1332900	0	1389700
Pdap1	1769400	1240700	0	2897400	1588900	1961600	1347400	0	2625000	3292700	829750	1386600
Alcam	58710000	38305000	75313000	31607000	48484000	41980000	77539000	74955000	41911000	32820000	95880000	67948000
Lilrb4	182390000	85851000	167000000	65652000	122970000	86278000	186010000	152300000	148440000	87972000	217120000	215430000
Atp1b3	115710000	64771000	121750000	52231000	86643000	46698000	101950000	110130000	70863000	39049000	132460000	91666000
C3ar1	3843900	2312300	3067400	0	2448900	2392300	5393000	3184000	2717500	2063800	4796100	2580300
Cd44	45784000	36075000	70653000	25622000	50693000	30436000	89153000	79257000	29681000	20419000	77862000	39848000
Cd72	31111000	18321000	49949000	9747400	25692000	15769000	60925000	95367000	34350000	28215000	43115000	72792000
Clec12a	4782700	2168400	9996100	1572800	4786900	3366900	8978000	7448100	2887900	1380500	12569000	7619200
Cr1l	3522300	1934700	7351700	1377100	2853200	1633600	8867000	5916600	1857000	1098300	6490300	4611100
Evi2a	9980400	7086300	11506000	6641300	7339300	6561600	11461000	11426000	7741200	6389600	13235000	12330000
Evi2b	17346000	10642000	20310000	7124700	11535000	7746200	28334000	10037000	4309200	2546000	21714000	7894600
Fcgr1	40868000	21258000	34165000	15379000	32790000	25550000	58188000	61527000	26662000	14062000	49778000	53695000
Fcgr2;Fcgr2b	71470000	38623000	68049000	47908000	55487000	46529000	88875000	110640000	58118000	52119000	91780000	102620000
Flrt2	4348400	1750900	6333200	1737500	2077300	1751100	4621500	6870100	1646300	2823900	5271500	3226200
Igsf8	136370000	76787000	124740000	44107000	85975000	46563000	184610000	189250000	65917000	28824000	190720000	195040000
Itga4	5230800	2325400	9997300	4839900	5166300	2415500	8826000	10572000	2768000	3074500	6527500	6278500
Itgal	3228500	1362500	1396000	1123800	2052900	1007300	1862000	3322000	1457100	1072300	2113000	1595400
Itgam	43038000	56476000	52780000	61296000	41790000	46100000	55507000	69961000	39885000	44149000	59520000	47062000
Itgb1	75963000	102280000	75625000	82159000	49782000	79687000	85548000	182360000	54016000	68334000	93713000	135440000
Itgb2	57243000	56276000	56984000	52725000	54134000	41857000	66532000	79691000	44496000	46274000	67340000	55714000
Lphn2	5722800	1809000	5113400	1474800	2523900	2349800	5954800	6641700	2030100	1625400	6359800	2792400
Notch2	10051000	4606700	11924000	4154600	6834500	4144300	13229000	14164000	5924100	3829300	12287000	13276000
Nrp2	19923000	10365000	21444000	6811800	16219000	8815600	24816000	34155000	16094000	12745000	26291000	35802000
Plxdc1	13057000	6955600	27214000	7946900	12590000	6830700	19776000	22307000	7408500	6399200	14407000	18879000
Plxna1	24027000	16520000	22890000	13040000	18625000	13403000	29045000	24737000	15302000	16783000	30047000	24371000
Plxnb2	262640000	168070000	340790000	137130000	243040000	169390000	421710000	404130000	185250000	141690000	416360000	342440000
Pvr	2117900	1812000	2546500	2145700	2137900	1511000	2860500	2974200	1863100	1612000	2404800	2389200
St14	2865800	2171300	4895800	2052200	2043700	1775000	5986100	3711900	1958100	1664900	3358400	2519300
Tlr2	9307400	3468300	12720000	4059300	7334500	4573100	11248000	14021000	6459400	4734900	12738000	12106000
Itga5	352110000	158170000	315490000	111490000	192730000	146390000	383550000	400070000	226380000	151590000	376720000	414930000
Nrp1	154960000	74785000	205610000	71719000	125470000	85004000	213050000	215540000	94196000	66175000	255900000	249340000
	3257722310	2524859710	3390285680	2420160730	2796519560	2221459890	3684576200	3847443670	2572272570	2262956460	3678404680	3441599420
